# Probing Structural Diversity in a Series of Perhydrocarbyl Heterobimetallic Complexes Associating Tantalum and 3d (Cr, Mn, Fe, Co, Ni) Transition Metals

**DOI:** 10.1021/acs.inorgchem.5c02380

**Published:** 2025-10-17

**Authors:** Till Neumann, Iker Del Rosal, Laurent Maron, Erwann Jeanneau, Vincent Maurel, Serge Gambarelli, Jean-Marie Mouesca, Chloé Thieuleux, Clément Camp

**Affiliations:** † Laboratory of Catalysis, Polymerization, Processes and Materials (CP2M, UMR 5128), CNRS, 27098 Université Claude Bernard Lyon 1, CPE Lyon, Institut de Chimie de Lyon, 3 rue Victor Grignard, 69616 Villeurbanne, France; ‡ LPCNO (UMR 5215), CNRS & INSA, Université Paul Sabatier, 135 avenue de Rangueil, 31077 Toulouse, France; § Centre de Diffractométrie Henri Longchambon, Université Claude Bernard Lyon 1, 5 rue de la Doua, 69100 Villeurbanne, France; ∥ 70610 Université Grenoble Alpes, CEA, CNRS, INAC, SyMMES, F-38000 Grenoble, France

## Abstract

Salt metathesis between an alkali-stabilized tantalate complex, [Li­(thf)_2_]­[Ta­(C*t*Bu)­(CH_2_ *t*Bu)_3_] (**1**), and a series of alkylcyclopentadienyl 3d transition metal halide dimers, [Cp’M­(μ-X)]_2_ (**2-MX**, Cp’ = 1,2,4-*t*Bu_3_C_5_H_2_, X = Cl, Br or I), provides a set of perhydrocarbyl-stabilized heterobimetallic complexes [Ta­(CH_2_ *t*Bu)_2_(μ-CH*t*Bu)_2_MCp’]/[Ta­(CH_2_ *t*Bu)_3_(μ-C*t*Bu)­MCp’] (**3-M**, M = Cr, Mn, Fe, Co, Ni). A combination of single-crystal X-ray diffraction, spectroscopic studies and computational investigations reveals that the species **3-M** make up a series of nearly isostructural molecules, some of which exhibit unusual coordination geometries and an atypical α-hydrogen tautomerism. Most of these Ta–M pairs are rare or unprecedented in bimetallic molecular compounds and were designed as potential alternatives to analogous compounds in which tantalum is combined with more costly, heavy transition metals such as iridium or osmium, recently reported to be suitable precursors for Surface OrganoMetallic Chemistry (SOMC) and catalytic H/D exchange reactions.

## Graphical abstract

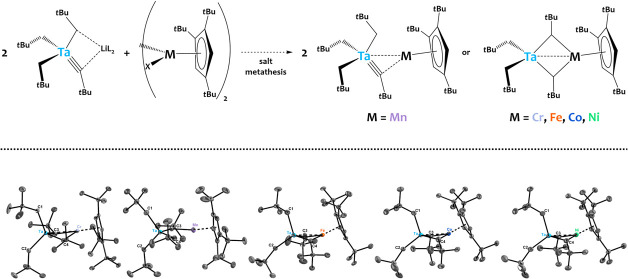

## Introduction

The assembly of two metal atoms with different chemical properties as parts of heterobimetallic complexes is of increasing interest to the organometallic chemistry community.^1–3^ Chemical cooperation between the metal centers has proven to be a powerful tool to achieve reactivities surpassing the performance of comparable monometallic compounds, especially with regard to the cleavage of strong chemical bonds. Numerous bimetallic complexes activating, for example, C–H,^4–16^ H–H,^17–31^ or C–O^32–42^ bonds stoichiometrically or catalytically have been reported so far. Although a few prevailing principles in the design of such bimetallic systems, namely the combination of metals with diverging HSAB characteristics and electronegativities and/or strong electronic interaction due to short metal–metal distances, seem to be beneficial, a judicious and purposeful selection of suitable metal centers and ligand frameworks remains challenging. Bridging phosphinoamide^43^ or phosphinopyrrolide^44^ ligands as well as tris­(phosphino)­borane/alane/gallane metalloligands^45–48^ and other multidentate tripodal ligand scaffolds^49–54^ are particularly versatile and have been shown to host a variety of different metal–metal pairs in nearly unmodified overall molecular structures (Scheme 1). The resulting series of structurally almost identical heterobimetallic complexes often enable in-depth investigations of the impact of a specific metal center on certain structural parameters (metal–metal bond order/polarity/length) and on the reactivity. However, while many of these compounds activate small molecules, their synthesis and stability usually rely on highly directing and sterically demanding ligands coordinating to the metal centers through heteroatom donors.

**Scheme 1:**
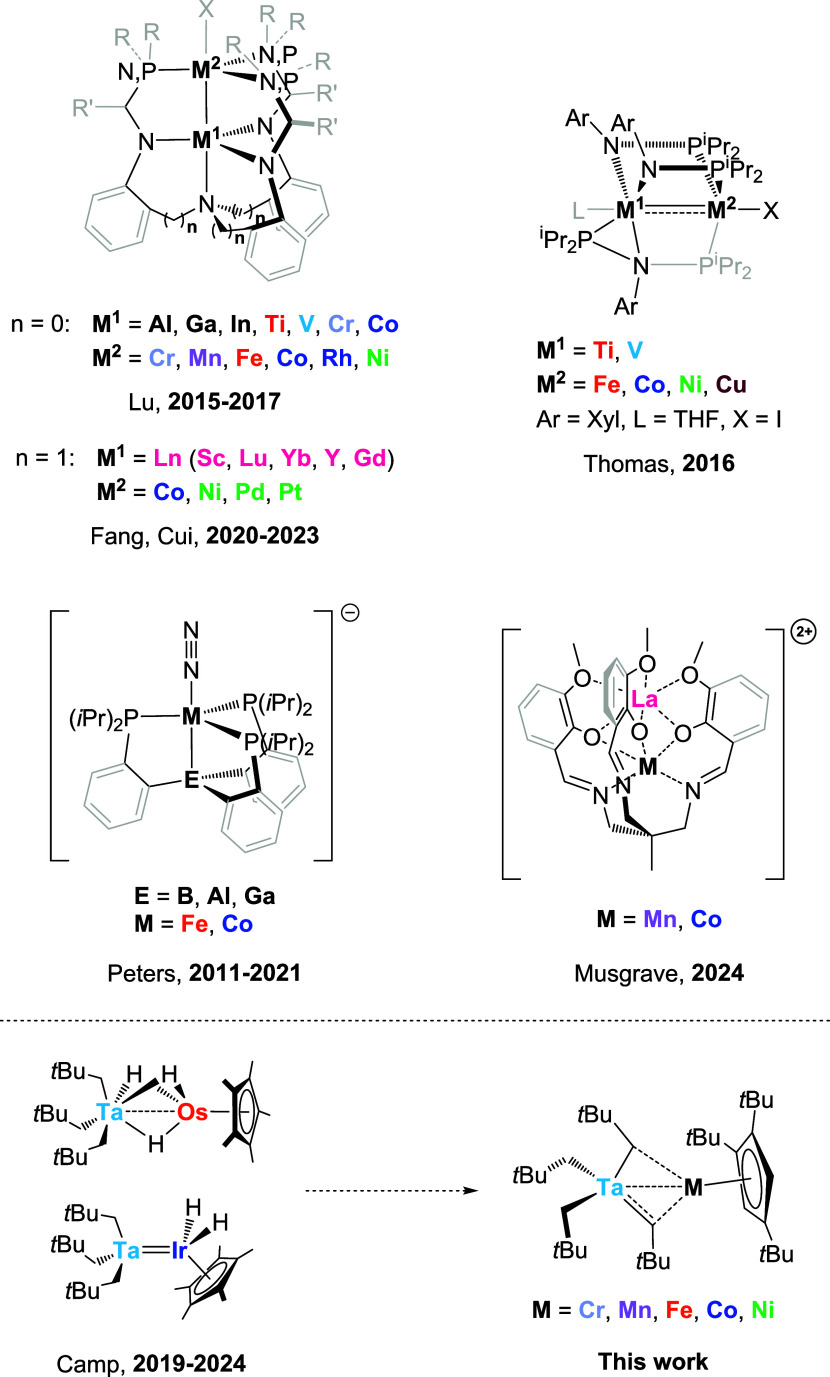
Isostructural Series of Heterobimetallic Complexes Stabilized by Multidentate Ligands Containing Heteroatom Donors (Top) and Perhydrocarbyl-stabilized Examples (Bottom)^6,42–54^

The synthetic pathway toward the series of complexes discussed in this report follows a different strategy. Instead of a preassembled ligand system “hosting” the two metal centers at well-defined coordination sites, salt metathesis between anionic and cationic monometallic fragments was developed to access heterobimetallic species associating tantalum and five different 3d transition metals in identical ligand environments. Two types of perhydrocarbyl ligands are used to stabilize the Ta–M pairs (M = Cr, Mn, Fe, Co, Ni): 2,2-dimethylpropyl (“neopentyl”) ligands prevent any β-hydride eliminations from the Ta center whereas the bulky 1,2,4-tri*tert*-butylcyclopentadienyl ligand (Cp’) sterically shields the 3d transition-metal ion. The absence of any heteroatom donors belongs to the specific constraints imposed by the potential use of these complexes as precursors for Surface OrganoMetallic Chemistry (SOMC). SOMC with heterobimetallic species has been put to use successfully by our group to access highly reactive Hf–Ir^55,56^ and Ta–Ir^6^ catalysts immobilized on a partially dehydroxylated silica support. In those cases, similar ligand environments enable (1) facile attachment of the bimetallic complexes onto the solid support via protonolysis between an alkyl ligand located at the early, oxophilic transition-metal center and a hydroxy group on the silica surface and (2) pretreatment of the immobilized catalysts with hydrogen gas to transform them into even more reactive hydride species.

In order to replace the rare and costly iridium by 3d transition metals and to extend the range of available bimetallic compounds, the monometallic precursor complexes as well as the synthetic strategy needed to be adapted. We report the impact of these modifications on the structures of the heterobimetallic complexes and discuss specific differences within the series of new Ta–M species as a function of the 3d transition metal M, putting special emphasis on a highly unusual alkyl–alkylidyne/bis­(μ-alkylidene) tautomerism around the Ta center.

## Results and Discussion

The treatment of first-row transition-metal complexes [Cp’M­(μ-X)]_2_ (**2-MX**, MX = CrCl,^57^ FeI,^58^ CoI,^59^ NiBr^60^) or [Cp’M­(thf)­(μ-Cl)]_2_ (**2-MnCl**)^61^ with two equivalents of the lithium tris­(alkyl)­alkylidynetantalate **1** ^62,63^ in a pentane solution warming from −40 °C to room temperature affords the heterobimetallic species [Ta­(CH_2_ *t*Bu)_2_(μ-CH*t*Bu)_2_MCp’]/[Ta­(CH_2_ *t*Bu)_3_(μ-C*t*Bu)­MCp’] (**3-M**) in which the metal center M ranges from chromium through manganese, iron and cobalt to nickel, according to the monometallic precursor **2-MX** used in the reaction (Scheme 2). As illustrated by ^1^H NMR studies of the reaction progress in a solution of deuterated benzene (C_6_D_6_), reaction times vary as a function of the 3d transition metal M, extending from a few minutes for M = Mn, Fe, Ni (Figures S17, S18, S20) to several hours in the case of M = Cr, Co (Figures S16, S19). All salt-metathesis reactions are accompanied by a color change and the precipitation of the corresponding lithium salt from the apolar reaction medium. Filtration and recrystallization from minimum amounts of an apolar solvent at −40 °C yield the pure bimetallic complexes in single-crystal quality in varying yields (29 to 89%, Scheme 2). Quite surprisingly, **3-Cr** was found to be less soluble than its Mn, Fe, Co and Ni counterparts and could be crystallized from either *n*-pentane or hexamethyldisiloxane (HMDSO), whereas HMDSO was required to obtain crystals of **3-Mn**, **3-Fe**, **3-Co** and **3-Ni**.

**Scheme 2:**
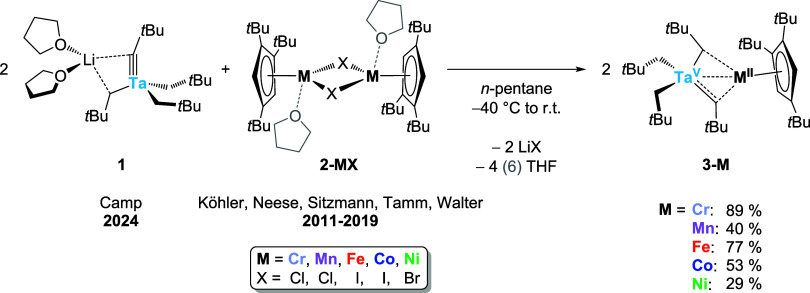
Synthesis of a Series of Heterobimetallic Complexes **3-M** via Salt Metathesis from Monometallic Precursors

The solution-state stability of complexes **3-M** over time was investigated using ^1^H NMR (Figures S16–S20) and UV–vis spectroscopies (Figures S21–S25). **3-Cr** and **3-Mn** are stable over prolonged periods of time, showing no signs of decomposition in the first 24 h after dissolution in benzene (NMR) or pentane (UV–vis). On the contrary, **3-Fe**, **3-Co** and **3-Ni** decompose within several hours even in diluted solutions, as highlighted by the evolution of their UV–vis spectra (c = 40 μM) and the diamagnetic decomposition products observed via ^1^H NMR, of which only neopentane (δ = 0.90 ppm) could be identified with certainty. The relative instability of **3-Fe**, **3-Co** and **3-Ni** explains the tendency toward lower synthetic yields with respect to **3-Cr** (see Experimental Section).

The crystal structures of compounds **3-M**, determined by X-ray diffraction, are shown in Figure 1. **3-Fe**, **3-Co** and **3-Ni** incorporate half an equivalent of HMDSO in their crystal structures. Those molecules crystallizing without any solvent molecules in the structure (**3-Cr**, **3-Mn**) belong to different settings (**3-Cr**: *C*2/*c*, **3-Mn**: I2/a) of the same space group (no. 15) while those compounds integrating half a molecule of HMDSO per asymmetric unit (**3-Fe**, **3-Co**, **3-Ni**) crystallize in the space group *P*2/*c* (no. 13). Numerous attempts were made to crystallize all compounds under identical conditions, but they consistently resisted adopting the same space group in the solid state. The unit cell parameters within these two isostructural groups are almost identical (Tables S2–3), and all five compounds are structurally related (Table 1). Although the series is marked by a number of surprising structural differences (see discussion below), all structures share some common features, including notably the similar coordination environments of the metal centers. The tantalum center is coordinated by the four α-carbon atoms of the surrounding alkyl and alkylidene ligands in **3-Cr**, **3-Fe**, **3-Co** and **3-Ni**. **3-Mn** stands out by the fact that three of the surrounding ligands are alkyl ligands while only one (alkylidyne) ligand formally bridges the metal centers (see discussion below). Generally, though, the donor atoms of the two terminal neopentyl ligands (C1, C2) and of the two neopentylidene ligands bridging Ta and the 3d transition-metal center (C3, C4) constitute a nearly regular tetrahedral coordination geometry with C–Ta–C angles deviating only slightly from the idealized angle, 109.5°. Each 3d transition-metal center in the **3-M** series is coordinated by the bridging alkylidene carbon atoms C3 and C4 and the substituted cyclopentadienyl ligand. Considering the C3–M–C4 angle comprised between 84.40(6)° (M = Cr) and 97.8(2)° (M = Ni) and the angles between C3/C4, M and the centroid of the Cp’ ligand, which range from 129.5(1)°/131.1(2)° (M = Ni) to 137.19(6)°/136.51(5)° (M = Cr), the trigonal arrangement is distorted in shape but virtually planar because the three angles add up to values between 357 and 359°.

**Figure 1:**
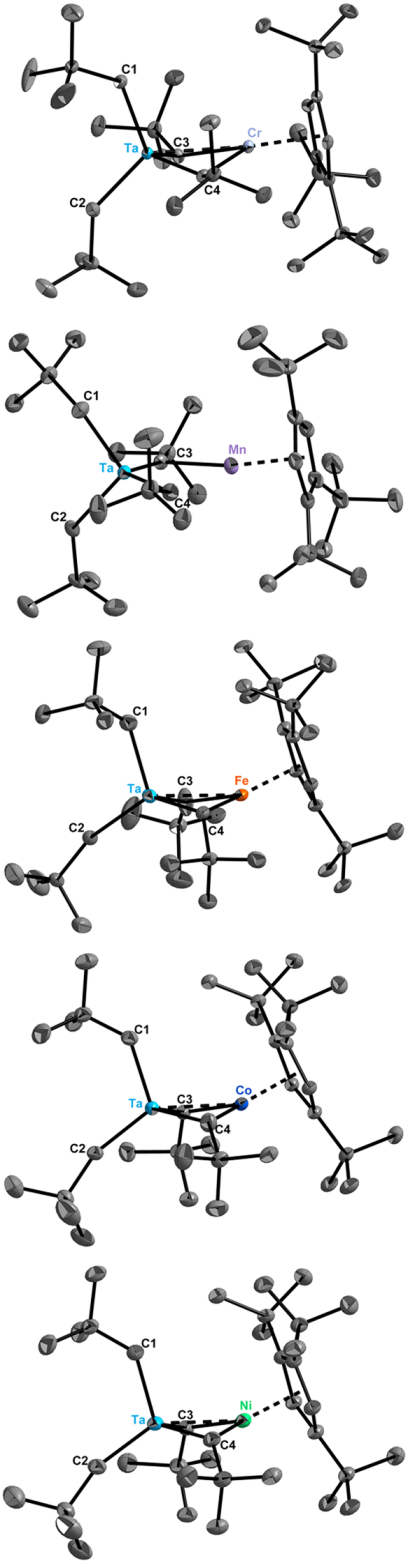
Solid-state molecular structures of **3-Cr**, **3-Mn**, **3-Fe**, **3-Co** and **3-Ni** (from top to bottom) with thermal ellipsoids (ORTEP) drawn at the 50% probability level. Carbon atoms except C1–C4 unlabeled and hydrogen atoms omitted for clarity. Selected bond distances and angles are listed in Table 1.

**Table 1:** Key Structural Parameters (Distances in Å and Angles in Deg) for Complexes **3-M** ^a^

	**3-Cr**	**3-Mn**	**3-Fe**	**3-Co**	**3-Ni**
Ta···M	2.8484(3)	3.015(1)	2.6406(8)	2.6196(4)	2.5773(4)
Ta–C1	2.163(2)	2.159(6)	2.174(6)	2.169(3)	2.157(3)
Ta–C2	2.145(2)	2.152(7)	2.071(6)	2.086(3)	2.081(3)
Ta–C3	2.010(2)	1.883(6)	2.017(7)	2.053(3)	2.035(3)
Ta–C4	1.995(2)	2.192(7)	2.055(5)	2.020(3)	2.019(3)
M–C3	2.249(2)	2.956(2)	2.066(7)	2.077(3)	2.050(3)
M–C4	2.323(2)	2.143(6)	2.105(6)	2.060(3)	2.030(3)
M···Cp’	2.017(3)	2.057(2)	1.878(7)	1.793(3)	1.819(3)
Ta–C1–*t*Bu	129.5(2)	135.1(5)	126.2(4)	126.2(2)	127.5(2)
Ta–C2–*t*Bu	133.2(2)	133.1(5)	161.4(4)	161.4(2)	162.0(2)
Ta–C3–*t*Bu	121.1(1)	153.7(5)	136.4(5)	127.4(2)	128.3(2)
Ta–C4–*t*Bu	133.1(1)	119.9(4)	128.5(4)	134.5(2)	133.3(2)
C1–Ta–C2	114.24(7)	109.9(3)	108.9(2)	108.0(1)	108.5(1)
C1–Ta–C3	113.25(7)	108.4(3)	106.7(3)	113.2(1)	112.6(1)
C1–Ta–C4	114.06(7)	111.6(2)	112.3(2)	106.5(1)	106.6(1)
C2–Ta–C3	107.53(7)	107.5(5)	114.8(3)	114.9(1)	114.8(1)
C2–Ta–C4	106.44(7)	111.0(3)	115.0(2)	115.1(1)	115.3(1)
C3–Ta–C4	100.13(7)	111.7(3)	98.7(3)	98.8(1)	98.6(1)
C3–M–C4	84.40(6)	81.2(4)	95.6(3)	96.8(2)	97.8(2)
C3–M–Cp’	137.19(6)	161.6(3)	132.5(2)	129.54(9)	129.5(1)
C4–M–Cp’	136.51(5)	116.5(3)	129.4(2)	131.3(2)	131.1(2)

a Cp’ Denotes the Centroid of the Carbon Atoms Making up the Five-membered Ring of the Cyclopentadienyl Ligand.

Despite these structural similarities, the crystallographic parameters also reveal a number of differences between the bimetallic complexes (Table 1). Some of the Ta–M distances vary considerably, especially in the cases of **3-Cr** (*d*_Ta–Cr_ = 2.8484(3) Å) and **3-Mn** (*d*_Ta–Mn_ = 3.015(1) Å), while those in the last three members of the series, **3-Fe**, **3-Co** and **3-Ni**, are much closer to each other and significantly shorter (*d*_Ta–Fe_ = 2.6406(8) Å, *d*_Ta–Co_ = 2.6196(4) Å, *d*_Ta–Ni_ = 2.5773(4) Å). Cotton’s formal shortness ratio (FSR)^64^ was used as a normalization method in order to evaluate the metal–metal distances independently of the decreasing atomic radii in the first row of the *d*-block. The FSR is defined as the ratio of the crystallographically observed distance *d* between two metals to the sum of their metallic radii as defined by Pauling.^65^ It substantially exceeds unity in **3-Cr** (FSR = 1.13) and **3-Mn** (FSR = 1.20), thus ruling out any covalent bonding between the metal centers. Regarding **3-Fe** (FSR = 1.05), **3-Co** (FSR = 1.05) and **3-Ni** (FSR = 1.03), the FSR are only slightly larger than 1 and represent a borderline case in which a certain covalent interaction is plausible according to some authors,^2^ even though the presence of genuine metal–metal bonds is not suggested here.

Apart from some Ta-based cluster compounds containing three or more metal centers,^66–69^ there are only very few examples of bimetallic complexes associating Ta and Cr (0), Mn (1), Fe (2), Co (2) or Ni (2) in the literature (Scheme 3). The Ta–Mn and Ta–Fe compounds reported by Bergman and co-workers^70^ were obtained via reactions of [Cp_2_Ta­(=CH_2_)­(CH_3_)] with Mn and Fe carbonyl complexes and show comparable Ta–M distances (M = Mn: 2.931(1) Å, M = Fe: 2.880(1) Å) lying between those observed in **3-Mn** (3.015(1) Å) and **3-Fe** (2.6406(8) Å). Incidentally, the Ta–Fe contacts in the *cyclo*-P_5_ and *cyclo*-As_5_ triple-decker complexes described by Scherer and co-workers^71,72^ roughly correspond to that in Bergman’s example and are therefore also longer than that in **3-Fe** by more than 0.2 Å. However, the most pertinent comparison can be made between two Ta–Co complexes reported by Bergman and co-workers^73,74^ and **3-Co** due to the structural resemblance of the Ta­(μ-CHR)_2_Co cores featuring two bridging alkylidene ligands. Both literature-known values for Ta–Co distances exceed the one in **3-Co** by approximately 0.1 and 0.2 Å, respectively. Only the Ta–Ni compounds developed by the groups of Tsurugi & Mashima (L = IPr)^75^ and Tonks^44^ exhibit metal–metal distances comparable to or shorter than those in **3-Ni** or any of the other bimetallic species reported here. In those two cases, the authors propose dative, late-to-early bonding between an electron-rich Ni^0^ and an electron-deficient Ta^V^ center.

**Scheme 3:**
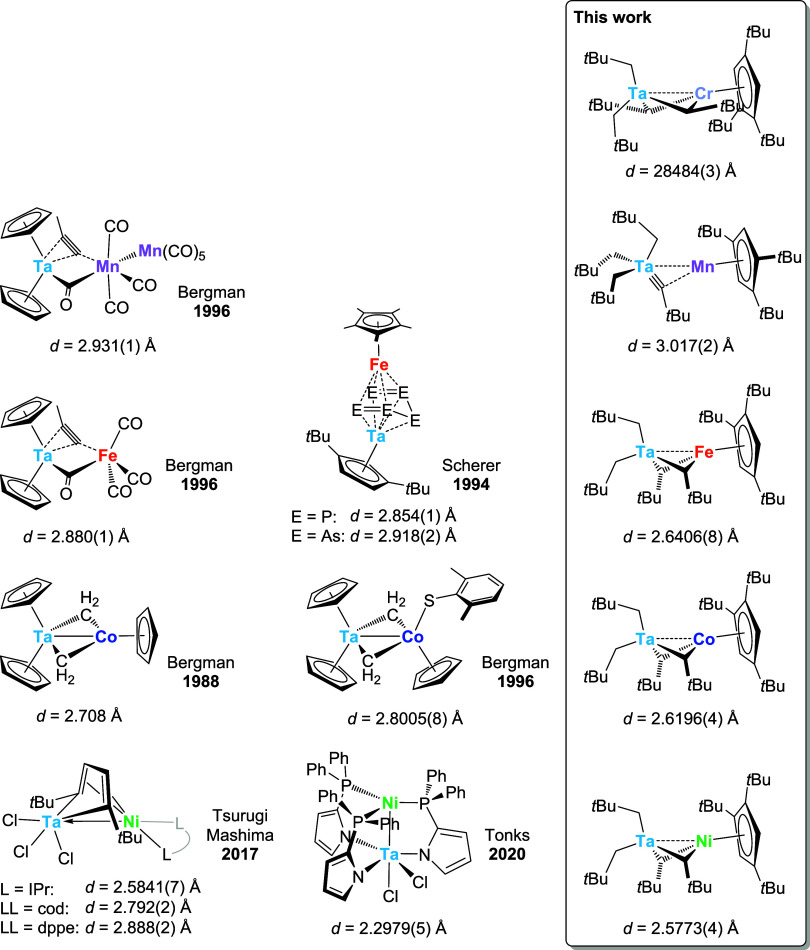
Ta–M (M = Cr, Mn, Fe, Co, Ni) Complexes and Associated Metal–Metal Distances Reported to Date

Further differences between the bimetallic complexes **3-M** exist with regard to the bond lengths and angles around the α-carbon atoms of the terminal alkyl ligands and with regard to the spatial orientation of the bridging alkylidene ligands (Table 1). In **3-Cr**, the Ta–C1 and Ta–C2 distances (2.163(2) and 2.145(2) Å) and Ta–C1–*t*Bu and Ta–C2–*t*Bu angles (129.5(2)° and 133.2(2)°) are expected parameters for alkyl ligands featuring sp^3^-hybridized α-carbon atoms and Ta–C single bonds. They correspond to those previously reported for Ta complexes containing neopentyl ligands, such as **1**,^63^ the closely related [(dmp)­Li]­[Ta­(C*t*Bu)­(CH_2_ *t*Bu)_3_] (dmp = *N*,*N*′-dimethylpiperazine)^62^ and a variety of monometallic and bimetallic examples previously described by our group.^55,76,77^ The *t*Bu groups of the bridging neopentylidene ligands point toward the convex face of the Ta­(μ-CHtBu)_2_Cr moiety formed by the two metal centers and atoms C3 and C4, leading to the “W-shaped” arrangement represented in Scheme 4 if viewed along the metal–metal axis. On the contrary, while complexes **3-Fe**, **3-Co** and **3-Ni** feature expected Ta–C1 distances (2.15–2.18 Å) and Ta–C1–*t*Bu angles (126°–128°), their Ta–C2 distances are shortened (2.07–2.09 Å) and their Ta–C2–*t*Bu angles widened (161°–163°). These parameters are closer to those typically found in Ta neopentylidene complexes, even though the values reported in the literature vary widely from 1.93 to 2.04 Å and from 140 to 169°, respectively.^78–81^ Moreover, the *t*Bu substituents of the bridging alkylidene ligands are directed toward the concave face of the Ta­(μ-CHtBu)_2_M moieties, forming a “flipped U” if viewed along the metal–metal axis.

**Scheme 4:**
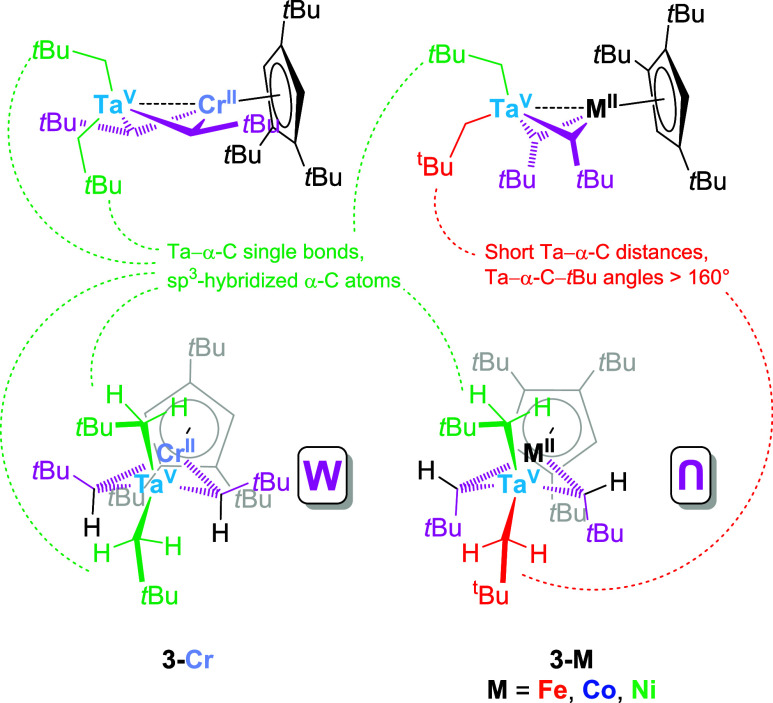
Differences between **3-Cr** and **3-M** (M = Fe, Co, Ni) Regarding the Neopentyl α-Carbon Atoms (Typical Arrangements in Green, Unusual Features in Red) and the Spatial Orientations of the *t*Bu Substituents Belonging to the Bridging Neopentylidene Ligands (Pink)

Interestingly, **3-Mn** is an exception in several respects. The Ta–Mn complex and its solutions in noncoordinating solvents are red, as opposed to the other bimetallic compounds in the series, which are black. Its crystal structure reveals by far the longest metal–metal distance (3.015(1) Å) and the highest FSR (1.20) of all complexes **3-M**. As in **3-Cr**, the terminal neopentyl ligands at the Ta center can be regarded as classical alkyl ligands with Ta–C bond lengths (2.155(4) Å) and Ta–C–*t*Bu angles (133.1(5) and 135.1(5)°) clearly indicating sp^3^-hybridized α-carbon atoms. However, C3 and C4 do not bridge the metal centers in the symmetrical way described for the other structures in the series. Instead of a regular Ta­(μ-CHtBu)_2_M core characterized by pairs of equal Ta–C and M–C distances, the Ta–C3 bond in **3-Mn** measures 1.883(6) Å and the corresponding Ta–C3–*t*Bu angle 153.7(5)°, values that lie between those usually observed for the formal Ta=C double and Ta≡C triple bonds in the examples of Ta neopentylidene^78–81^ and Ta neopentylidyne^62,63^ complexes discussed earlier, whereas the Ta–C4 distance (2.192(7) Å) and the Ta–C4–*t*Bu angle (119.9(4)°) are typical of an alkyl ligand bearing an sp^3^-hybridized α-carbon atom. Along with an elongated Mn–C3 distance of 2.956(2) Å, compared to 2.143(6) Å between Mn and C4, these structural parameters suggest that the tris­(alkyl)-alkylidyne tautomer A depicted on the left in Scheme 5, in which a formal tantalum–carbon multiple bond is π-coordinated to the manganese ion, is favored over the bis­(alkyl)-bis­(μ-alkylidene) tautomer B (right) adopted by the other bimetallic complexes **3-M** (M = Cr, Fe, Co, Ni).

**Scheme 5:**
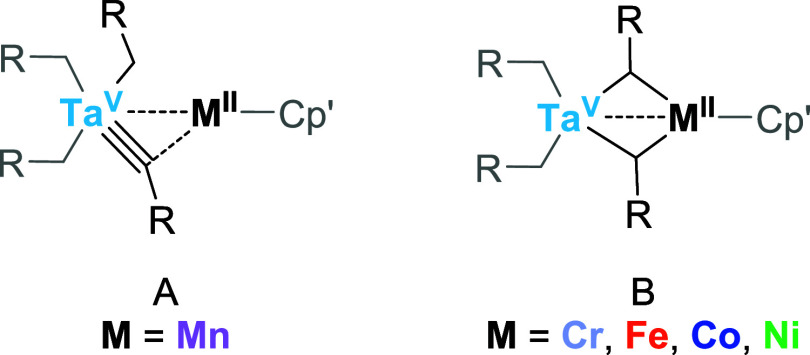
Experimentally Observed Tautomers: Tris­(alkyl)-alkylidyne Form (A), Which is Observed Only in the Case of **3-Mn**, and Bis­(alkyl)-bis­(μ-alkylidene) Form (B) Adopted by **3-Cr**, **3-Fe**, **3-Co** and **3-Ni**

Since the yield of crystalline product in the reaction between **1** and [Cp’Mn­(μ-Cl)­(thf)]_2_ (**2-MnCl**) giving **3-Mn** is lower (40%) than in the analogous syntheses of **3-Cr**, **3-Fe** and **3-Co** (89, 77 and 53%, respectively; see Scheme 2 and Experimental Section), **2-MnCl** was replaced by a similar precursor containing iodide instead of chloride ligands, [Cp’Mn­(μ-I)­(thf)]_2_ (**2-MnI**). Surprisingly, the reaction of **1** with **2-MnI** under the same reaction conditions does not afford **3-Mn** in higher yields. The product crystallizing from the reaction mixture after filtration is **3-Mn′** (47%), a Ta–Mn species whose anionic charge is balanced by a THF-coordinated lithium counterion (Scheme 6). In **3-Mn′**, one of the Mn centers from the monometallic precursor **2-MnI** retains both of its iodide ligands under displacement of the Cp’ ligand. It seems reasonable to assume that the expelled Cp’ ligand coordinates to the second Mn ion in **2-MnI** to give the corresponding manganocene [MnCp’_2_], a common byproduct in salt-metathesis reactions involving **2-MnI**.^61^ Unlike **3-Mn′**, [MnCp’_2_] could not be crystallized from the reaction mixture and was only identified *in situ* by its characteristic, broad ^1^H NMR resonance at 14.69 ppm (Figure S5).^82^ Initially, **3-Mn′** was obtained despite a reaction of **2-MnI** with two equivalents of **1** in the conditions of Scheme 2. After **3-Mn′** had been identified, the stoichiometry of the reaction was adjusted to a 1:1 ratio of **1** and **2-MnI** to increase the yield and avoid excess **1** in the reaction mixture. Unfortunately, the elemental analysis results of **3-Mn′** were unsatisfactory despite several attempts of purification, possibly due to partial decomposition through loss of THF molecules from the structure or trace contamination by the reaction byproduct (see Experimental Section). For this reason, it was tried to obtain **3-Mn′** from a reaction of **1** with the tetrahydrofuran adduct of manganese­(II) iodide, [MnI_2_(thf)_3_].^83^ This method yielded a third product, **3-Mn″**, closely related to **3-Mn′** by the identical anionic [Ta­(CH_2_ *t*Bu)_3_(μ-C*t*Bu)­MnI_2_]^−^ fragment contained in both species (Scheme 6). **3-Mn″** differs from **3-Mn′** in the fact that the lithium ion is coordinated by both manganese-bound iodide ligands and only two THF molecules instead of forming an isolated cation coordinated by four THF molecules. The reaction proceeds stoichiometrically, affording **3-Mn″** in single-crystal quality and in good yield (67%), avoiding MnCp’_2_ as a byproduct. Elemental analysis confirmed the purity of the product in this case. It should be noted that reactions of **1** with the tetrahydrofuran adducts of other 3d transition-metal halides such as [FeI_2_(thf)_2_] and [Co_3_I_6_(thf)_8_] result in intractable reaction mixtures of insoluble, black products which elude crystallization. These findings further highlight the exceptionality of the Ta–Mn bimetallic combination.

**Scheme 6:**
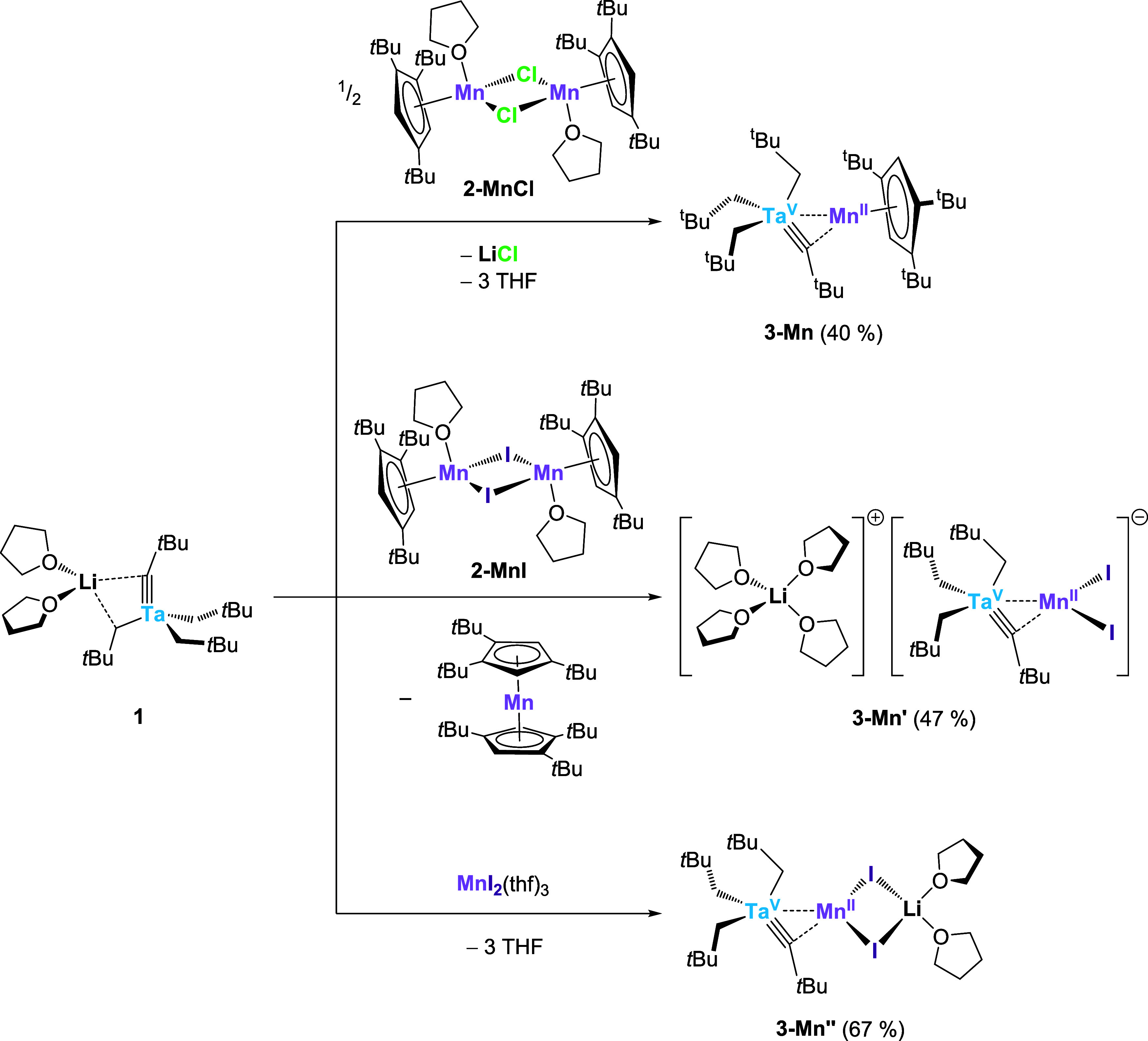
Influence of the Monometallic Mn Precursor on the Outcome of the Reaction with **1** under Otherwise Identical Reaction Conditions

Interatomic distances and angles in the solid-state structures of the heterobimetallic anions in **3-Mn′** and **3-Mn″** are virtually identical. On the one hand, the Ta–Mn contacts in **3-Mn′** and **3-Mn″** (2.838(1) and 2.8362(4) Å, respectively) are much shorter than that in **3-Mn** (3.017(2) Å) and therefore have a lower FSR of 1.13. On the other hand, the preference for a Ta tris­(alkyl)-alkylidyne moiety coordinated to the Mn center over a bis­(alkyl)-bis­(μ-alkylidene) species is a structural feature that **3-Mn**, **3-Mn′** and **3-Mn″** have in common and that distinguishes them from the other Ta–M complexes (M = Cr, Fe, Co, Ni). Like in **3-Mn**, three of four Ta−α-C distances (to carbon atoms C1, C2 and C3) in **3-Mn′** and **3-Mn″** are relatively long (2.145(2)–2.222(2) Å). Accordingly, the Ta−α-C–tBu angles range from 117.2(1) to 135.3(1)°, suggesting ordinary alkyl ligands. The distances between the Ta centers and the carbon atoms closest to the Mn ions (C4) are considerably shorter (1.863(6) and 1.863(1) Å, respectively) and the angles toward the quaternary carbon atoms of the corresponding tBu groups very wide (162.2(5) and 162.0(1)°), underlining the tantalum–carbon multiple-bond character in what can be formally described as a neopentylidyne ligand. Accordingly, the Mn–C3 distances (2.546(6) and 2.551(2) Å) exceed the Mn–C4 distances (2.234(6) and 2.183(2) Å) by more than 0.3 Å (Figure 2). In total, this leads to the unsymmetrical Ta­(CH_2_tBu)_3_(μ-CtBu)Mn motif encountered in the neutral derivative **3-Mn** earlier on, as opposed to the symmetric Ta­(μ-CHtBu)_2_M arrangements found in **3-Cr**, **3-Fe**, **3-Co** and **3-Ni**.

**Figure 2:**
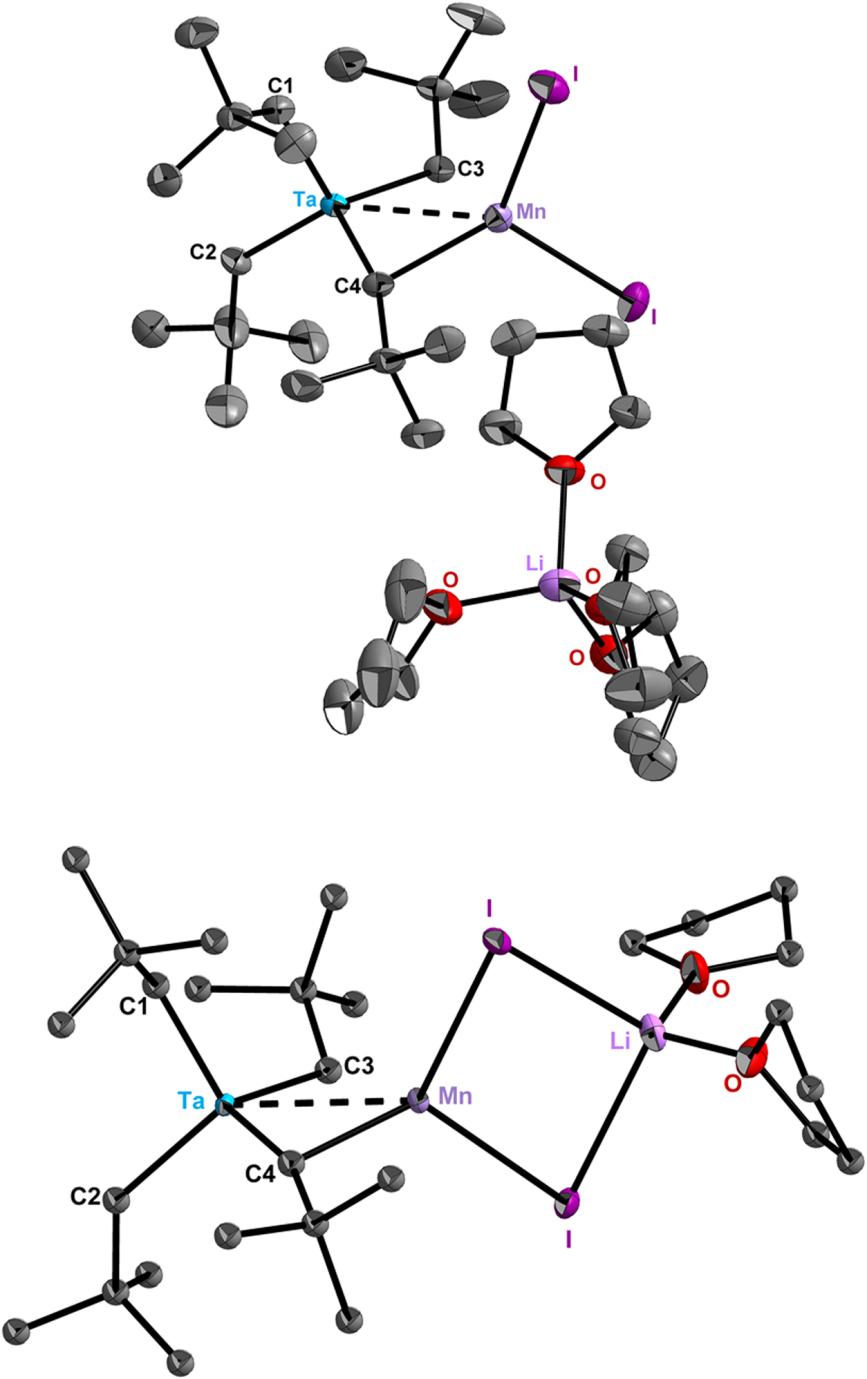
Solid-state molecular structures of **3-Mn′** (top) and **3-Mn″** (bottom) with thermal ellipsoids (ORTEP) drawn at the 50% probability level. Carbon atoms except C1–C4 unlabeled and hydrogen atoms omitted for clarity. Selected bond distances (Å) and angles (°) in **3-Mn′**/**3-Mn″**: Ta···Mn 2.838(1)/2.8362(4), Ta–C1 2.166(6)/2.145(2), Ta–C2 2.158(7)/2.160(2), Ta–C3 2.191(6)/2.222(2), Ta–C4 1.863(6)/1.863(1), Mn–I_(avg)_ 2.693(1)/2.7158(4), Mn–C3 2.546(6)/2.551(2), Mn–C4 2.234(6)/2.183(2); Ta–C1–*t*Bu 129.4(4)/135.3(1), Ta–C2–*t*Bu 135.1(5)/133.4(1), Ta–C3–*t*Bu 119.7(4)/117.2(1), Ta–C4–*t*Bu 162.2(5)/162.0(1).

Density-functional theory (DFT) calculations (Tables S4–S14, Figures S33–S35) were carried out to gain a deeper understanding of this unusual type of tautomerism. Alkyl–alkylidyne/bis­(alkylidene) rearrangements were first postulated by Schrock and co-workers to account for the formation of [CpTa­(CH*t*Bu)_2_(PMe_3_)] from treating [CpTa­(C*t*Bu)­(Cl)­(PMe_3_)_2_] with [Mg­(CH_2_ *t*Bu)_2_(dioxane)][Bibr ref84] and have since been documented in a small number of W and Os complexes.^85–90^ Most recently, such tautomeric equilibria were also detected in a set of bimetallic complexes associating Ta and the coinage metals (Cu, Ag, Au).^63^ Scheme 7 depicts the conceivable tautomeric forms of **3-M** which were thought to cause the unusually short Ta–C bonds and wide Ta–C–*t*Bu angles observed in many of the crystal structures. Their geometries and spin states were optimized using the B3PW91 hybrid functional with double-ζ quality basis set 6–31G­(d,p) on carbon and hydrogen atoms and Stuttgart effective core potential basis sets augmented with polarization functions on the metal centers. The energetically lowest geometries (Table 2) correspond to those found experimentally except for **3-Fe**, which is predicted to exist as the tris­(alkyl)-alkylidyne tautomer but crystallizes in the bis­(alkyl)-bis­(μ-alkylidene) form. It should be mentioned, however, that the geometry optimizations are performed at a different temperature (298 K) than the crystallization of the compounds (233 K) and that explicit solvation or crystal packing effects are not taken into account in these considerations.

**Scheme 7:**
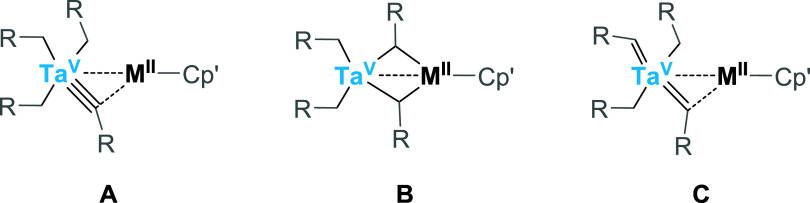
Possible Tautomeric Forms of a Generalized Complex **3-M**: (A) Tris­(alkyl)-alkylidyne, (B) Bis­(alkyl)-bis­(μ-alkylidene) and (C) Bis­(alkyl)-bis­(alkylidene) Form

**Table 2:** Electronic Structures of Compounds **3-M** ^a^

		**3-Cr**	**3-Mn**	**3-Fe**	**3-Co**	**3-Ni**
Δ_*r*_ *H (*Δ_*r*_ *G)*/kcal mol^–1^	**A**	4.2 (3.6)	0.0 (0.0)	0.0 (0.0)	1.4 (−0.4)*	6.3 (3.7)*
	**B**	0.0 (0.0)	3.3 (3.7)	3.1 (3.3)	0.0 (0.0)	0.0 (0.0)
	**C**	16.5 (15.9)	6.3 (6.8)	5.6 (6.1)	7.5 (4.5)*	13.1 (10.8)*
2S + 1 (calculated)		5	6	5	2 (4*)	1 (3*)
NMR (evans)	**μ**_**eff**_ **/μ**_**B**_	4.86	5.98	4.08	2.23	0.00
	***n***_**exp**_	4.0	5.1	3.2	1.4	0.0
DFT	**2S + 1**	5	6	5	2	1
	***n***_**comp**_	4	5	4	1	0
electr. configuration (oxidation state + II)		d^4^	d^5^	d^6^	d^7^	d^8^
		*S* = 2	*S* = 5/2	*S* = 2	*S* = 1/2	*S* = 0
M···Cp’ distances/Å	**2-MX**	1.95	2.13	1.93	1.70	1.79
		(*S* = 1 or 2)	(*S* = 5/2)	(*S* = 2)	(*S* = 1/2)	(*S* = 0)
	**3-M**	2.017(3)	2.05(2)	1.878(7)	1.793(3)	1.819(3)

a Computational relative electronic enthalpies (Δ_*r*_ *H*) and Gibbs Free energies (Δ_*r*_ *G*) of complexes **3-M** in the tautomeric forms A to C represented in Scheme 7 and calculated spin multiplicities of the optimized structures. The asterisk (*) indicates a spin-state change with respect to the most stable tautomer. Magnetic moments μ_eff_ measured using the Evans NMR method and computationally determined spin multiplicities 2S + 1 of complexes **3-M** along with the inferred numbers of unpaired electrons *n*_exp_/*n*_comp_ and a description of the most probable electron configurations at the 3d transition-metal centers. Crystallographic M···Cp’ distances in **2-MX** and **3-M** for comparison.

For all complexes **3-M**, the experimentally observed tautomers A (M = Mn) and B (M = Cr, Fe, Co, Ni) as well as the hypothetical intermediate form C lie within a relative energy range of 16 kcal mol^–1^, in most cases even within 6 kcal mol^–1^ (Table 2). The most favorable calculated spin multiplicity is the same for all tautomers of a given compound except for the A and C forms of **3-Ni** and **3-Co**, which show a preference for the triplet instead of the singlet state for the former and quartet instead of doublet for the latter.

Despite the small energetic differences between the optimized structures A, B and C of **3-M**, in-depth DFT studies suggest that the interconversion between these tautomeric forms is hampered by significant activation barriers in all cases. Energy profiles for the conversion of A to B were computed for a direct transformation from the tris­(alkyl)-alkylidyne form A to the bis­(alkyl)-bis­(μ-alkylidene) form B (Figure S34) and for an indirect reaction pathway through the intermediate bis­(alkyl)-bis­(alkylidene) form C (Figure S35). Overall, the reaction barriers exceed values normally expected for reactions occurring spontaneously at room temperature: barriers around 30 kcal mol^–1^ were computed for the passage from A to C and even higher barriers were found for the direct conversion from A to B (above 40 kcal mol^–1^).

Taking into account these additional investigations, the discrepancy between the computational and experimental geometries of **3-Fe** could also be explained by the fact that **3-Fe** (form B) is the kinetic product of the reaction between **1** and **2-Fe** and that a conversion into the thermodynamic product **3-Fe** (form A) is impossible. Accordingly, in all other cases (M = Cr, Mn, Co, Ni), the optimized and observed structures (**3-Cr**(B), **3-Mn**(A), **3-Co**(B), **3-Ni**(B)) would simultaneously be the kinetic and thermodynamic products.

Additional analytical techniques were employed, especially to confirm the correct description of **3-Fe**, **3-Co** and **3-Ni** as the B tautomer bearing two actual neopentyl ligands, in spite of the relatively short Ta−α-C bonds and exceptionally obtuse Ta−α-C–*t*Bu angles observed for one of the ligands in each crystal structure. Diffuse reflectance infrared Fourier-transform spectroscopy (DRIFTS) confirms the great similarity between all compounds **3-M** of the bimetallic series but allows no conclusion to be drawn about possible differences in the nature of a particular ligand or bond as all spectra share the same characteristic features. Among them are some very intense, overlapping absorption bands between 3000 and 2800 cm^–1^ that are assigned to the C–H stretching modes of the molecules’ methyl groups, the absence of any intense bands in the region between 2800 and 1500 cm^–1^ due to the lack of heteroatoms and hydrides in the ligand scaffold, and a close resemblance of the fingerprint regions ranging from 1500 to 500 cm^–1^ (Figures S26–S30).

Nuclear magnetic resonance (NMR) studies are of limited use for most of the complexes because of their paramagnetism. The ^1^H NMR spectra of **3-Cr**, **3-Mn**, **3-Fe** and **3-Co** are either completely featureless or exhibit only a small number of broad resonances (Figures S1, S3, S9, S11). Only **3-Ni** is a diamagnetic compound. Consequently, its ^1^H, ^13^C and ^1^H–^13^C heteronuclear single-quantum coherence (HSQC) spectra (Figures S13–S15) provide valuable insight into the bonding situation in the perhydrocarbyl ligands. Most importantly, the ^1^H NMR spectrum shows four pairs of chemically equivalent protons. Given the molecular structure of **3-Ni**, the most downfield resonance at 5.83 ppm can be attributed to the two aromatic protons of the trisubstituted cyclopentadienyl ligand. Each of the other three resonances integrating for two protons is explained by one of the α-proton sets in the alkyl and alkylidene ligands. Related examples of diamagnetic early-late heterobimetallic complexes featuring μ-alkylidene ligands are limited to some Ta­(μ-CH_2_)Ir and Ta­(μ-CH_2_)Pd compounds reported by Bergman and co-workers^91–93^ and to the neopentylidene-bridged Ta–Rh and Ta–coinage metal complexes recently described by our group.^63,77^ In the former species, the metal centers are bridged by two methylene ligands with ^1^H chemical shifts (3.96 to 7.22 ppm) varying over wide ranges and ^13^C chemical shifts (74.5 to 163.0 ppm) generally lying between the values observed for the terminal methyl (δ­[^13^C] = 5 ppm) and methylene (δ­[^13^C] = 224 ppm) ligands of the monometallic Ta precursor, [Cp_2_Ta­(CH_2_)­(CH_3_)], from which they are obtained.^94^ The same is true of the bridging CH moieties in the literature-known neopentylidene complexes (δ­[^1^H] = 4.78, δ­[^13^C] = 170.1 ppm in the Ta–Rh complex;^77^ δ­[^1^H] = 2.19, δ­[^13^C] = 167.6 ppm in the Ta–Au species^63^) when compared to the associated monometallic precursor, [Ta­(CH*t*Bu)­(CH_2_ *t*Bu)_3_] (α-CH_2_: δ­[^13^C] = 113.7; α-CH: δ­[^13^C] = 250.1 ppm).^95^ Hence, the α-^1^H and α-^13^C chemical shifts of **3-Ni** strongly suggest that the singlet at 4.15 ppm showing a ^1^H–^13^C single-bond correlation with the most downfield ^13^C resonance at 187.5 ppm most probably belongs to the α protons of the two bridging neopentylidene ligands. The two upfield ^1^H resonances at 0.50 and 0.61 ppm feature ^1^J_C–H_ correlation signals with the ^13^C resonances at 104.2 and 146.1 ppm, respectively. These chemical shifts are rather typical of α-hydrogen and α-carbon atoms in classical, terminal alkyl ligands and are therefore assigned to the α-positions of the Ta-bound terminal neopentyl ligands in **3-Ni**. In this context, it should be noted that the two μ-alkylidene ligands in **3-Ni** are chemically equivalent whereas the two alkyl ligands are not. This finding reflects both the symmetrical arrangement of the bridging neopentylidene ligands around the metal–metal axis and the structural differences between the two terminal neopentyl ligands observed in the solid-state molecular structure (Figure 1).

Furthermore, NMR spectroscopy provides some indirect information on the oxidation states of the metal centers. On the one hand, the oxidation states of the metal ions in the monometallic precursors **1** and **2-MX** are +V in the case of Ta and +II for the 3d transition metals. On the other hand, taking into account the coordination environments in **3-M**, the tetracoordinate Ta center could be formally viewed as a Ta­(+IV) and the tricoordinate 3d transition-metal centers classified as M­(+III) ions. However, the diamagnetic behavior of **3-Ni** is strongly in favor of a closed-shell 3*d* ^8^ configuration of the nickel center and thus the oxidation state +II, hinting at a salt metathesis reaction between **1** and **2-NiBr** that occurs without any electron transfer from one metal center to the other. It is assumed that the same attribution of oxidation states also holds true for the paramagnetic complexes of the bimetallic series. In particular, EPR studies were performed on compound **3-Mn**, which structurally differs from the other compounds in the series, confirming its S = 5/2 spin state (Mn^+II^), with the majority of the spin density localized on the Mn center (see SI). This hypothesis is largely corroborated by the DFT calculations discussed above (Table 2) and *in situ* measurements of the paramagnetic susceptibilities by means of the Evans NMR method (Table S1, Figure S31 and Table 2). Experiment and theory are in excellent agreement for **3-Cr**, **3-Mn** and **3-Co**, whose magnetic moments (μ_eff_) and calculated spin multiplicities (2S + 1) suggest the presence of *n* = 4 (**3-Cr**), *n* = 5 (**3-Mn**) and *n* = 1 (**3-Co**) unpaired electrons per 3d transition-metal center, in consistency with Cr­(+II) and Mn­(+II) ions in their respective high-spin configurations and a Co­(+II) ion in its low-spin configuration.

The experimentally determined magnetic moment of **3-Fe** (μ_eff_ = 4.08 μ_B_) is lower than expected for the computationally derived spin configuration with *n*_comp_ = 4 unpaired electrons *per* Fe center but still in broad agreement (*n*_exp_ between 3 and 4). Theoretically, the optimized structure of the triplet spin state structure compares well with the experiment but is not computed to be the ground state (see Tables S6 and S11), which is a quintet. The analysis of the unpaired spin density in both indicates the presence of 3.7 unpaired electrons for the quintet and 2.4 in the triplet, so that the experimental magnetic measurement could correspond to a mixture of these two spin states (see Figure S33). Additionally, larger basis sets, 6–311G­(d,p) instead of 6–31G­(d,p), and an alternative functional with a smaller Hartree–Fock exchange contribution, PBE0 instead of B3PW91, were employed in order to rule out a possible overstabilization of the high-spin (quintet) configuration by the DFT method, especially for **3-Fe**. However, neither the basis-set quality nor a change in functional significantly affected the relative energy differences between the spin states or the A, B and C forms for the series of complexes **3-M** (Table S14).

Unfortunately, a comparison between the M···Cp’ distances in the monometallic precursor complexes **2-MX** and the bimetallic species **3-M** reveals no clear tendency toward higher or lower values. The transition from a low-spin to a high-spin configuration is generally accompanied by an increased M···Cp’ distance, and *vice versa*. However, the relatively small changes observed when going from **2-MX** to the corresponding **3-M** species (Table 2) may further support the absence of a spin state change with respect to the precursor.

## Conclusions

Five structurally related heterobimetallic complexes associating tantalum and 3d transition metals ranging from chromium to nickel were prepared *via* salt metathesis from monometallic precursors and their structural and electronic properties were investigated. The use of sterically demanding perhydrocarbyl ligands provides great flexibility in the choice of the 3d transition metal and allows to stabilize metal–metal pairs with widely differing numbers of valence electrons without significantly affecting the molecular structure. The relatively short metal–metal distances compared to other, literature-known Ta–M­(3*d*) species are most likely explained by the impact of the bridging alkylidene ligands rather than actual metal–metal interactions. The choice of the 3d transition metal used during the synthesis influences the hybridization states of the α-carbon atoms in the Ta coordination sphere, giving rise to a tris­(alkyl)-alkylidyne species if M = Mn and bis­(alkyl)-bis­(μ-alkylidene) species if M = Cr, Fe, Co and Ni. Density-functional theory calculations indeed suggest the existence of different tautomeric forms lying energetically close to each other, which result from the mutual exchange of α-hydrogen atoms between the neopentyl and neopentylidene or neopentylidyne ligands. Even though this tautomerism influences the complexes’ structural parameters, especially in proximity to the Ta center, it only has a minor impact on the electronic configurations of the 3d transition-metal ions: computational investigations and magnetic susceptibility measurements using the Evans method indicate the same spin multiplicities as in the corresponding monometallic precursors (except for M = Ni, in which case NMR spectroscopy is much more informative) and therefore corroborate the description of the heterobimetallic complexes as formal Ta­(+V)-M­(+II) species.

The salt-metathesis approach involving an alkali-stabilized alkyltantalate complex and a series of 3d transition-metal cyclopentadienyl halide dimers proves to be robust and versatile. Nonetheless, the formation of an unexpected anionic Ta–Mn halide species upon exchanging chloride for iodide ligands in the starting material **2-MnX** also demonstrates its limitations. Moreover, the lack of stability of part of the heterobimetallic series **3-M** even in noncoordinating solvents at ambient temperature as well as the paramagnetic properties of all compounds except **3-Ni**, which in turn shows the fastest decomposition, seriously hampers further reactivity studies. Future work will be directed at expanding the range of currently available perhydrocarbyl-stabilized heterobimetallic complexes to species that are more convenient as precursors for SOMC applications.

## Experimental Section

### General Considerations

Unless otherwise stated, all reactions were carried out under an argon atmosphere using a standard dual manifold Schlenk line setup. Materials and reactants sensitive to air and moisture were manipulated in an MBraun glovebox operating at H_2_O and O_2_ levels of <0.1 ppm. All glassware was dried at 100 °C for at least 24 h prior to use. Tetrahydrofuran, diethyl ether, toluene and *n*-pentane were purified by passage through a column of activated alumina, dried in the presence of sodium and benzophenone, vacuum-transferred to a storage flask and freeze–pump–thaw degassed. Deuterated solvents (THF-d_8_, C_6_D_6_) were dried over sodium and benzophenone, vacuum-transferred to a storage flask and freeze–pump–thaw degassed. Hexamethyldisiloxane was dried by refluxing and distilling in the presence of calcium hydride and freeze–pump–thaw degassed. Compounds **1**,^63^ **2-CrCl**,^57^ **2-MnCl**,^61^ **2-MnI**,^61^ [MnI_2_(thf)_3_],^83^ **2-FeI**,^58^ **2-CoI** ^59^ and **2-NiBr** ^60^ were prepared according to literature procedures. All other reagents were purchased from commercial sources and used without any further purification.

Caution! Extreme care should be taken both in the handling of the cryogen liquid nitrogen and its use in the Schlenk line trap to avoid the condensation of oxygen from air.

Caution! Alkali metals and ketyl radical solutions are highly reducing. They must not come into contact with air or oxidizing agents. Quenching should be carried out at low temperature under an argon atmosphere by the slow, sequential addition of t-BuOH, then i-PrOH, then EtOH and finally H_2_O.

Caution! The organometallic compounds described in this work are air-sensitive and potentially pyrophoric. Although the procedures reported here mitigate these risks by employing millimolar concentrations, particular care is required if scale-up is attempted.

Caution! All chemicals may present toxic or hazardous properties. Readers are strongly encouraged to consult the corresponding Material Safety Data Sheets (MSDS) for detailed information on safe handling, potential risks, and recommended protective measures.

### Characterization Methods

#### Infrared Spectroscopy

Solid samples were diluted in dry potassium bromide (KBr) inside a glovebox, sealed under argon in a DRIFTS cell equipped with KBr windows and analyzed on a Nicolet 670 FT-IR spectrometer.

#### UV–Visible Absorption Spectroscopy

Samples were dissolved in *n*-pentane, diluted (c = 40 μM) and transferred to a quartz cuvette equipped with a J. Young Teflon valve inside a glovebox. Absorption spectra were recorded on a PerkinElmer Lambda 1050 UV/vis/NIR spectrophotometer.

#### Elemental Analysis

Elemental analyses were performed under inert atmosphere at Mikroanalytisches Labor Pascher, Germany.

#### X-ray Structural Determination

Experimental details regarding single-crystal X-ray diffraction measurements are provided in the SI. CCDC 2420092–2420098 contain the supplementary crystallographic data for this publication. These data are provided free of charge by the Cambridge Crystallographic Data Center.

#### NMR Spectroscopy

Solution NMR spectra were recorded on a Bruker AV-300 spectrometer. ^1^H and ^13^C chemical shifts are reported relative to an external tetramethylsilane standard at 0.00 ppm and were manually referenced to the residual solvent signal in Mestre Nova.

#### Computational Methods

Experimental details regarding density functional theory studies are provided in the SI.

#### Electron Paramagnetic Resonance

Experimental details regarding EPR measurements and simulation are provided in the SI.

### Synthetic Procedures

#### Synthesis of [Ta­(CH_2_tBu)_2_(μ-CHtBu)_2_CrCp’] (**3-Cr**)

An orange solution of [Li­(thf)_2_]­[Ta­(C*t*Bu)­(CH_2_ *t*Bu)_3_] (**1**, 500 mg, 0.814 mmol) in 3 mL *n*-pentane prepared at ambient temperature was added dropwise to a dark blue solution of [Cp’Cr­(μ-Cl)]_2_ (**2-Cr**, 261 mg, 0.407 mmol) in 5 mL *n*-pentane which had been cooled to −40 °C. The dark brown to black reaction mixture was stirred at ambient temperature for 24 h, leading to the formation of a gray precipitate. The solvents were removed under reduced pressure. After thorough drying under vacuum, the residual solids were taken up in 1.5 mL *n*-pentane and filtered through a Pasteur pipet equipped with a pad of glass microfiber filter to remove the salt (lithium chloride). The black filtrate was stored at −40 °C for 24 h to obtain **3-Cr** as black, block-shaped crystals (540 mg, 89% yield). ^1^H NMR (300 MHz, C_6_D_6_, 296 K): δ/ppm = 26.66, 14.75, 3.26. Elemental analysis (%). Calculated for **3-Cr** (C_37_H_71_CrTa): C 59.34, H 9.55; found: C 59.44, H 9.51.

#### Synthesis of [Ta­(CH_2_tBu)_3_(μ-CtBu)­MnCp’] (**3-Mn**)

An orange solution of [Li­(thf)_2_]­[Ta­(C*t*Bu)­(CH_2_ *t*Bu)_3_] (**1**, 200 mg, 0.325 mmol) in 3 mL *n*-pentane prepared at ambient temperature was added dropwise to a pale green solution of [Cp’Mn­(thf)­(μ-Cl)]_2_ (**2-MnCl**, 134 mg, 0.162 mmol) in 5 mL *n*-pentane which had been cooled to −40 °C. The red reaction mixture was stirred at ambient temperature for one hour, leading to the formation of a gray precipitate. The solvents were removed under reduced pressure. After thorough drying under vacuum, the residual solids were taken up in 2.0 mL *n*-pentane and filtered through a Pasteur pipet equipped with a pad of glass microfiber filter to remove the salt (lithium chloride). The red filtrate was stored at −40 °C for 24 h to obtain **3-Mn** as a red, microcrystalline powder (176 mg, 72% yield). Small and very fragile red crystals for X-ray structural analysis were obtained by recrystallizing the dried powder from a slightly larger volume of hexamethyldisiloxane (3.0 mL) at −40 °C for 48 h (98 mg, 40% yield). ^1^H NMR (300 MHz, C_6_D_6_, 296 K): δ/ppm = 32.12, 5.96. Elemental analysis (%). Calculated for **3-Mn** (C_37_H_71_MnTa): C 59.11, H 9.52; found: C 58.82, H 9.62.

#### Synthesis of [Li­(thf)_4_]­[Ta­(CH_2_tBu)_3_(μ-CtBu)­MnI_2_] (**3-Mn′**)

An orange solution of [Li­(thf)_2_]­[Ta­(C*t*Bu)­(CH_2_ *t*Bu)_3_] (**1**, 100 mg, 0.162 mmol) in 3 mL *n*-pentane prepared at ambient temperature was added dropwise to a pale turquoise solution of [Cp’Mn­(thf)­(μ-I)]_2_ (**2-MnI**, 163 mg, 0.162 mmol) in 5 mL *n*-pentane which had been cooled to −40 °C. The reaction mixture was stirred at ambient temperature for one hour, leading to the formation of a brown oil suspended in a deep orange solution. The solvents were removed under reduced pressure. After thorough drying under vacuum, the residual solids were taken up in 2.5 mL *n*-pentane and filtered through a Pasteur pipet equipped with a pad of glass microfiber filter to remove the salt (lithium iodide). The orange filtrate was stored at −40 °C for 24 h to obtain **3-Mn′** as an orange-red, microcrystalline powder (144 mg, 83% yield). Small and very fragile red crystals for X-ray structural analysis were obtained by recrystallizing the dried powder from a slightly larger volume of hexamethyldisiloxane (3.5 mL) at −40 °C for 48 h (81 mg, 47% yield). ^1^H NMR (300 MHz, C_6_D_6_, 296 K): δ/ppm = 5.78, 3.68, 2.01, 0.12 (HMDSO).

#### Synthesis of [Ta­(CH_2_tBu)_3_(μ-CtBu)­MnI_2_Li­(thf)_2_] (**3-Mn″**)

An orange of solution of [Li­(thf)_2_]­[Ta­(C*t*Bu)­(CH_2_ *t*Bu)_3_] (**1**, 100 mg, 0.163 mmol) in 2 mL *n*-pentane was added dropwise to a pale rose suspension of [MnI_2_(thf)_3_] (94 mg, 0.179 mmol) in 3 mL toluene prepared at ambient temperature. The reaction mixture was stirred for two hours and gradually transformed into a cherry-red solution. The solvents were removed under reduced pressure. After thorough drying under vacuum, the residual solids were taken up in 2.0 mL *n*-pentane and filtered through a Pasteur pipet equipped with a pad of glass microfiber filter. The filtrate was stored at −40 °C for 24 h to obtain **3-Mn″** as red crystals (101 mg, 67%). ^1^H NMR (300 MHz, C_6_D_6_, 296 K): δ/ppm = 5.94, 2.18, traces of pentane (1.23, 0.88). Elemental analysis (%). Calculated for **3-Mn″** (C_28_H_58_O_2_LiI_2_MnTa): C 36.42, H 6.33; found: C 36.09, H 6.30.

#### Synthesis of [Ta­(CH_2_tBu)_2_(μ-CHtBu)_2_FeCp’] (**3-Fe**)

An orange solution of [Li­(thf)_2_]­[Ta­(C*t*Bu)­(CH_2_ *t*Bu)_3_] (**1**, 200 mg, 0.325 mmol) in 3 mL *n*-pentane prepared at ambient temperature was added dropwise to a dark brown solution of [Cp’Fe­(μ-I)]_2_ (**2-Fe**, 135 mg, 0.163 mmol) in 5 mL *n*-pentane which had been cooled to −40 °C. The dark brown to black reaction mixture was stirred at ambient temperature for 30 min, leading to the formation of a gray precipitate. The solvents were removed under reduced pressure. After thorough drying under vacuum, the residual solids were taken up in 5 mL *n*-pentane and filtered through a Pasteur pipet equipped with a pad of glass microfiber filter to remove the salt (lithium iodide). The filtrate was taken to dryness again, the remaining black solid dissolved in a minimum amount of hexamethyldisiloxane (1.0 mL) and the resulting solution filtered in the way described above. The black filtrate was stored at −40 °C for 24 h to obtain **3-Fe** as black, block-shaped crystals (210 mg, 77% yield). ^1^H NMR (300 MHz, C_6_D_6_, 296 K): δ/ppm = 62.28, 4.38, 0.10 (HMDSO), 1.12, 1.97, −21.36, −35.90, −47.45. Elemental analysis (%). Calculated for **3-Fe** · 1/2 HMDSO (C_40_H_80_O_0.5_SiFeTa): C 57.61, H 9.67; found: C 56.26, H 9.19. *Despite multiple attempts and careful shipment of the sample under argon, elemental analysis consistently showed slightly low carbon content, which we attribute to the thermal instability of the complex leading to the release of volatile neopentane.*

#### Synthesis of [Ta­(CH_2_tBu)_2_(μ-CHtBu)_2_CoCp’] (**3-Co**)

An orange solution of [Li­(thf)_2_]­[Ta­(C*t*Bu)­(CH_2_ *t*Bu)_3_] (**1**, 300 mg, 0.488 mmol) in 3 mL *n*-pentane prepared at ambient temperature was added dropwise to a black solution of [Cp’Co­(μ-I)]_2_ (**2-Co**, 205 mg, 0.244 mmol) in 5 mL *n*-pentane which had been cooled to −40 °C. The dark brown to black reaction mixture was stirred at ambient temperature for three hours, leading to the formation of a gray precipitate. The solvents were removed under reduced pressure. After thorough drying under vacuum, the residual solids were taken up in 5 mL *n*-pentane and filtered through a Pasteur pipet equipped with a pad of glass microfiber filter to remove the salt (lithium iodide). The filtrate was taken to dryness again, the remaining black solid dissolved in a minimum amount of hexamethyldisiloxane (1.0 mL) and the resulting solution filtered in the way described above. The black filtrate was stored at −40 °C for 24 h to obtain **3-Co** as black, block-shaped crystals (215 mg, 53% yield). ^1^H NMR (300 MHz, C_6_D_6_, 296 K): δ/ppm = 14.12, 3.02, 1.77, 0.59, 0.12 (HMDSO). Elemental analysis (%). Calculated for **3-Co** · 1/2 HMDSO (C_40_H_80_O_0.5_SiCoTa): C 57.40, H 9.63; found: C 57.90, H 9.36.

#### Synthesis of [Ta­(CH_2_tBu)_2_(μ-CHtBu)_2_NiCp’] (**3-Ni**)

An orange solution of [Li­(thf)_2_]­[Ta­(C*t*Bu)­(CH_2_ *t*Bu)_3_] (**1**, 500 mg, 0.814 mmol) in 3 mL *n*-pentane prepared at ambient temperature was added dropwise to a dark red solution of [Cp’Ni­(μ-Br)]_2_ (**2-Ni**, 303 mg, 0.407 mmol) in 5 mL *n*-pentane which had been cooled to −40 °C. The dark brown to black reaction mixture was stirred at ambient temperature for 10 min, leading to the formation of a gray precipitate. The solvents were removed under reduced pressure. After thorough drying under vacuum, the residual solids were taken up in 5 mL *n*-pentane and filtered through a Pasteur pipet equipped with a pad of glass microfiber filter to remove the salt (lithium bromide). The filtrate was taken to dryness again, the remaining black solid dissolved in a minimum amount of hexamethyldisiloxane (0.8 mL) and the resulting solution filtered in the way described above. The black filtrate was stored at −40 °C for 48 h to obtain **3-Ni** as black, block-shaped crystals (200 mg, 29% yield). ^1^H NMR (300 MHz, C_6_D_6_, 296 K): δ/ppm = 5.83 (s, 2 H, *t*Bu_3_C_5_ *H*_2_), 4.15 (s, 2 H, Ta–C*Ht*Bu–Ni), 1.68 (s, 9 H, *t*Bu), 1.35 (superposition of two s, 36 H, 1,2-tBu_Cp’_ and Ta–CHt*Bu*–Ni), 1.25 (s, 9 H, *t*Bu), 1.16 (s, 9 H, *t*Bu), 0.61 (s, 2 H, Ta–C*H*_2_ *t*Bu), 0.50 (s, 2 H, Ta–C*H*_2_ *t*Bu), 0.12 (s, 9 H, (*Me*_3_Si)_2_O). ^13^C­{^1^H} NMR (126 MHz, C_6_D_6_, 298 K): δ/ppm = 187.47 (Ta–*C*H*t*Bu–Ni), 146.06 (Ta–*C*H_2_ *t*Bu), 125.65, 121.02, 104.20 (Ta–*C*H_2_ *t*Bu), 82.89 (*C*_Cp’_^3*,5*^), 43.54, 40.57, 36.72, 35.16, 34.92, 34.62, 34.04, 33.75, 32.69, 2.10 ((*Me*_3_Si)_2_O). Elemental analysis (%). Calculated for **3-Ni** · 1/2 HMDSO (C_40_H_80_O_0.5_SiNiTa): C 57.41, H 9.63; found: C 57.56, H 9.21.

## Supplementary Material





## Notes

NMR, IR, EPR and UV–vis spectroscopy, X-ray crystallography and computational data (PDF)
DFT Structures (XYZ)
