# Supplementary material for: Probing Structural Diversity in a Series of Perhydrocarbyl Heterobimetallic Complexes Associating Tantalum and 3d (Cr, Mn, Fe, Co, Ni) Transition Metals
Source: Inorg Chem. 2025 Oct 17;64(43):21353–67. doi: 10.1021/acs.inorgchem.5c02380 (PMC12598868; doi:10.1021/acs.inorgchem.5c02380)
Supplement: Supplementary file 1 [file ic5c02380_si_001.pdf]

Supporting information

Probing structural diversity in a series of perhydrocarbyl heterobimetallic complexes associating tantalum and 3*d* (Cr, Mn, Fe, Co, Ni) transition metals

Till Neumann[a], Iker Del Rosal[b], Laurent Maron[b], Erwann Jeanneau[c], Vincent Maurel[d], Serge Gambarelli[d], Jean-Marie Mouesca[d], Chloé Thieuleux[a], and Clément Camp*[,a]

[a] Laboratory of Catalysis, Polymerization, Processes and Materials (CP2M, UMR 5128), CNRS, Universite Claude Bernard Lyon 1, CPE Lyon, Institut de Chimie de Lyon, 3 rue Victor Grignard, 69616 Villeurbanne, France

[b] LPCNO (UMR 5125), CNRS & INSA, Université Paul Sabatier, 135 Avenue de Rangueil, 31077 Toulouse, France

[c] Centre de Diffractométrie Henri Longchambon, Universite Claude Bernard Lyon 1, 5 Rue de la Doua, 69100 Villeurbanne, France

[d] Université Grenoble Alpes, CEA, CNRS, INAC, SyMMES, F-38000 Grenoble, France

clement.camp@univ-lyon1.fr

Contents

NMR-spectroscopic characterization data for compounds **3-M** (M = Cr, Mn, Fe, Co, Ni), **3-Mn'** and **3-Mn''** 2

UV-visible absorption spectra for compounds **3-M** (M = Cr, Mn, Fe, Co, Ni)........ 12

Diffuse reflectance infrared Fourier-transform spectroscopy (DRIFTS) data for compounds **3-M** (M = Cr, Mn, Fe, Co, Ni)........ 15

Evans magnetometry for paramagnetic compounds **3-M** (M = Cr, Mn, Fe, Co)........ 18

Crystallographic data for compounds **3-M** (M = Cr, Mn, Fe, Co, Ni), **3-Mn'** and **3-Mn''**..... 20

EPR spectroscopy........ 22

Computational details........ 26

References 40

NMR-spectroscopic characterization data for compounds **3-M** (M = Cr, Mn, Fe, Co, Ni), **3-Mn'** and **3-Mn''**

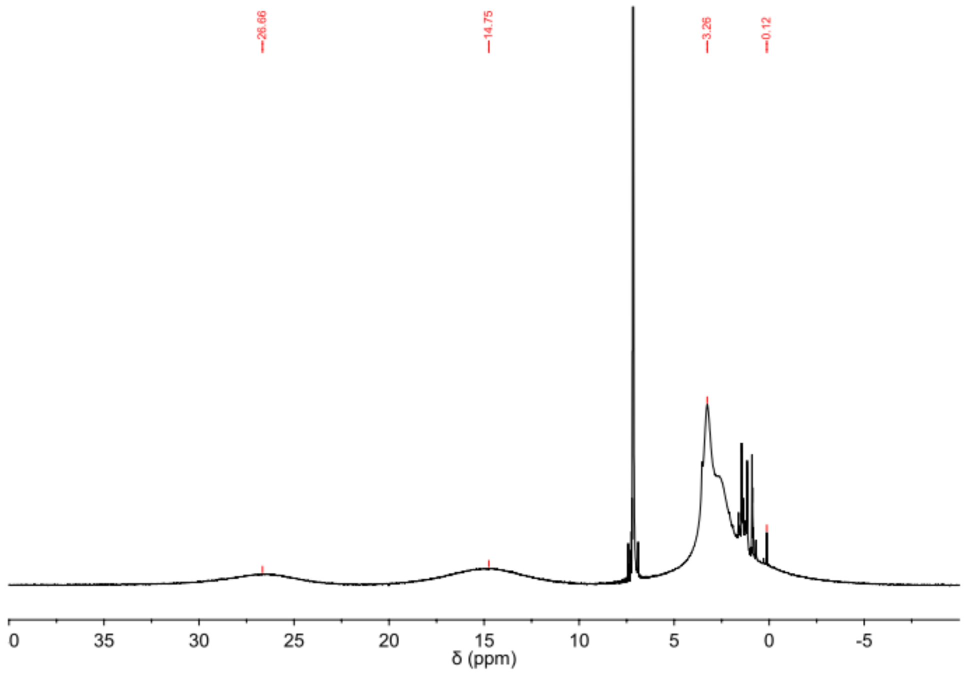

Figure S1. ^{1}H NMR spectrum (300 MHz, C_6D_6, 296 K) for compound **3-Cr**. Sharp resonances between 0.5 and 2.0 ppm due to trace amounts of **1** even in the recrystallized product.

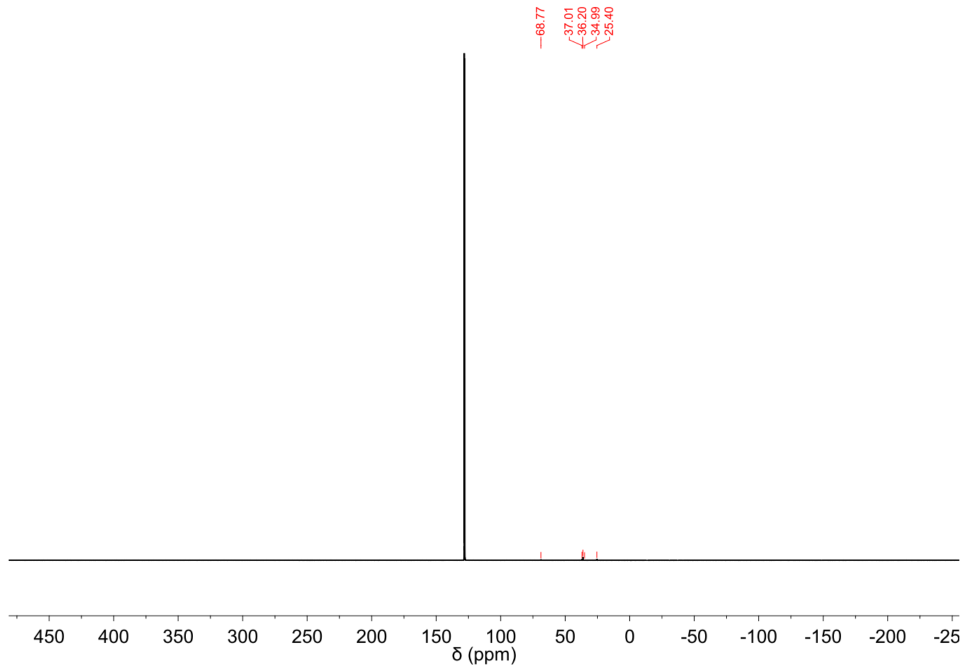

Figure S2. $^{13}C\{^{1}H\}$ NMR spectrum (126 MHz, C_6D_6, 293 K) for compound **3-Cr**. Sharp resonances due to trace amounts of **1** even in the recrystallized product.

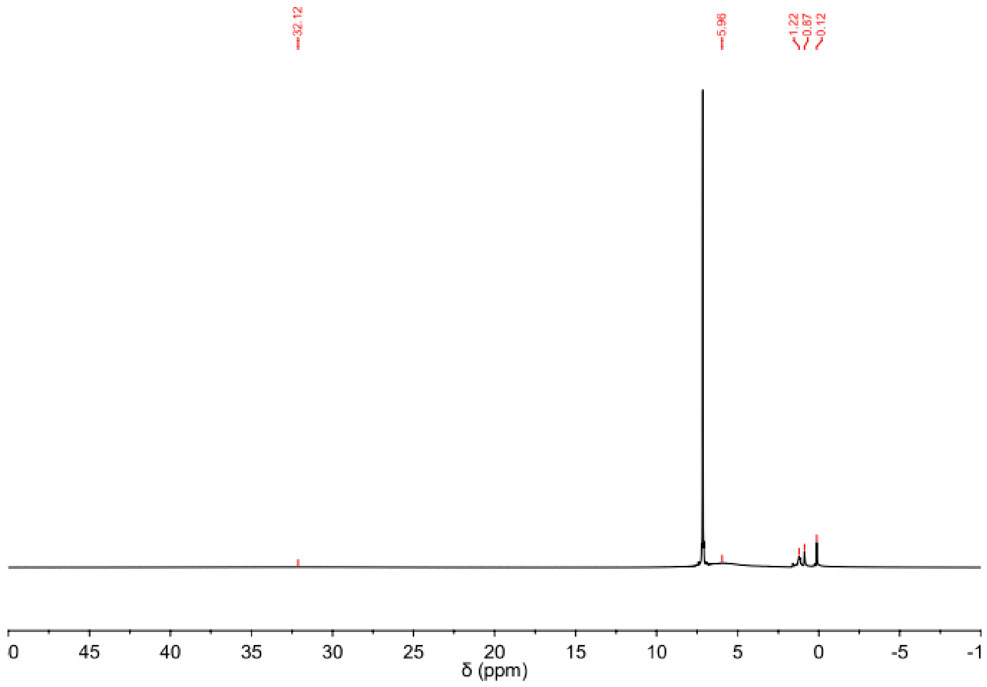

Figure S3. ^{1}H NMR spectrum (300 MHz, C_6D_6, 295 K) for compound **3-Mn**. Diamagnetic resonances are attributed to trace amounts of pentane (1.22, 0.87 ppm) and HMDSO (0.12 ppm).

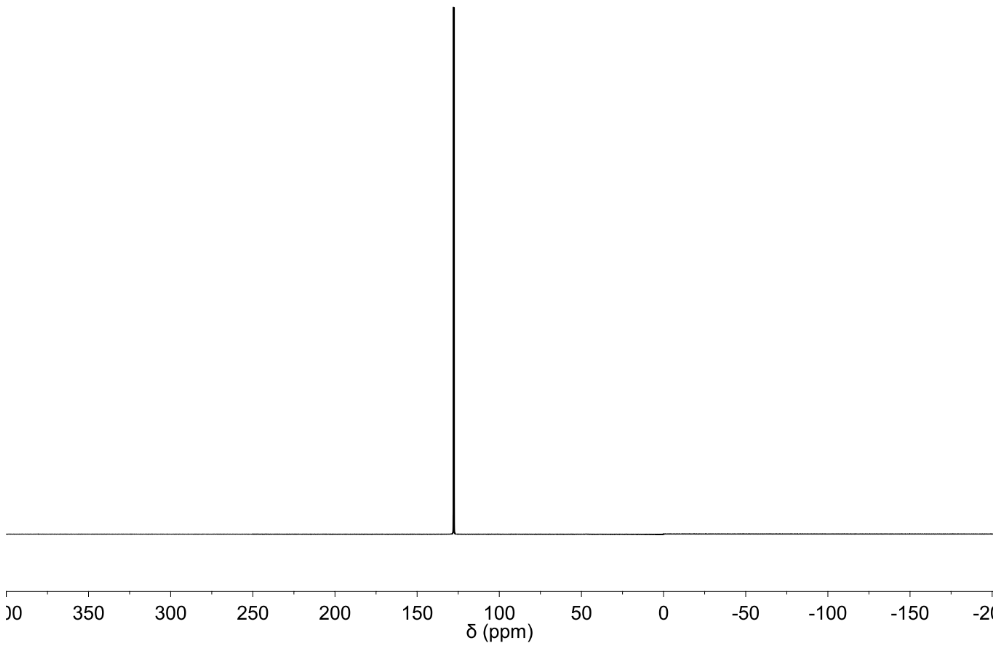

Figure S4. $^{13}C\{^{1}H\}$ NMR spectrum (126 MHz, C_6D_6, 293 K) for compound **3-Mn.**

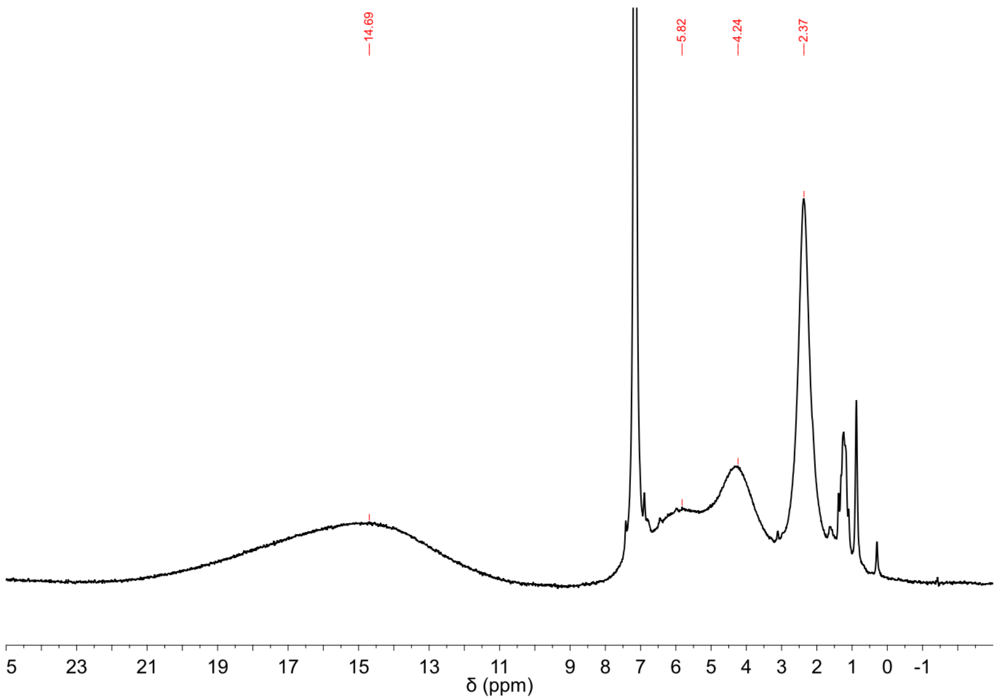

Figure S5. ¹H NMR spectrum (300 MHz, C_6D_6, 294 K) of the reaction between **1** and **2-MnI**. The broad resonances are attributed to MnCp’$_2$ (14.69 ppm) and **3-Mn’** (5.82, 4.24, 2.37 ppm), the diamagnetic resonances at 0.88 and 1.24 ppm to trace amounts of pentane co-crystallizing with **2-MnI**.

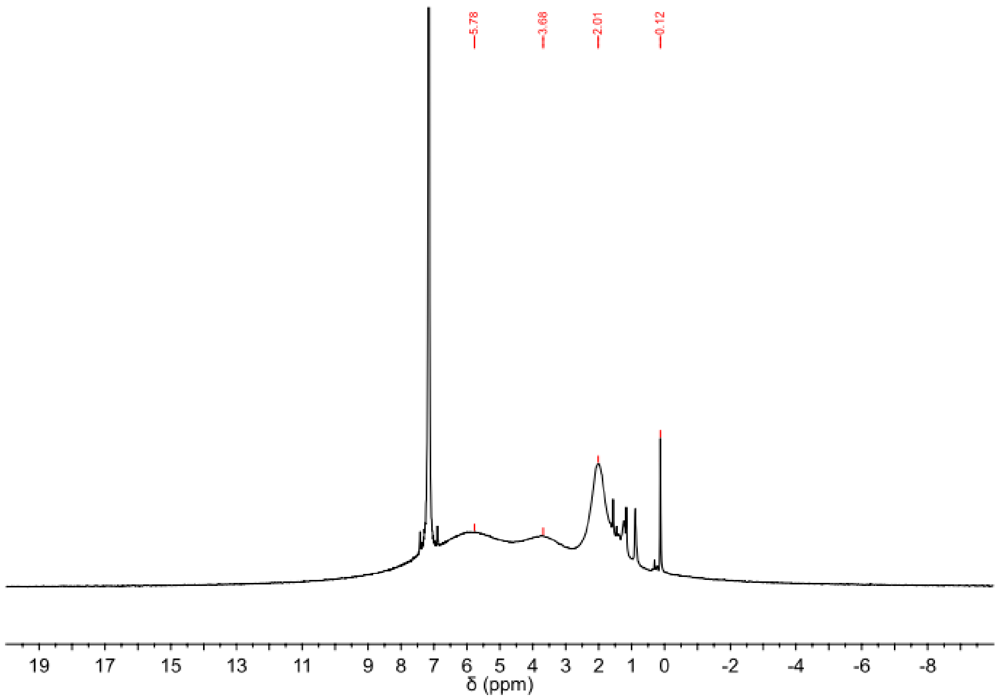

Figure S6. ¹H NMR spectrum (300 MHz, C_6D_6, 296 K) for compound **3-Mn’.**

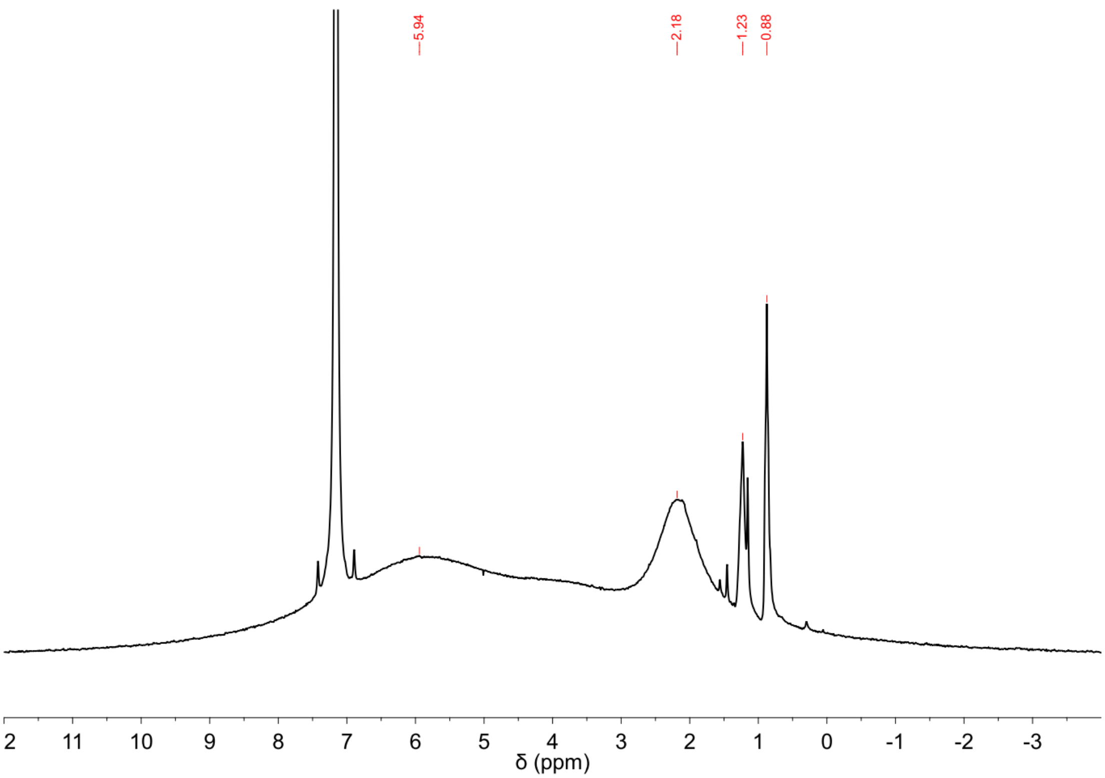

Figure S7. ^{1}H NMR spectrum (300 MHz, C_6D_6, 295 K) for compound **3-Mn”.**

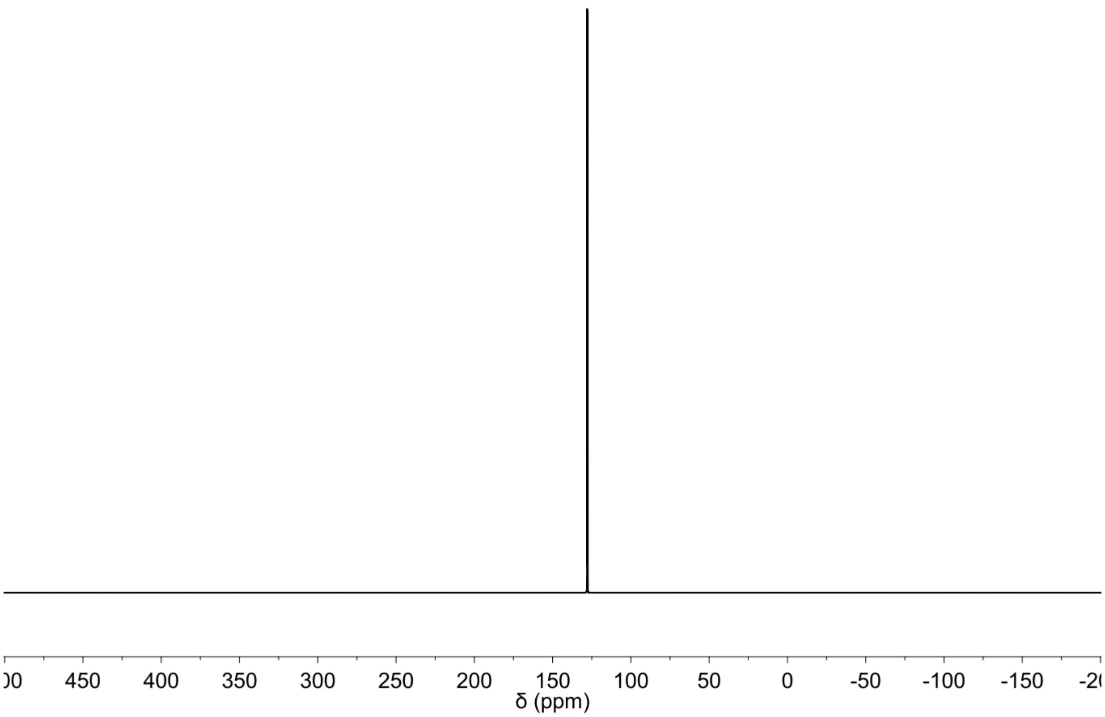

Figure S8. $^{13}C\{^{1}H\}$ NMR spectrum (126 MHz, C_6D_6, 293 K) for compound **3-Mn”.**

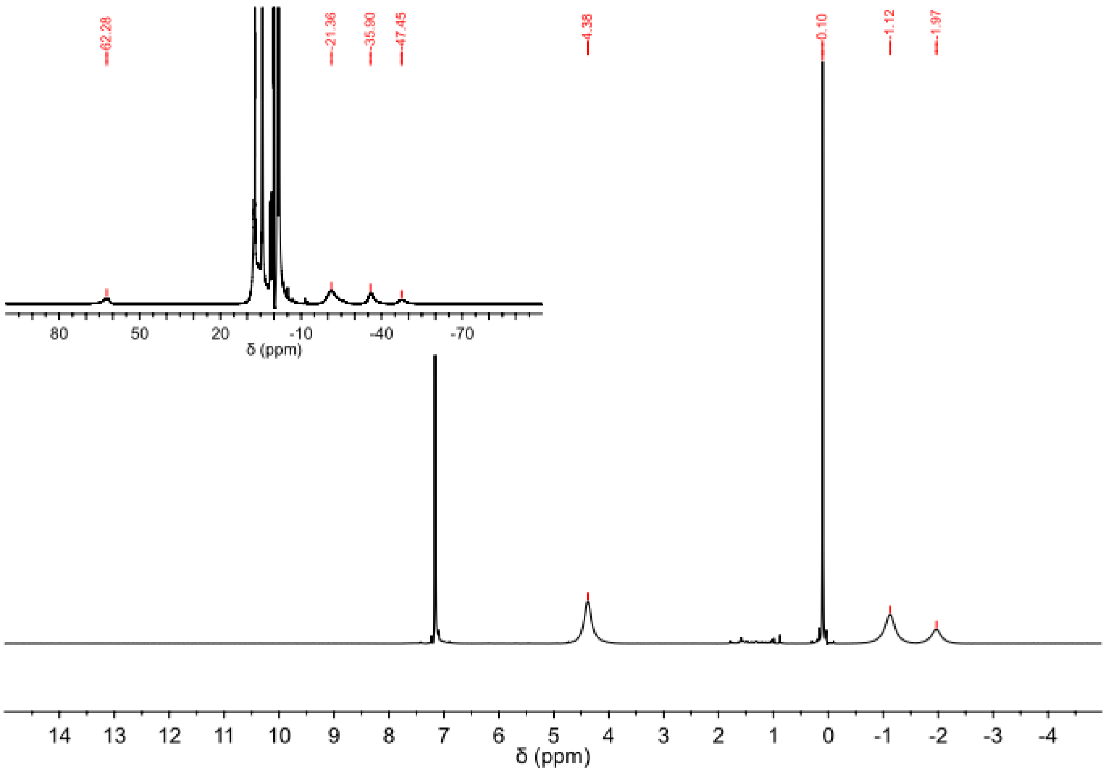

Figure S9. ^{1}H NMR spectrum (300 MHz, C_6D_6, 292 K) for compound **3-Fe.**

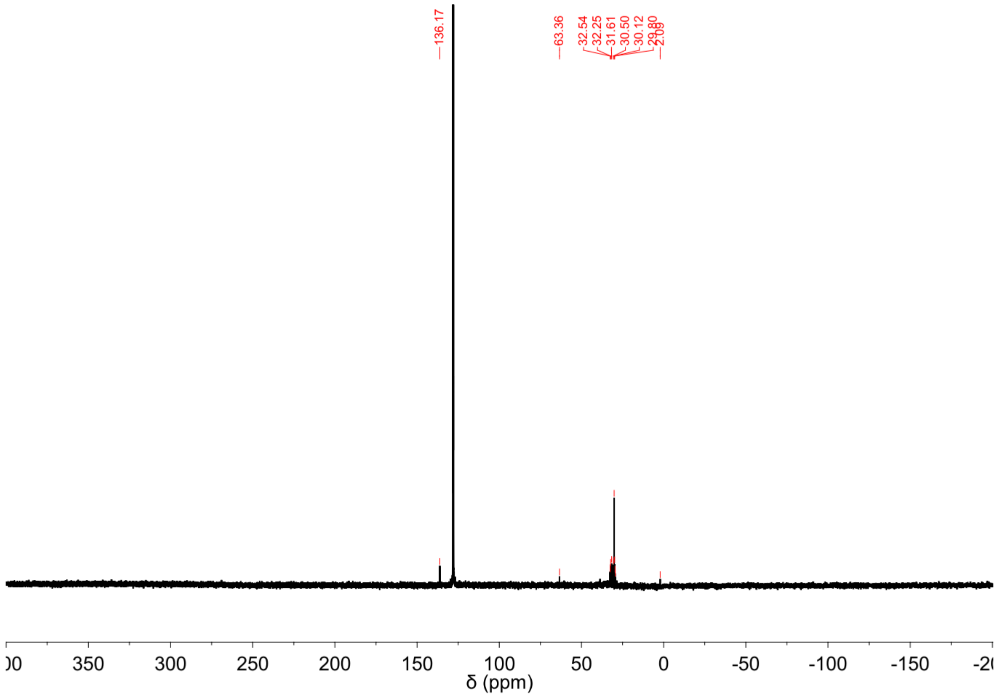

Figure S10. $^{13}C\{^{1}H\}$ NMR spectrum (126 MHz, C_6D_6, 293 K) for compound **3-Fe**. Sharp resonances grow during acquisition and are attributed to the diamagnetic products of the fast decomposition of **3-Fe** in solution. Resonance at 2.09 ppm due to HMDSO from purification.

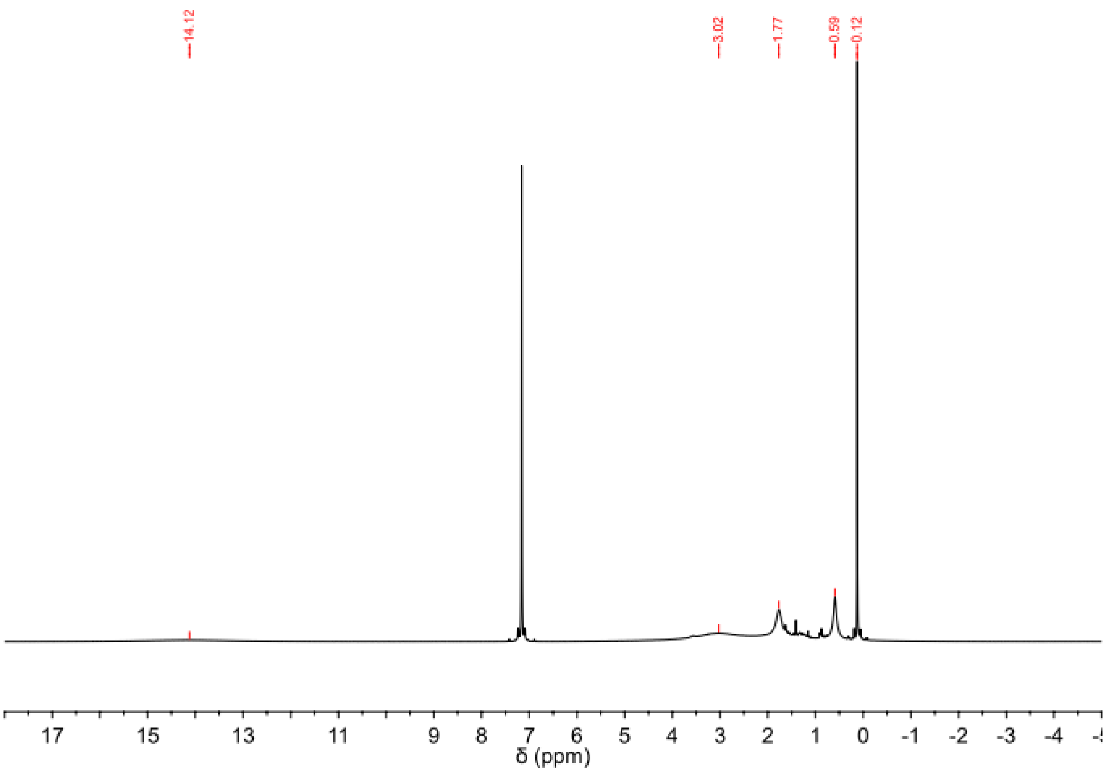

Figure S11. ^{1}H NMR spectrum (300 MHz, C_6D_6, 292 K) for compound **3-Co.**

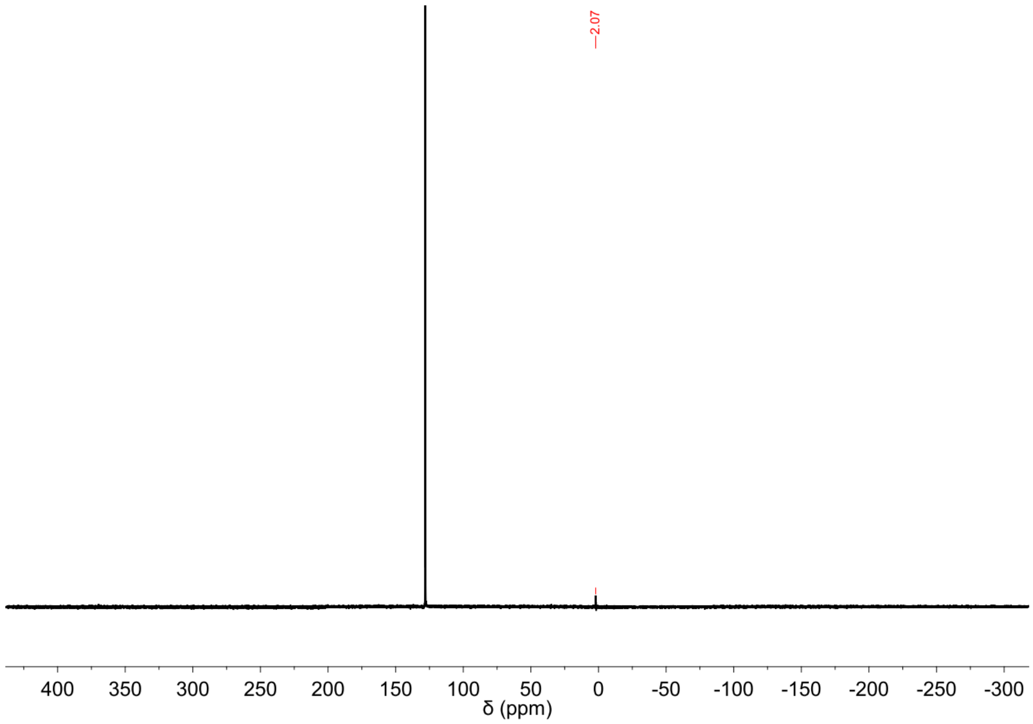

Figure S12. $^{13}C\{^{1}H\}$ NMR spectrum (126 MHz, C_6D_6, 293 K) for compound **3-Co**. Resonance at 2.07 ppm due to HMDSO from purification.

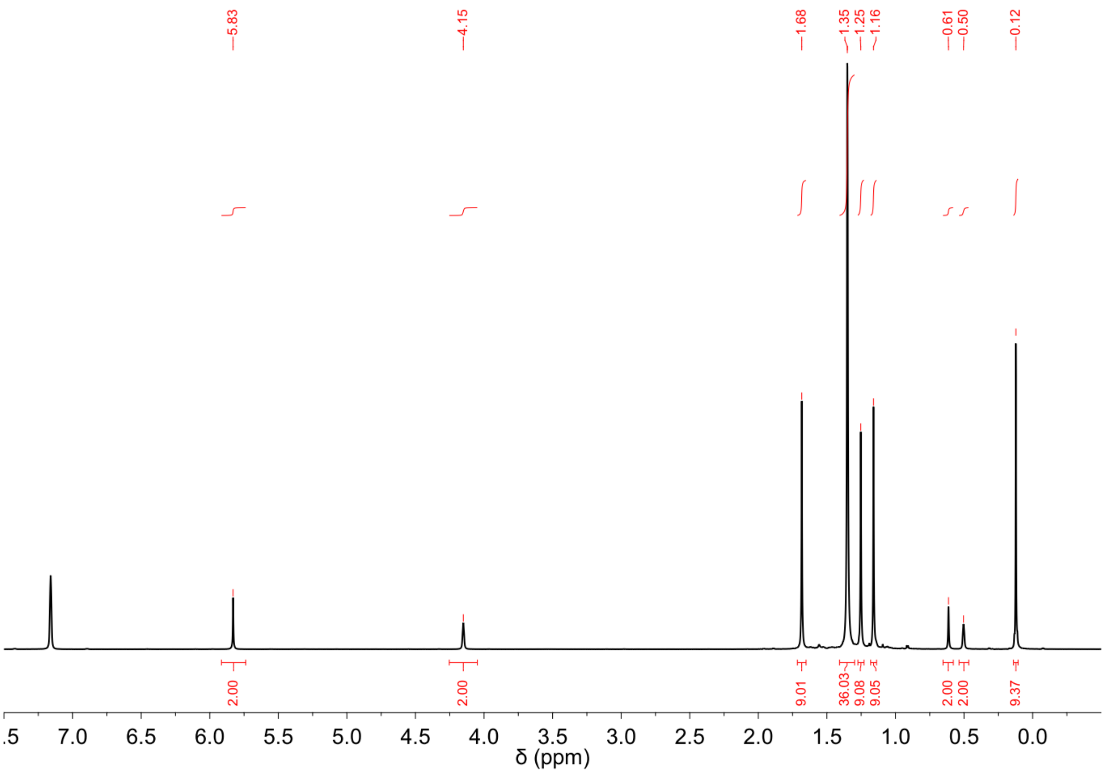

Figure S13. ^{1}H NMR spectrum (300 MHz, C_6D_6, 296 K) for compound **3-Ni.**

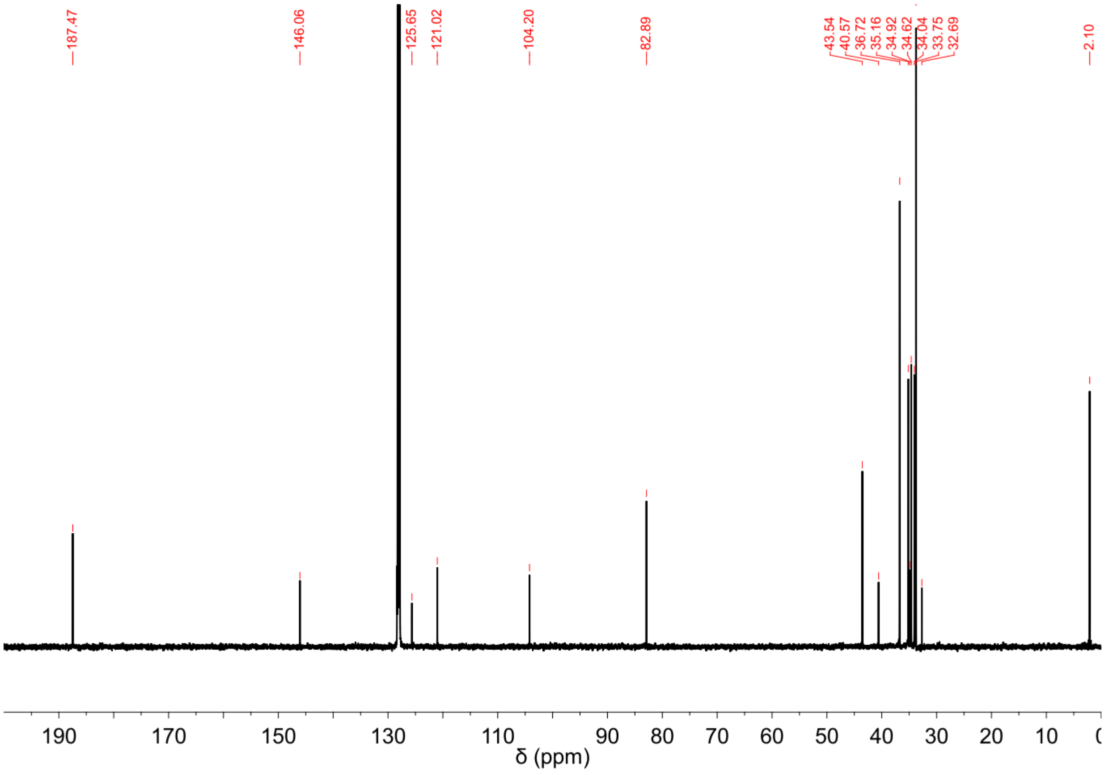

Figure S14. $^{13}C\{^{1}H\}$ NMR spectrum (126 MHz, C_6D_6, 298 K) for compound **3-Ni.**

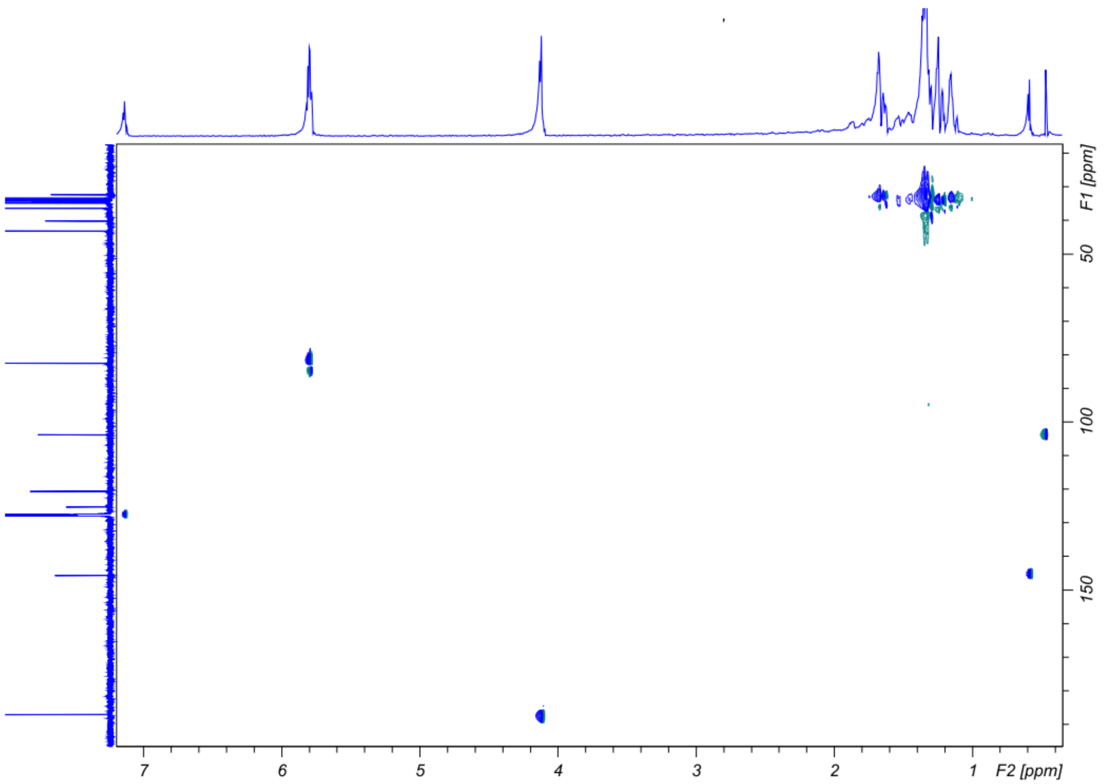

Figure S15. ^{1}H-^{13}C HSQC spectrum (500 MHz, C_6D_6, 298 K) for compound **3-Ni.**

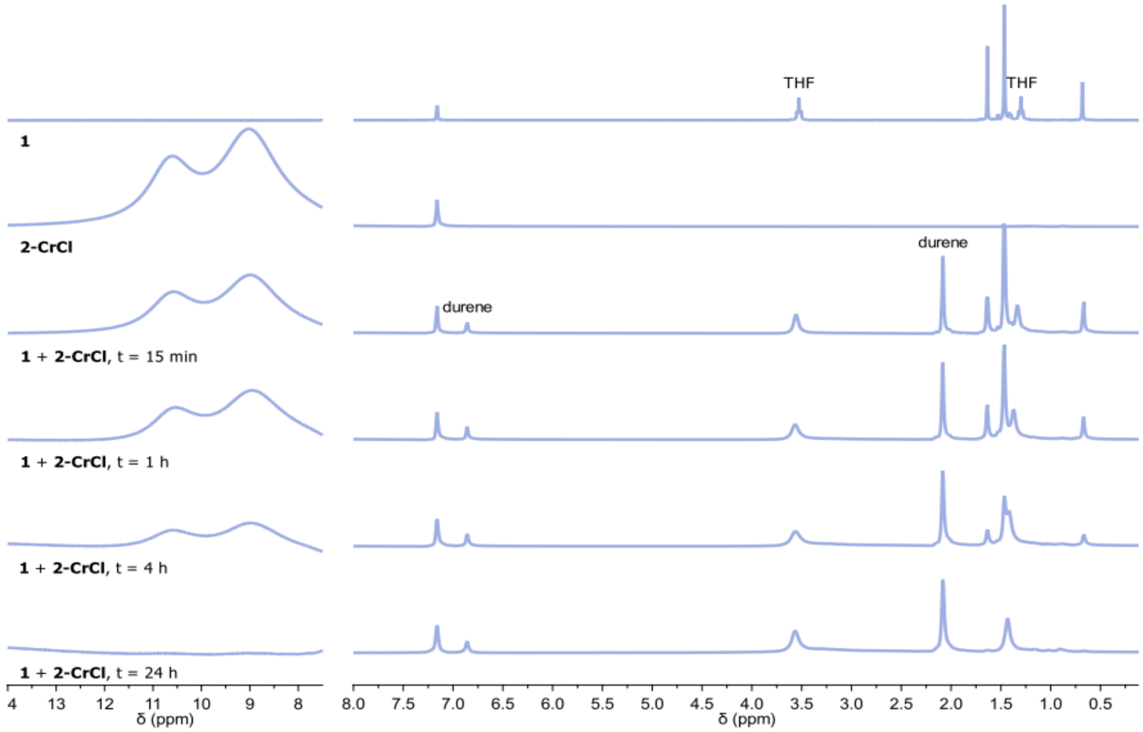

Figure S16. ^{1}H NMR monitoring (300 MHz, 298 K) of the reaction between **1** and **2-CrCl** in a C_6D_6 solution in the presence of 1,2,4,5-tetramethylbenzene (durene) as internal standard.

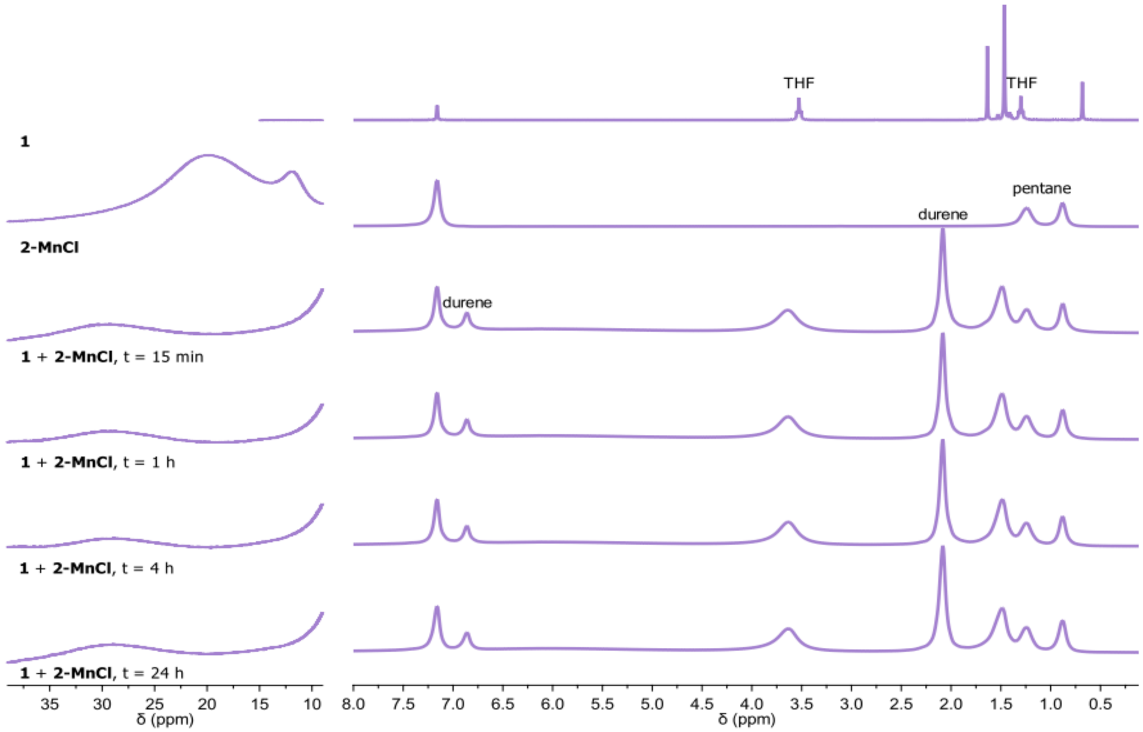

Figure S17. ^{1}H NMR monitoring (300 MHz, 298 K) of the reaction between **1** and **2-MnCl** (crystalline starting material containing 0.5 equiv. *n*-pentane) in a C_6D_6 solution in the presence of 1,2,4,5-tetramethylbenzene (durene) as internal standard.

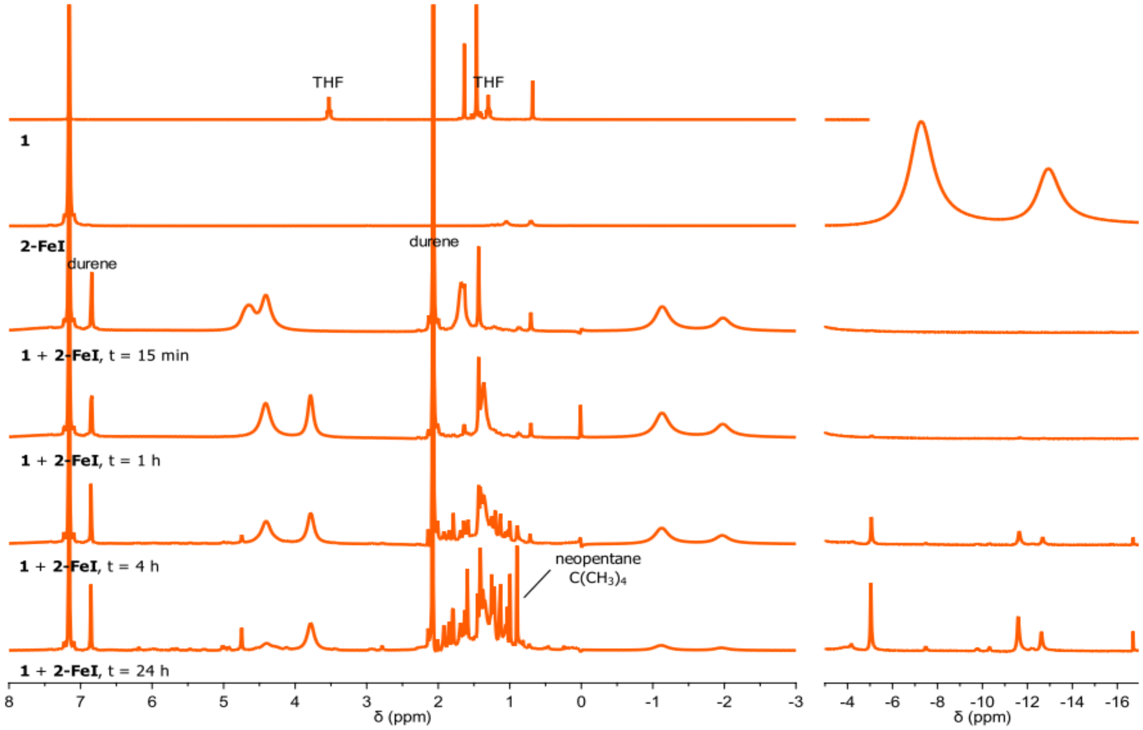

Figure S18. ^{1}H NMR monitoring (300 MHz, 298 K) of the reaction between **1** and **2-FeI** in a C_6D_6 solution in the presence of 1,2,4,5-tetramethylbenzene (durene) as internal standard.

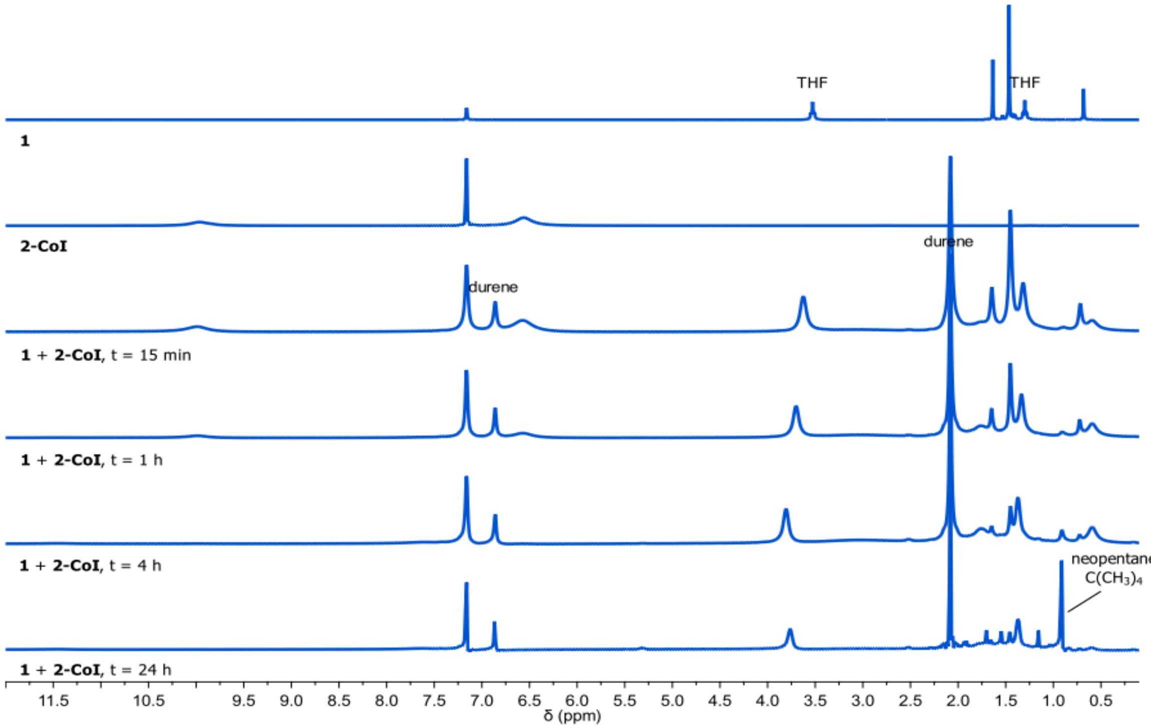

Figure S19. ^{1}H NMR monitoring (300 MHz, 298 K) of the reaction between **1** and **2-CoI** in a C_6D_6 solution in the presence of 1,2,4,5-tetramethylbenzene (durene) as internal standard.

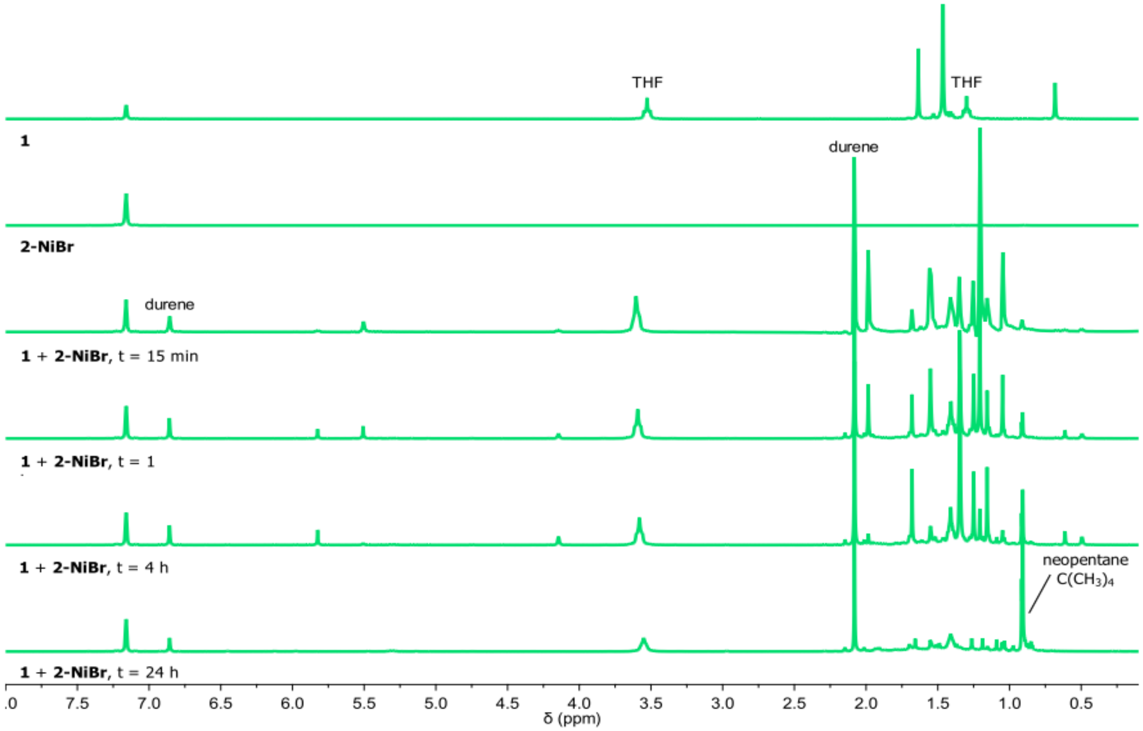

Figure S20. ^{1}H NMR monitoring (300 MHz, 298 K) of the reaction between **1** and **2-NiBr** in a C_6D_6 solution in the presence of 1,2,4,5-tetramethylbenzene (durene) as internal standard.

UV-visible absorption spectra for compounds **3-M** (M = Cr, Mn, Fe, Co, Ni)

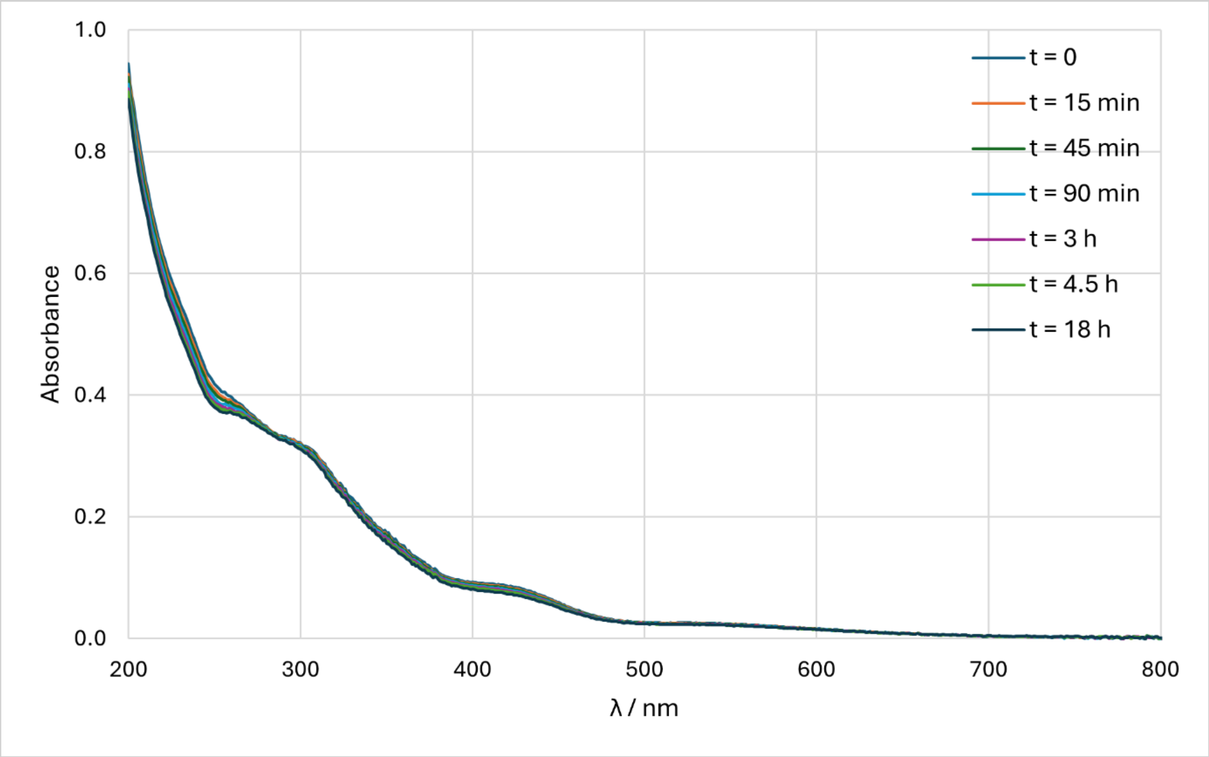

Figure S21. UV-visible spectra for compound **3-Cr** (c = 40 μM in pentane, 298 K).

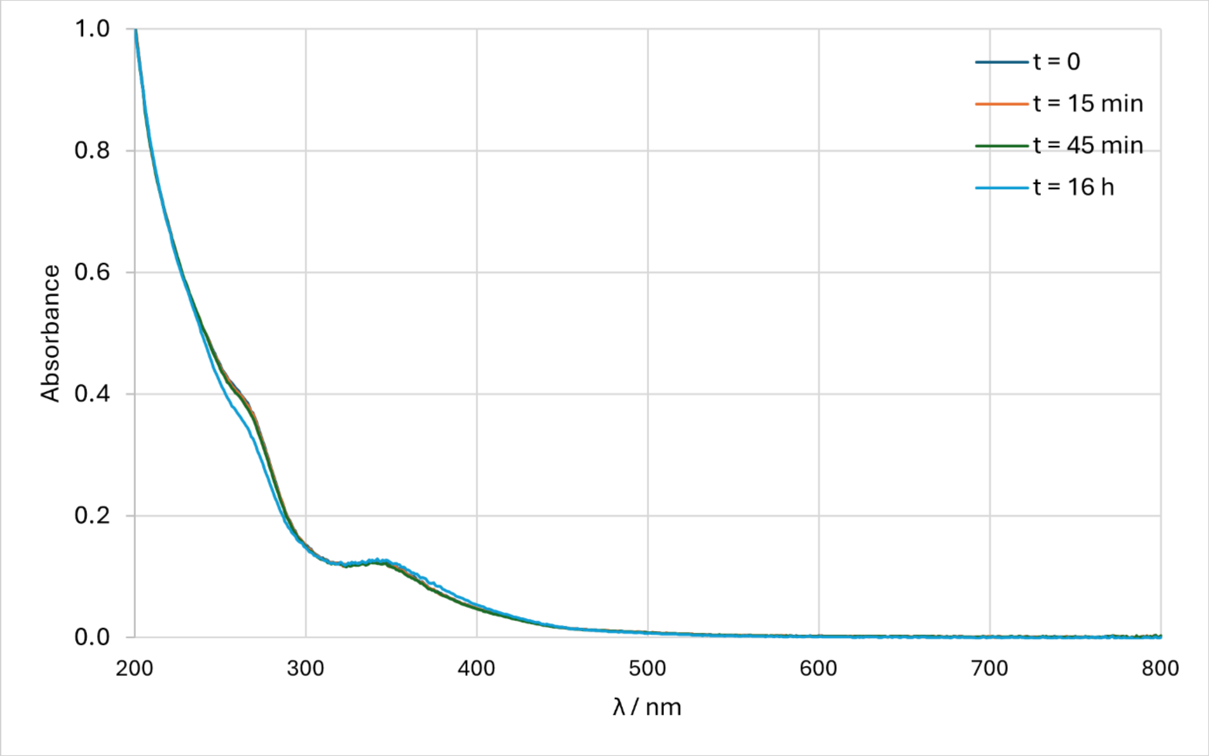

Figure S22. UV-visible spectra for compound **3-Mn** (c = 40 μM in pentane, 298 K).

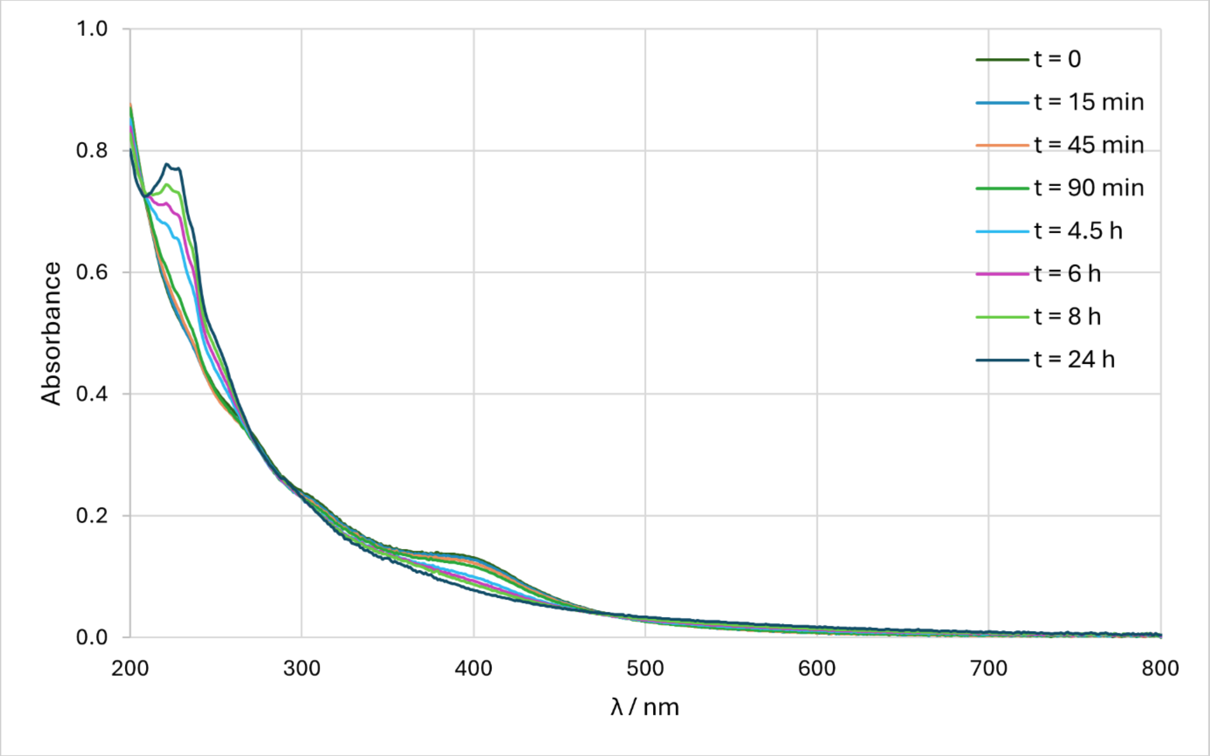

Figure S23. UV-visible spectra for compound **3-Fe** (c = 40 μM in pentane, 298 K).

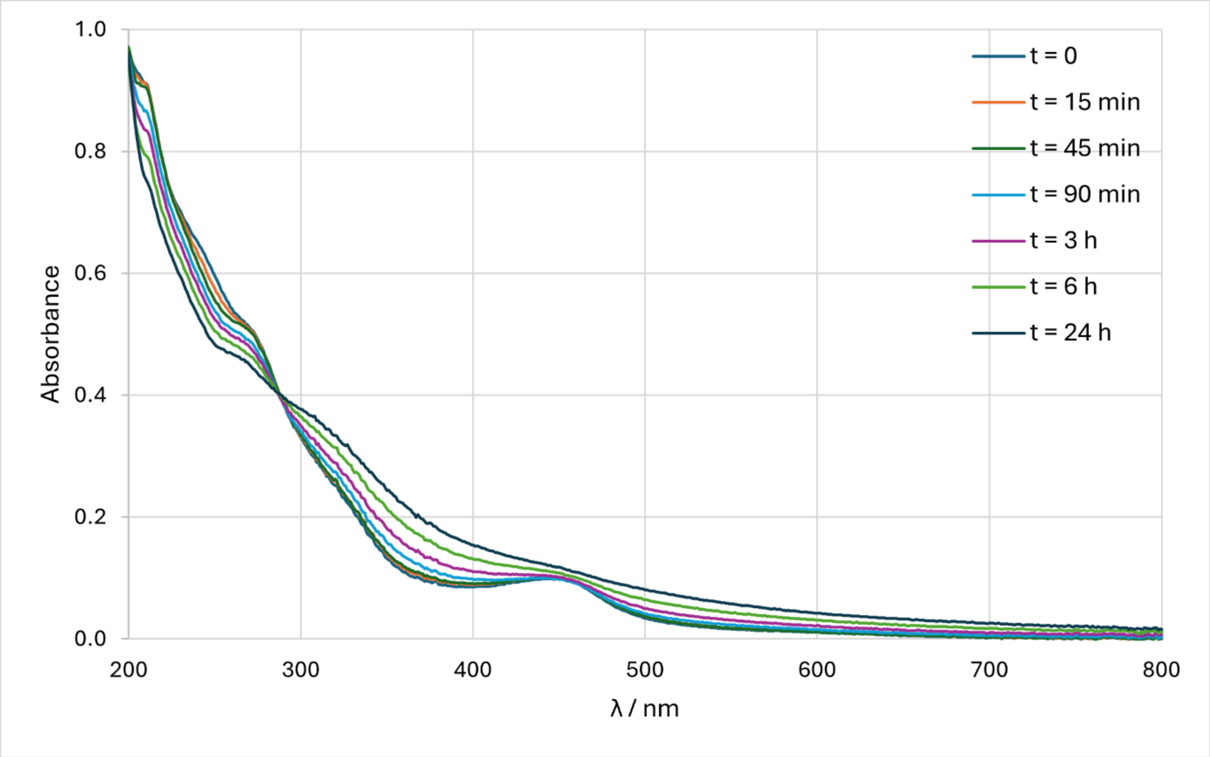

Figure S24. UV-visible spectra for compound **3-Co** (c = 40 μM in pentane, 298 K).

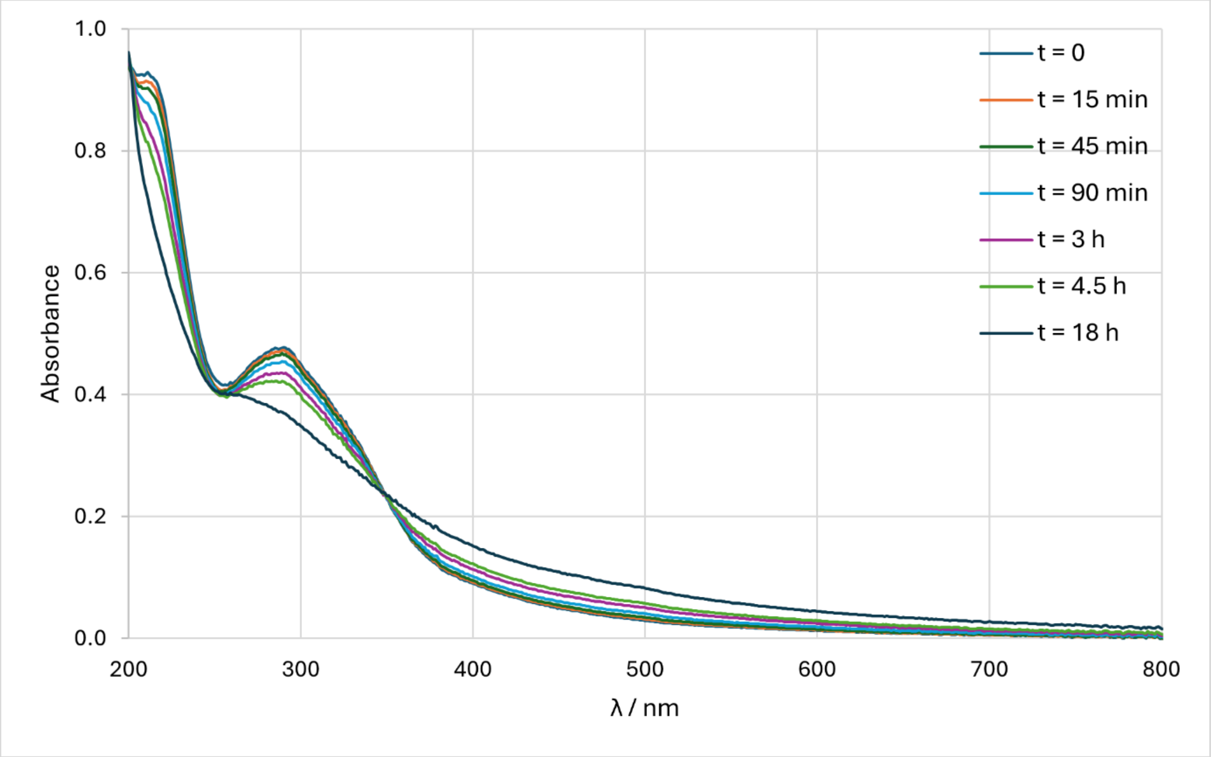

Figure S25. UV-visible spectra for compound **3-Ni** (c = 40 μM in pentane, 298 K).

Diffuse reflectance infrared Fourier-transform spectroscopy (DRIFTS) data for compounds **3-M** (M = Cr, Mn, Fe, Co, Ni)

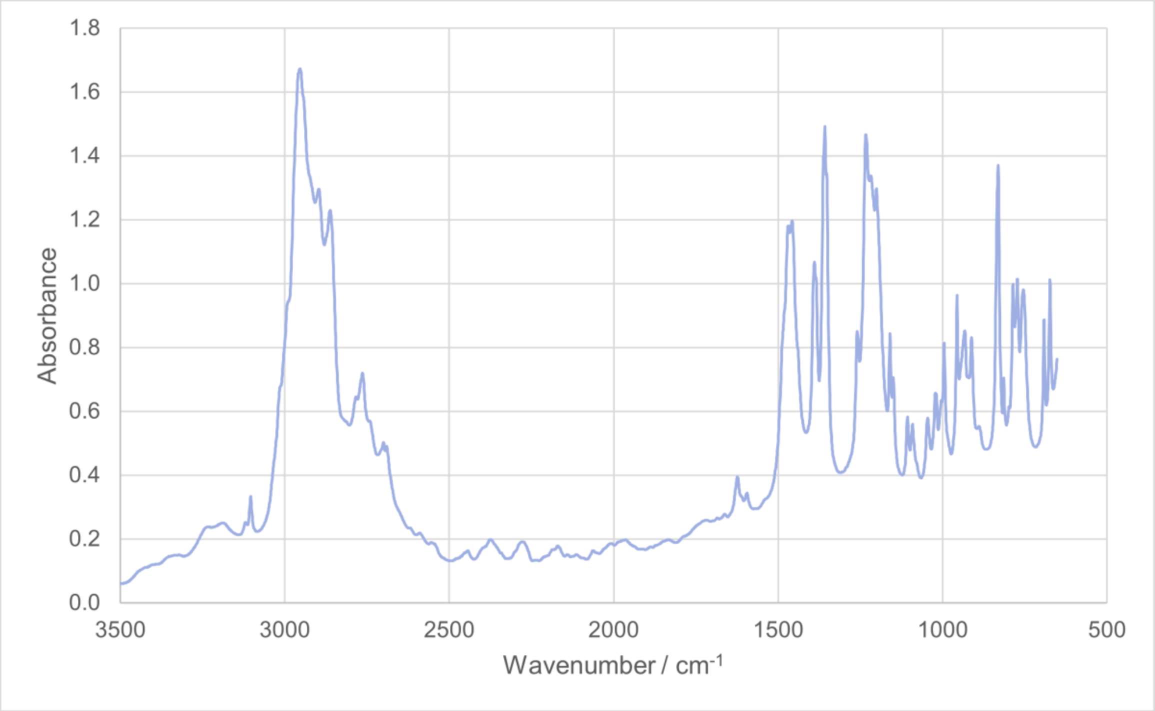

Figure S26. DRIFTS infrared spectrum for compound **3-Cr** (293 K).

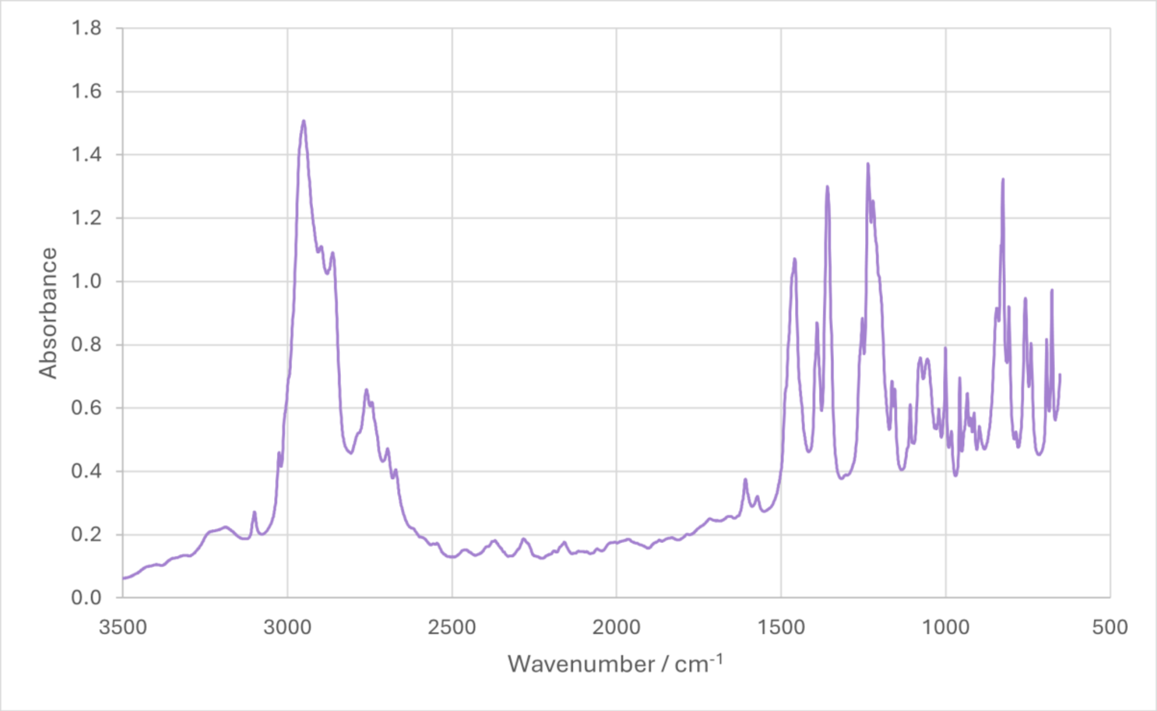

Figure S27. DRIFTS infrared spectrum for compound **3-Mn** (293 K).

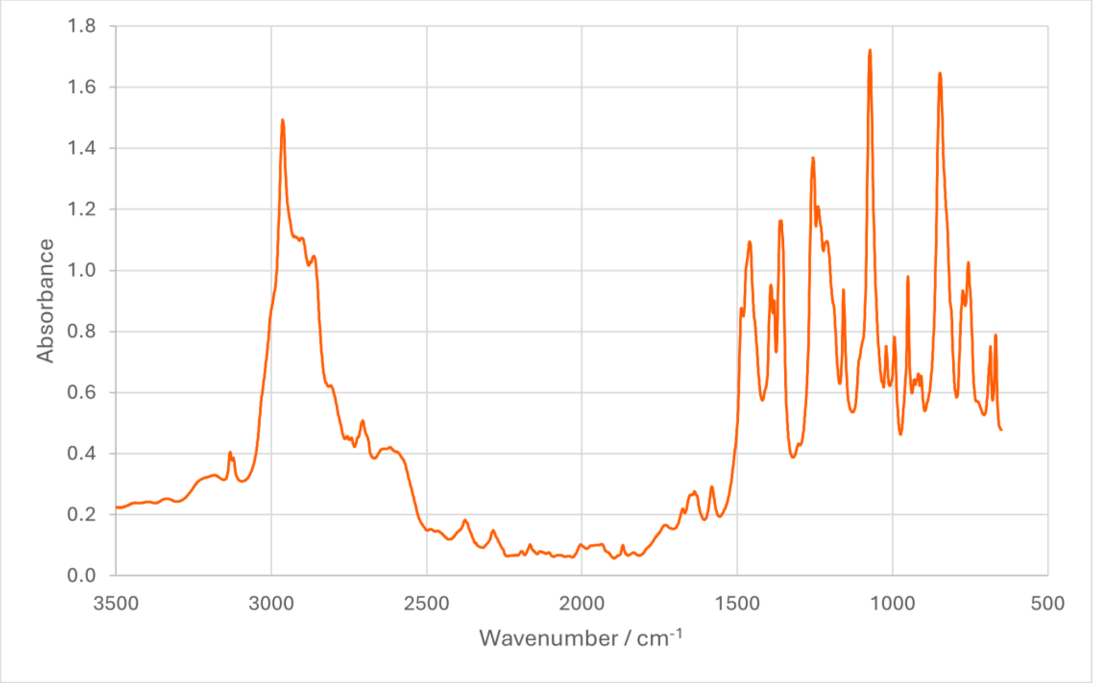

Figure S28. DRIFTS infrared spectrum for compound **3-Fe** (293 K).

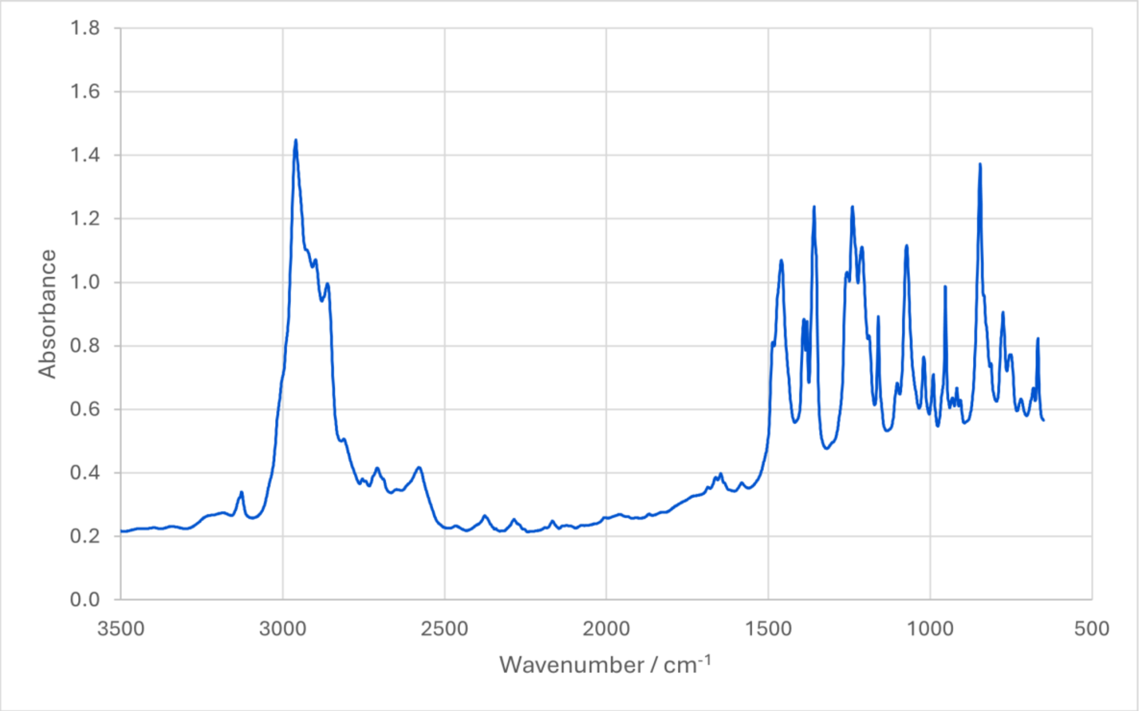

Figure S29. DRIFTS infrared spectrum for compound **3-Co** (293 K).

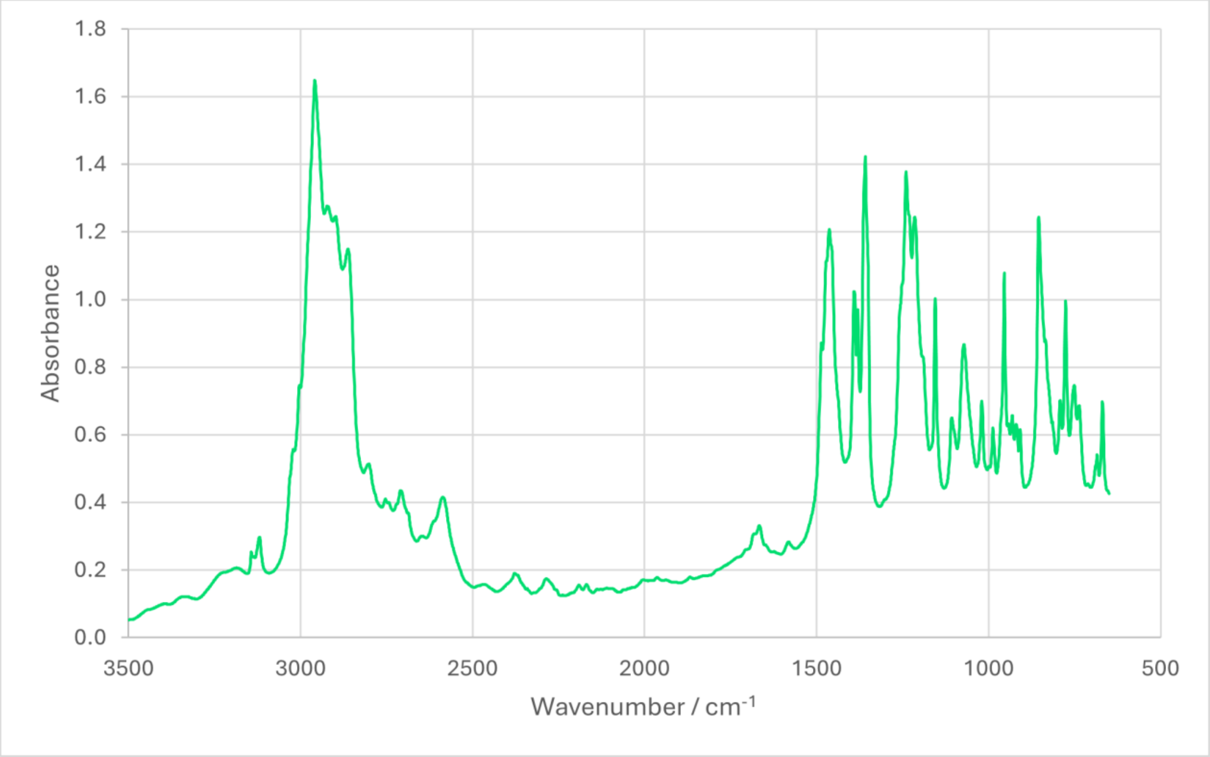

Figure S30. DRIFTS infrared spectrum for compound **3-Ni** (293 K).

Evans magnetometry for paramagnetic compounds **3-M** (M = Cr, Mn, Fe, Co)

General method: Sample solutions with known concentrations of each compound **3-M** (M = Cr, Mn, Fe, Co) in 0.600 mL deuterated benzene (C_6D_6) were prepared and transferred to J. Young NMR tubes. To these NMR samples, a sealed glass capillary containing (C_6D_6) was added for reference. A standard ^{1}H NMR experiment was run. The difference in chemical shift ($\Delta\nu$) between the residual solvent signals in the sample and in the reference capillary was used to estimate the effective magnetic moment (μ_{eff}) of each complex.[1]

- The measured molar magnetic susceptibility, χ_{M} (in cm^3 mol^{-1}), was calculated according to the equation

$$\chi_M = \frac{3000\Delta\nu}{4\pi\nu_0 c}$$

  where ν_0 denotes the spectrometer frequency (300.13 · 106 Hz), $\Delta\nu$ the difference in chemical shift between the residual solvent signals in Hz and c the sample concentration in mol L^{-1}.

- The paramagnetic contribution to the magnetic susceptibility, χ_{P}, was calculated using

$$\chi_P = \chi_M - \chi_D$$

  where the diamagnetic component, χ_{D}, was estimated using Pascal's constants.[2]

- μ_{eff} was then calculated using equation

$$\mu_{eff} = \sqrt{8\chi_P T}$$

  where T is the temperature at which the corresponding NMR acquisition was performed (293-297 K).

Table S1. Evans magnetometry data

Compound	$\Delta\nu$ / Hz	c / mol L^{-1}	χ_{D} / cm^3 mol^{-1}	χ_{P} / cm^3 mol^{-1}	μ_{eff} / μ_{B}	n
**3-Cr**	612.3	0.0512	-0.479 · 10^{-3}	9.994 · 10^{-3}	4.86	4.0
**3-Mn**	642.3	0.0350	-0.478 · 10^{-3}	15.065 · 10^{-3}	5.98	5.1
**3-Fe · ½ HMDSO**	369.2	0.0454	-0.533 · 10^{-3}	7.006 · 10^{-3}	4.08	3.2
**3-Co · ½ HMDSO**	81.0	0.0398	-0.488 · 10^{-3}	2.106 · 10^{-3}	2.23	1.4

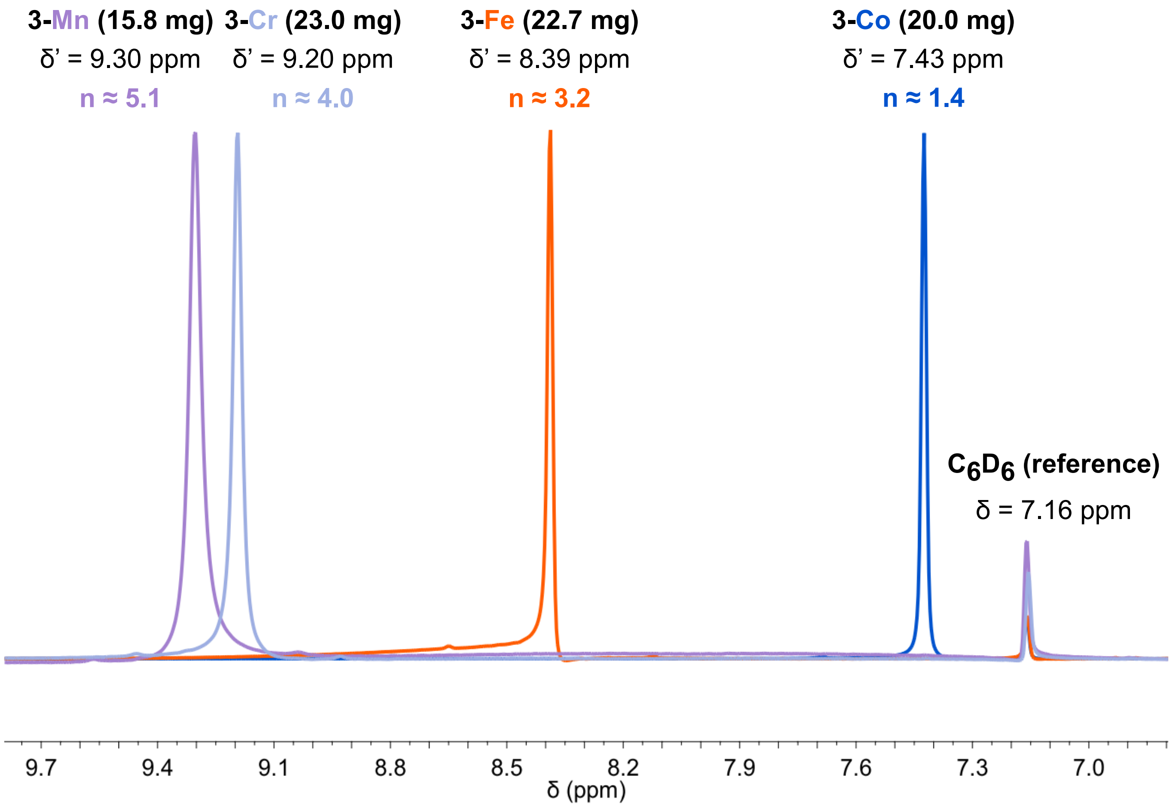

Figure S31. Evans ^{1}H NMR data.

These values were compared to the expected spin-only magnetic moment given by

$$\mu_{eff} = \sqrt{n(n+2)}\mu_B$$

where n is the number of unpaired electrons. It is obtained by solving the quadratic equation and choosing the positive solution for n.

Crystallographic data for compounds **3-M** (M = Cr, Mn, Fe, Co, Ni), **3-Mn'** and **3-Mn"**

Single crystal samples were prepared in a glove box under an argon atmosphere and transported to the diffractometer in a sealed scintillation vial. Suitable crystals were selected and mounted on a MITIGEN holder in perfluoroether oil on a XtaLAB Synergy, Dualflex, HyPix-Arc 100 diffractometer. The crystals were kept at a steady T = 100 K during data collection. The structure was solved with the ShelXT[3] solution program using dual methods and by using Olex2 1.5-ac6-020[4] as the graphical interface. The model was refined with ShelXL 2018/3[5] using full matrix least squares minimization on F2.

Table S2. Crystallographic parameters for compounds **3-Cr**, **3-Mn**, **3-Mn'** and **3-Mn"**

**Compound**	**3-Cr**	**3-Mn**	**3-Mn'**	**3-Mn''**
Formula	$C_{37}H_{71}CrTa$	$C_{37}H_{71}MnTa$	$C_{36}H_{74}I_2LiMnO_4Ta$	$C_{28}H_{58}I_2LiMnO_2Ta$
$D_{calc.}$ / g cm^{-3}	1.325	1.315	1.551	1.690
m / mm^{-1}	3.224	3.233	4.047	5.083
Formula Weight	748.88	751.82	1067.58	923.37
Color	dark brown	brown	orange	orange
Shape	plate-shaped	plate-shaped	plate-shaped	block-shaped
Size / mm^3	0.44×0.38×0.10	0.17×0.10×0.02	0.37×0.27×0.04	0.29×0.27×0.16
T / K	100.0(3)	100.00(10)	100.0(2)	99.97(19)
Crystal System	monoclinic	monoclinic	monoclinic	monoclinic
Space Group	$C2/c$	$I2/a$	$P2_1/c$	$P2_1/c$
a / Å	41.9672(3)	17.5557(2)	11.3713(2)	13.6057(2)
b / Å	10.29100(10)	10.26850(10)	36.6513(6)	15.61140(10)
c / Å	17.62920(10)	42.9593(5)	11.2345(2)	17.8463(2)
α / °	90	90	90	90
β / °	99.6440(10)	101.2280(10)	102.397(2)	106.8290(10)
γ / °	90	90	90	90
V / Å^3	7506.18(10)	7596.08(15)	4573.06(14)	3628.28(7)
Z	8	8	4	4
Z'	1	1	1	1
Wavelength / Å	0.71073	0.71073	0.71073	0.71073
Radiation type	Mo K_a	Mo K_a	Mo K_a	Mo K_a
Θ_{min} / °	1.969	2.309	2.378	3.209
Θ_{max} / °	30.462	30.416	30.643	30.623
Measured Refl's.	70939	14627	102927	82300
Indep't Refl's	10039	14627	12301	9905
Refl's I ≥ 2 σ(I)	9377	11823	11006	9378
R_{int}	0.0316	0.0580	0.0455	0.0316
Parameters	373	374	457	337
Restraints	0	198	173	2
Largest Peak	0.837	2.036	2.090	0.734
Deepest Hole	-0.777	-1.245	-3.282	-0.617
GooF	1.024	1.092	1.287	1.076
wR_2 (all data)	0.0442	0.1170	0.1203	0.0346
wR_2	0.0433	0.1052	0.1181	0.0342
R_1 (all data)	0.0210	0.0649	0.0646	0.0175
R_1	0.0185	0.0453	0.0567	0.0158

Table S3. Crystallographic parameters for compounds **3-Fe**, **3-Co** and **3-Ni**

**Compound**	**3-Fe**	**3-Co**	**3-Ni**
Formula	$C_{80}H_{160}Fe_2OSi_2Ta_2$	$C_{80}H_{160}Co_2OSi_2Ta_2$	$C_{80}H_{160}Ni_2OSi_2Ta_2$
$D_{calc.}$ / g cm^{-3}	1.283	1.298	1.295
m / mm^{-1}	2.921	2.993	3.039
Formula Weight	1667.85	1674.01	1673.57
Color	black	brown	black
Shape	block-shaped	plate-shaped	block-shaped
Size / mm^3	0.37×0.20×0.17	0.24×0.17×0.05	0.19×0.15×0.10
T / K	100.0(3)	100.01(10)	100.0(4)
Crystal System	monoclinic	monoclinic	monoclinic
Space Group	$P2/c$	$P2/c$	$P2/c$
a / Å	18.6705(3)	18.5937(2)	18.5082(3)
b / Å	11.7058(2)	11.6867(2)	11.7081(2)
c / Å	19.8286(3)	19.8027(2)	19.8941(3)
α / °	90	90	90
β / °	95.068(2)	95.5610(10)	95.503(2)
γ / °	90	90	90
V / Å^3	4316.66(12)	4282.85(10)	4291.10(12)
Z	2	2	2
Z'	0.5	0.5	0.5
Wavelength / Å	0.71073	0.71073	0.71073
Radiation type	Mo K_a	Mo K_a	Mo K_a
Θ_{min} / °	2.343	2.353	2.350
Θ_{max} / °	30.545	30.424	31.196
Measured Refl's.	62111	99617	262631
Indep't Refl's	11219	11341	12717
Refl's I ≥ 2 σ(I)	9766	9505	10981
R_{int}	0.0826	0.0504	0.0572
Parameters	432	432	431
Restraints	80	8	275
Largest Peak	3.423	2.050	2.532
Deepest Hole	-3.465	-1.374	-1.235
GooF	1.045	1.031	1.141
wR_2 (all data)	0.1299	0.0660	0.0795
wR_2	0.1265	0.0621	0.0753
R_1 (all data)	0.0652	0.0414	0.0443
R_1	0.0571	0.0298	0.0343

EPR spectroscopy

X-band cw-EPR spectra were recorded using a Bruker EMX+ spectrometer operating with a high quality factor Bruker 4122 SHQE resonator. Settings: HF frequency = 9.391 GHz, power = 0.2 mW, Modulation Amplitude = 10 G, T = 20K. The numerical simulation of EPR spectra was performed using EasySpin Matlab Library.[1]

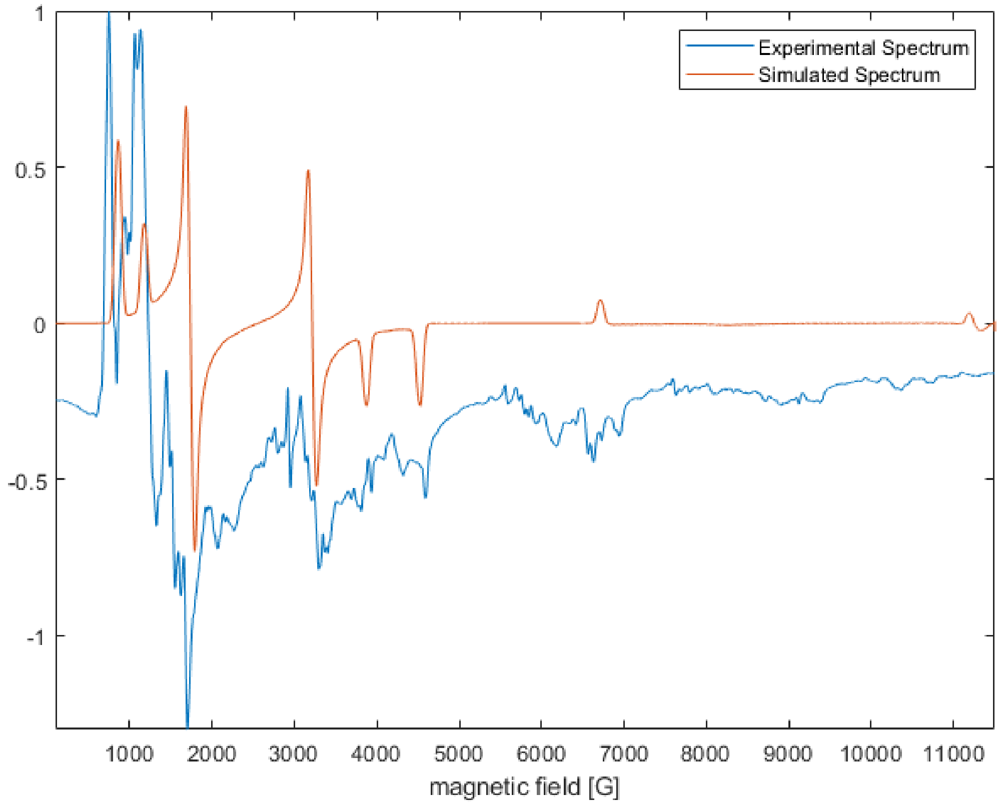

Figure S32. **X-band EPR spectrum of 3-M**. Experimental spectrum recorded at T=20K (in blue), numerical simulation (in red) with (D=-1.12 cm^{-1}, E=-0.10 cm^{-1}), using Easyspin Matlab Library (see below).

The EPR spectrum of the complex **3-M** (500 µM in toluene solution) was recorded in X-band at T=20 K. It exhibits a pattern that is consistent with a half-integer Kramer system of S=5/2 electronic spin in the low field regime. Indeed, DFT computations of the zero-field splitting (ZFS) parameters (D,E) of **3-M** yields a D value of -1.12 cm^{-1}, much higher than the energy of X band photons 0.3 cm^{-1}. In such a case the shape of the spectrum is mainly due the ZFS anisotropy. Accordingly, the simulation proposed below was obtained by exploring E values in the range 0 and 0.35 cm^{-1} while keeping D value constant at -1.12 cm^{-1}. The criterion for selecting the best simulation spectrum was its agreement with the experimental resonance fields

[1] S. Stoll and A. Schweiger , J. Magn. Reson., 2006, 178, 42-55.

of the most intense transitions observed in the range [680 G - 8000 G] (the weaker signals observed at higher magnetic field could not be simulated nor attributed). This resulted in a E value of -0.10 cm^{-1}, smaller but of comparable magnitude with the DFT computed one (-0.17 cm^{-1}). The hyperfine and quadrupolar interactions of ^{55}Mn and ^{181}Ta nuclei computed by DFT were then included in the simulation, which did not change the shape and resonance fields of the simulated spectrum as these interactions are of much lower intensity than the ZFS interaction in **3-M**.

Overall, the recorded EPR spectrum for **3-M** confirms its S=5/2 electronic spin and is in qualitative agreement with the DFT computed values for the ZFS interaction. This provides an experimental validation for the electronic and coordination structure of **3-M** obtained by DFT computations.

DFT methodology applied to EPR data. The DFT calculation of the ZFS tensor and derived parameters D and E has been performed with the ADF code[2] using the Generalized Gradient Approximation (GGA) VBP exchange-correlation (XC) potential [VWN + BP: Vosko, Wilk & Nusair[3] + corrective terms by Becke[4] for the exchange, and Perdew[5] for the correlation], and scalar relativistic option ("relativistic scalar ZORA").

DFT calculation of the g-tensor, as well as those of the hyperfine (for the Mn metal ion) and quadrupolar (for the Ta metal ion) tensors have been performed using the PBE0 functional (25% HF exchange) developed by Ernzerhof-Scuseria[6] and by Adamo-Barone[7], a hybrid form of PBE, combined with full spin-orbit option (« relativistic spinorbit ZORA »).

All calculations used triple-zeta (TZ2P) basis sets (option "core none"), and numerical precision 6. The geometry of the complex **3-M** used for the DFT calculations is that deduced from X-ray crystallography.

---

[2] Velde, G.; Baerends, E. Numerical-Integration for Polyatomic Systems. J. Comput. Phys. 1992, 99, 84.

[3] Vosko, S. H.; Wilk, L.; Nusair, M. Accurate Spin-Dependent Electron Liquid Correlation Energies for Local Spin Density Calculations: A Critical Analysis. Can. J. Phys. 1980, 58, 1200.

[4] Becke, A. D. Density-Functional Exchange-Energy Approximation with Correct Asymptotic Behavior. Phys. Rev. A 1988, 38, 3098.

[5] Perdew, J. P. Density-Functional Approximation for the Correlation Energy of the Inhomogeneous Electron Gas. Phys. Rev. B 1986, 33, 8822.

[6] M. Ernzerhof and G. Scuseria, Assessment of the Perdew.Burke.Ernzerhof exchange-correlation functional. Journal of Chemical Physics 110, 5029 (1999).

[7] C. Adamo and V. Barone, Toward reliable density functional methods without adjustable parameters: The PBE0 model. Journal of Chemical Physics 110, 6158 (1999).

I – g-tensor (PBE0 ; full spin-orbit relativistic)

==== full g-tensor

	X	Y	Z
X	2.004231	0.003453	-0.000363
Y	0.003453	2.004878	-0.002577
Z	-0.000363	-0.002577	2.001163

===== principal axes g-tensor

	11	22	33
X	-0.347426	0.717737	-0.603447
Y	0.567402	-0.351442	-0.744677
Z	0.746559	0.601117	0.285145

===== principal values g-tensor

g11	g22	g33
1.999373	2.002236	2.008663

Isotropic g-value: 2.003424

II – ZFS tensor (GGA ; scalar relativistic)

===== full D-tensor (ZFS) in original axis system (cm-1)

	X	Y	Z
X	-0.059653	0.010824	-0.516619
Y	0.010824	0.357295	-0.260314
Z	-0.516619	-0.260314	-0.297642

===== principal axes D-tensor (ZFS)

	11	22	33
X	0.672245	-0.446704	0.590374
Y	-0.649183	-0.739020	0.180033
Z	-0.355877	0.504287	0.786795

==== principal values D-tensor

D11	D22	D33	
0.203384E+00	0.541470E+00	-0.744854E+00	cm-1

ZFS D= -1.117281 cm-1, E= -0.169043 cm-1

III – Mn Hyperfine tensor (PBE0 ; full spin-orbit relativistic)

==== calculated tensor (a.u.) for Mn

	X	Y	Z
X	0.118117E-07	-0.332897E-10	0.285622E-09
Y	-0.332897E-10	0.114581E-07	-0.296356E-09
Z	0.285622E-09	-0.296356E-09	0.118947E-07

===== principal axes A-tensor Mn

	11	22	33
X	-0.223819	0.800750	0.555612
Y	0.831024	0.454642	-0.320468
Z	0.509219	-0.390000	0.767200

principal values 55Mn A-tensor (includes factor g_n/S)

a11	a22	a33	
0.412111E+02	0.425557E+02	0.446431E+02	MHz

Isotropic value 55Mn A-tensor: 42.8033 MHz

IV – Ta quadripolar tensor (PBE0 ; full spin-orbit relativistic)

==== EFG-tensor (a.u.) for Ta

	X	Y	Z
X	-0.293107E+00	0.537182E+00	-0.256881E+00
Y	0.537182E+00	-0.796797E+00	-0.313908E-01
Z	-0.256881E+00	-0.313908E-01	0.108990E+01

==== principal axes Q-tensor (EFG) Ta

	11	22	33
X	0.544110	0.814537	-0.201179
Y	-0.837474	0.541787	-0.071444
Z	0.050802	0.207356	0.976946

principal values EFG Ta

V11	V22	V33	
-0.114390E+01	-0.119655E-02	0.114510E+01	a.u.

principal values 181Ta Q-tensor (includes factor Q/(2I(2I-1)))

q11	q22	q33	
-0.202863E+02	-0.212199E-01	0.203075E+02	MHz

V – Mulliken Spin populations

Atom	Charge pop	Spin pop	spins	S	P	D	F
Ta	1.2228	0.1519	alpha	5.1673	12.1377	11.6132	7.0464
			beta	5.1638	12.1243	11.4799	7.0447
**Mn**	0.4333	**4.8088**	alpha	3.3743	6.4108	**4.8993**	0.0034
			beta	3.1002	6.2302	**0.5453**	0.0032

The spin population is clearly mainly localized on the Mn metallic ion.

Computational details

All DFT calculations were carried out with the Gaussian 16 suite of programs.[6] Geometries were fully optimized in the gas phase without symmetry constraints using the B3PW91 functional.[7,8] The nature of the extrema was verified by analytical frequency calculations. The calculations of electronic energies and enthalpies of the extrema of the potential energy surface (minima and transition states) were performed at the same level of theory as the geometry optimizations. Intrinsic reaction coordinate (IRC) calculations were performed to confirm the connections of the optimized transition states. Iron, cobalt, nickel, chromium, tantalum and manganese atoms were treated with a Stuttgart effective core potential augmented with a polarization function ($\zeta f = 2.462$, $\zeta f = 2.780$, $\zeta f = 3.130$, $\zeta f = 1.941$, $\zeta f = 0.790$ and $\zeta f = 2.195$ respectively for Fe, Co, Ni, Cr, Ta and Mn atoms).[9,10] For the other elements (H and C), Pople's double-ζ basis set 6-31G(d,p) was used.[11,12] The electronic charges (at the DFT level) were computed using the natural population analysis technique.[13] Dispersion corrections were treated with the D3 version of Grimme's dispersion with Becke-Johnson damping.[14]

The validity of the method used was tested by comparing the results of changing the size of the basis set (6-31G(d,p) vs. 6-311G(d,p)[15,16]) and also the functional (B3PW91 vs. PBE0[17,18]) on the stability of the different tautomers. As can be seen in Table S14, the energy differences when using a double-ζ or triple-ζ basis set are almost identical. The relative stability of the different complexes both in terms of preferred form and spin state remains the same and therefore the conclusions stay unchanged. As far as the effect of functional is concerned, for iron, nickel, manganese and chromium we obtain exactly the same trends with both PBE0 and B3PW91. However, for cobalt, with the PBE0 functional the B form is destabilized, which is not in agreement with the experimental results. Consequently, only the studies carried out with the B3PW91 functional and the Pople's double-ζ basis set 6-31G(d,p) for the hydrogen and carbon atoms will be discussed in the manuscript.

Table S4. Comparison of the experimental and theoretical key structural parameters (distances in Å and angles in deg) for complexes **3-Cr**. From a theoretical point of view, only the most stable structures have been considered. Cp' denotes the centroid of the carbon atoms making up the five-membered ring of the cyclopentadienyl ligand.

**M = Cr**		Without Dispersion	With Dispersion
	Exp. (B - W)	B – W quintet	B – W quintet
Ta···M	2.8484(3)	2.915	2.843
Ta−C1	2.163(2)	2.163	2.162
Ta−C2	2.145(2)	2.174	2.159
Ta−C3	2.010(2)	2.021	2.014
Ta−C4	1.995(2)	2.013	2.001
M−C3	2.249(2)	2.308	2.256
M−C4	2.323(2)	2.350	2.305
M···Cp'	2.017(3)	2.076	2.026
Ta−C1−tBu	129.5(2)	134.5	125.0
Ta−C2−tBu	133.2(2)	130.3	128.4
Ta−C3−tBu	121.1(1)	124.4	120.7
Ta−C4−tBu	133.1(1)	132.2	131.7
C1−Ta−C2	114.24(7)	113.0	115.8
C1−Ta−C3	113.25(7)	106.4	113.7
C1−Ta−C4	114.06(7)	108.5	113.7
C2−Ta−C3	107.53(7)	114.6	107.2
C2−Ta−C4	106.44(7)	113.4	105.0
C3−Ta−C4	100.13(7)	100.0	99.8
C3−M−C4	84.40(6)	83.1	84.6
C3−M−Cp'	137.19(6)	138.3	137.3
C4−M−Cp'	136.51(5)	136.8	136.3

Table S5. Comparison of the experimental and theoretical key structural parameters (distances in Å and angles in deg) for complexes **3-Mn**. From a theoretical point of view, only the most stable structures have been considered. Cp' denotes the centroid of the carbon atoms making up the five-membered ring of the cyclopentadienyl ligand.

**M = Mn**		Without Dispersion	With Dispersion
	Exp. (A)	A sextet	A sextet
Ta···M	3.017(2)	3.083	2.953
Ta−C1	2.16(2)	2.178	2.170
Ta−C2	2.16(2)	2.174	2.172
Ta−C3	1.89(2)	2.214	1.882
Ta−C4	2.20(2)	1.900	2.190
M−C3	2.96(2)	2.130	2.920
M−C4	2.16(2)	3.149	2.117
M···Cp'	2.05(2)	2.085	2.072
Ta−C1−tBu	134.5(8)	134.3	128.0
Ta−C2−tBu	133.3(8)	136.6	129.4
Ta−C3−tBu	154.2(9)	120.5	155.2
Ta−C4−tBu	120.1(8)	149.8	117.1
C1−Ta−C2	104.3(5)	105.2	106.6
C1−Ta−C3	110.9(5)	113.0	104.0
C1−Ta−C4	110.8(5)	106.6	112.4
C2−Ta−C3	107.5(5)	110.2	109.4
C2−Ta−C4	111.0(5)	109.7	114.4
C3−Ta−C4	112.0(5)	111.7	109.4
C3−M−C4	81.2(4)	77.9	81.0
C3−M−Cp'	161.6(3)	164.2	160.3
C4−M−Cp'	116.5(3)	117.4	118.3

Table S6. Comparison of the experimental and theoretical key structural parameters (distances in Å and angles in deg) for complexes **3-Fe**. From a theoretical point of view, only the most stable structures have been considered. Cp' denotes the centroid of the carbon atoms making up the five-membered ring of the cyclopentadienyl ligand.

**M = Fe**		Without Dispersion			With Dispersion		
	Exp. (B - ∩)	A quintet	C quintet	B - W quintet	A quintet	B – ∩ quintet	B – ∩ triplet
Ta···M	2.6406(8)	3.017	3.312	3.201	2.918	2.793	2.679
Ta−C1	2.174(6)	2.157	1.915	2.171	2.179	2.167	2.153
Ta−C2	2.071(6)	2.159	2.188	2.191	2.167	2.173	2.114
Ta−C3	2.017(7)	2.196	2.193	1.935	2.181	1.963	2.038
Ta−C4	2.055(5)	1.887	2.113	2.114	1.890	2.035	2.053
M−C3	2.066(7)	2.157	2.069	2.080	2.957	2.677	2.059
M−C4	2.105(6)	2.965	3.604	3.708	2.041	2.156	2.058
M···Cp'	1.878(7)	1.990	1.954	1.965	1.961	2.022	1.891
Ta−C1−tBu	126.2(4)	133.3	168.7	135.1	128.8	122.0	124.7
Ta−C2−tBu	161.4(4)	134.5	129.5	129.1	129.5	129.5	154.1
Ta−C3−tBu	136.4(5)	120.1	129.5	157.7	116.9	159.5	128.4
Ta−C4−tBu	128.5(4)	154.2	129.5	123.0	151.2	133.1	127.9
C1−Ta−C2	108.9(2)	104.3	110.8	113.0	108.4	112.1	107.4
C1−Ta−C3	106.7(3)	111.0	106.7	106.4	114.0	108.2	106.4
C1−Ta−C4	112.3(2)	107.5	115.4	108.5	108.8	110.8	109.1
C2−Ta−C3	114.8(3)	110.2	106.0	114.6	110.3	109.8	118.6
C2−Ta−C4	115.0(2)	110.8	109.1	113.4	104.2	108.1	117.1
C3−Ta−C4	98.7(3)	112.0	108.3	100.0	110.7	107.9	92.5
C3−M−C4	95.6(3)	81.2	70.1	83.1	82.0	83.2	96.6
C3−M−Cp'	132.5(2)	167.3	169.9	159.7	114.8	138.9	128.3
C4−M−Cp'	129.4(2)	115.3	128.8	132.6	157,0	132.0	128.7

Table S7. Comparison of the experimental and theoretical key structural parameters (distances in Å and angles in deg) for complexes **3-Co**. From a theoretical point of view, only the most stable structures have been considered. Cp' denotes the centroid of the carbon atoms making up the five-membered ring of the cyclopentadienyl ligand.

**M = Co**		Without Dispersion			With Dispersion		
	Exp. (B - ∩)	B - W doublet	B - W quartet	A quartet	B - ∩ doublet	B - W quartet	B - ∩ quartet
Ta···M	2.6196(4)	2.659	2.842	3.027	2.642	2.724	2.720
Ta−C1	2.169(3)	2.155	2.171	2.167	2.157	2.170	2.162
Ta−C2	2.086(3)	2.172	2.182	2.175	2.112	2.164	2.173
Ta−C3	2.053(3)	2.022	2.002	2.176	2.050	2.010	2.024
Ta−C4	2.020(3)	2.040	2.037	1.910	2.030	2.003	1.978
M−C3	2.077(3)	2.168	2.130	1.945	2.028	2.133	2.201
M−C4	2.060(3)	2.147	2.666	3.307	2.027	2.454	2.570
M···Cp'	1.793(3)	1.968	1.985	1.908	1.826	1.972	1.977
Ta−C1−tBu	126.2(2)	140.9	135.6	130.7	124.3	127.8	123.1
Ta−C2−tBu	161.4(2)	131.3	132.8	129.0	155.2	129.6	129.7
Ta−C3−tBu	127.4(2)	129.8	129.3	120.9	127.6	133.1	131.5
Ta−C4−tBu	134.5(2)	132.7	133.0	145.0	129.2	127.6	158.0
C1−Ta−C2	108.0(1)	111.4	110.2	98.4	107.1	112.8	111.4
C1−Ta−C3	113.2(1)	106.7	103.0	109.9	110.8	114.9	110.9
C1−Ta−C4	106.5(1)	106.5	106.2	104.4	107.4	117.2	107.7
C2−Ta−C3	114.9(1)	116.8	117.0	121.5	117.0	106.5	106.8
C2−Ta−C4	115.1(1)	116.7	114.7	104.5	117.4	100.5	110.1
C3−Ta−C4	98.8(1)	97.2	104.6	115.6	107.4	103.4	109.8
C3−M−C4	96.8(2)	89.9	82.6	77.7	97.4	86.4	86.4
C3−M−Cp'	129.54(9)	135.3	133.2	172.5	128.8	141.2	129.1
C4−M−Cp'	131.3(2)	134.7	143.4	105.4	129.1	132.2	138.2

Table S8. Comparison of the experimental and theoretical key structural parameters (distances in Å and angles in deg) for complexes **3-Ni**. From a theoretical point of view, only the most stable structures have been considered. Cp' denotes the centroid of the carbon atoms making up the five-membered ring of the cyclopentadienyl ligand.

**M = Ni**		Whitout Dispersion		With Dispersion
	Exp. (B - ∩)	A triplet	B - ∩ singlet	B - ∩ singlet
Ta···M	2.5773(4)	3.077	2.646	2.612
Ta−C1	2.157(3)	2.173	2.172	2.152
Ta−C2	2.081(3)	2.165	2.130	2.117
Ta−C3	2.035(3)	2.199	2.039	2.042
Ta−C4	2.019(3)	1.929	2.050	2.028
M−C3	2.050(3)	1.966	2.022	1.998
M−C4	2.030(3)	3.377	2.026	1.996
M···Cp'	1.819(3)	1.869	1.889	1.852
Ta−C1−tBu	127.5(2)	134.0	128.9	124.7
Ta−C2−tBu	162.0(2)	139.3	155.7	154.2
Ta−C3−tBu	128.3(2)	123.0	129.0	127.8
Ta−C4−tBu	133.3(2)	143.2	129.6	127.8
C1−Ta−C2	108.5(1)	104.5	107.5	107.6
C1−Ta−C3	112.6(1)	110.7	109.6	109.3
C1−Ta−C4	106.6(1)	107.2	106.3	106.5
C2−Ta−C3	114.8(1)	107.3	117.3	117.5
C2−Ta−C4	115.3(1)	111.3	119.4	119.1
C3−Ta−C4	98.6(1)	115.0	95.8	96.0
C3−M−C4	97.8(2)	76.4	97.1	98.4
C3−M−Cp'	129.5(1)	170.2	129.8	128.9
C4−M−Cp'	131.1(2)	111.3	129.6	128.9

Table S9. Computational relative electronic enthalpies ($\Delta_r H$) and Gibbs free energies ($\Delta_r G$) of complex **3-Cr** in the tautomeric forms A to C taking into account the different possible spin states and including or not dispersion effects.

**M = Cr**	Spin Multiplicity	$\Delta_r G$ (kcal/mol)	$\Delta_r H$ (kcal/mol)	$\Delta_r G$ (kcal/mol)	$\Delta_r H$ (kcal/mol)
		Without Dispersion		With Dispersion	
**A**	singlet	53.9	51.9	--	--
	triplet	22.6	21.7	20.8	18.4
	quintet	0.0	0.0	0.0	0.0
**C**	singlet	63.0	60.2	--	--
	triplet	32.9	31.6	37.1	35.7
	quintet	8.4	10.0	12.3	12.3
**B**	singlet (W)	40.1	34.9	38.8	34.7
	singlet (∩)	--	--	63.8	61.2
	singlet (Z)	45.7	41.5	--	--
	triplet (W)	23.4	20.0	23.2	21.2
	triplet (∩)	30.0	27.1	--	--
	triplet (Z)	24.7	20.8	--	--
	quintet (W)	-2.7	-4.1	-3.6	-4.2
	quintet (∩)	-0.5	-2.0	-3.2	-1.9
	quintet (Z)	-2.0	-2.8	--	--

Table S10. Computational relative electronic enthalpies (Δ_rH) and Gibbs free energies (Δ_rG) of complex **3-Mn** in the tautomeric forms A to C taking into account the different possible spin states and including or not dispersion effects.

**M = Mn**	Spin Multiplicity	Δ_rG (kcal/mol) Without Dispersion	Δ_rH (kcal/mol) Without Dispersion	Δ_rG (kcal/mol) With Dispersion	Δ_rH (kcal/mol) With Dispersion
**A**	doublet	53.0	48.8	--	--
	quartet	25.5	24.6	23.5	23.3
	sextet	0.0	0.0	0.0	0.0
**C**	doublet	56.8	55.9	--	--
	quartet	43.4	42.8	43.9	42.6
	sextet	3.4	4.0	6.8	6.3
				--	--
**B**	doublet (W)	45.6	41.8	51.0	48.5
	doublet (∩)	51.1	46.5	53.8	50.9
	doublet (Z)	47.6	42.9	--	--
	quartet (W)	23.9	21.6	32.8	30.5
	quartet (∩)	24.3	20.5	28.6	26.6
	quartet (Z)	26.3	23.0	--	--
	sextet (W)	4.0	3.8	5.0	4.5
	sextet (∩)	4.9	4.6	3.7	3.3
	sextet (Z)	3.3	3.5	--	--

Table S11. Computational relative electronic enthalpies (Δ_rH) and Gibbs free energies (Δ_rG) of complex **3-Fe** in the tautomeric forms A to C taking into account the different possible spin states and including or not dispersion effects.

**M = Fe**	Spin Multiplicity	Δ_rG (kcal/mol)	Δ_rH (kcal/mol)	Δ_rG (kcal/mol)	Δ_rH (kcal/mol)
		Without Dispersion		With Dispersion	
**A**	singlet	45.5	43.1	--	--
	triplet	16.9	16.8	16.3	15.6
	quintet	0.0	0.0	0.0	0.0
**C**	singlet	52.3	49.5	--	--
	triplet	38.9	39.3	29.2	27.9
	quintet	1.1	2.4	6.1	5.6
**B**	singlet (W)	40.3	37.1	49.2	47.0
	singlet (∩)	42.3	38.4	38.2	34.5
	singlet (Z)	40.5	36.7	--	--
	triplet (W)	9.3	8.3	14.0	11.4
	triplet (∩)	14.3	11.9	12.1	9.6
	triplet (Z)	11.6	8.8	--	--
	quintet (W)	2.0	2.7	4.8	4.8
	quintet (∩)	8.5	9.6	3.3	3.1
	quintet (Z)	4.4	5.9	--	--

Table S12. Computational relative electronic enthalpies ($\Delta_r H$) and Gibbs free energies ($\Delta_r G$) of complex **3-Co** in the tautomeric forms A to C taking into account the different possible spin states and including or not dispersion effects.

**M = Co**	Spin Multiplicity	$\Delta_r G$ (kcal/mol) Without Dispersion	$\Delta_r H$ (kcal/mol) Without Dispersion	$\Delta_r G$ (kcal/mol) With Dispersion	$\Delta_r H$ (kcal/mol) With Dispersion
**A**	doublet	0.0	0.0	8.6	9.0
	quartet	0.1	1.8	0.0	0.0
	sextet	23.9	25.8	--	--
**C**	doublet	6.9	7.9	25.4	25.4
	quartet	-13.2	-10.7	4.9	6.1
	sextet	19.1	22.1	--	--
**B**	doublet (W)	-7.5	-8.0	6.8	5.6
	doublet (∩)	-10.0	-12.1	0.4	-1.4
	doublet (Z)	-9.3	-11.3	--	--
	quartet (W)	-15.1	-12.7	0.1	0.5
	quartet (∩)	-10.1	-8.7	1.3	1.8
	quartet (Z)	-11.8	-11.5	--	--
	sextet (W)	20.1	23.4	37.1	40.0
	sextet (∩)	26.4	29.6	48.0	51.3
	sextet (Z)	20.8	23.5	--	--

Table S13. Computational relative electronic enthalpies ($\Delta_r H$) and Gibbs free energies ($\Delta_r G$) of complex **3-Ni** in the tautomeric forms A to C taking into account the different possible spin states and including or not dispersion effects.

**M = Ni**	Spin Multiplicity	$\Delta_r G$ (kcal/mol) Without Dispersion	$\Delta_r H$ (kcal/mol) Without Dispersion	$\Delta_r G$ (kcal/mol) With Dispersion	$\Delta_r H$ (kcal/mol) With Dispersion
**A**	singlet	8.4	6.4	5.4	3.6
	triplet	0.0	0.0	0.0	0.0
	quintet	40.5	41.2	--	--
**C**	singlet	23.0	21.6	20.1	18.3
	triplet	1.6	3.8	7.1	6.8
	quintet	45.3	47.3	--	--
**B**	singlet (W)	5.3	3.2	3.8	1.9
	singlet (∩)	2.2	-1.4	-3.7	-6.3
	singlet (Z)	4.4	1.3	--	--
	triplet (W)	2.0	2.5	2.1	2.4
	triplet (∩)	4.6	5.3	5.2	5.3
	triplet (Z)	5.3	4.0	--	--
	quintet (W)	36.7	38.4	55.3	56.8
	quintet (∩)	42.8	44.3	51.2	52.4
	quintet (Z)	38.8	40.2	--	--

Table S14. Computational relative electronic enthalpies (ΔrH) and Gibbs free energies (ΔrG) of complex **3-M** in the tautomeric forms A to C taking into account a different basis set and functional. Energies are in kcal/mol.

	Spin Multiplicity	ΔrG B3PW91/6-31G(d,p)	ΔrH	ΔrG B3PW91/6-311G(d,p)	ΔrH	ΔrG PBE0/6-311G(d,p)	ΔrH
**M = Cr**							
A	triplet	20.8	19.4	21.0	19.5	22.6	22.8
	quintet	0.0	0.0	0.0	0;0	0.0	0.0
B	triplet	23.2	21.2	23.3	20.7	23.7	21.0
	quintet	-3.6	-4.8	-3.9	-4.9	-4.4	-5.5
C	triplet	37.1	35.7	35.0	33.9	39.6	38.8
	quintet	12.3	12.3	11.9	12.1	10.8	11.3
**M = Mn**							
A	quartet	23.5	23.3	23.9	23.8	26.1	26.9
	sextet	0.0	0.0	0.0	0.0	0.0	0.0
B	quartet	28.6	26.6	24.9	22.7	26.1	24.1
	sextet	3.7	3.3	3.5	3.3	3.1	4.3
C	quartet	43.9	42.6	39.7	39.4	47.6	47.0
	sextet	6.8	6.3	6.7	6.4	5.9	5.8
**M = Fe**							
A	triplet	16.3	15.6	16.8	16.0	21.1	20.1
	quintet	0.0	0.0	0.0	0.0	0.0	0.0
B	triplet	12.1	9.6	17.4	14.9	14.4	11.4
	quintet	3.3	3.1	3.0	3.0	5.1	5.6
C	triplet	29.2	27.9	32.1	32.4	31.4	30.8
	quintet	6.1	5.6	5.9	5.6	5.1	5.6
**M = Co**							
A	doublet	8.6	9.0	9.1	9.4	18.9	18.7
	quartet	0.0	0.0	0.0	0.0	0.0	0.0
B	doublet	0.4	-1.4	0.4	-1.6	4.5	2.5
	quartet	1.3	1.8	3.4	5.4	2.2	2.6
C	doublet	25.4	25.4	25.6	25.7	23.4	23.4
	quartet	4.9	6.1	5.1	6.1	4.0	5.4
**M = Ni**							
A	singlet	5.4	3.6	4.8	3.1	10.5	8.7
	triplet	0.0	0.0	0.0	0.0	0.0	0.0
B	singlet	-3.7	-6.3	-4.0	-6.8	0.5	-2.6
	triplet	5.2	5.3	4.8	4.7	4.8	4.8
C	singlet	20.1	18.3	20.1	18.2	26.0	24.5
	triplet	7.1	6.8	7.1	6.8	6.1	6.1

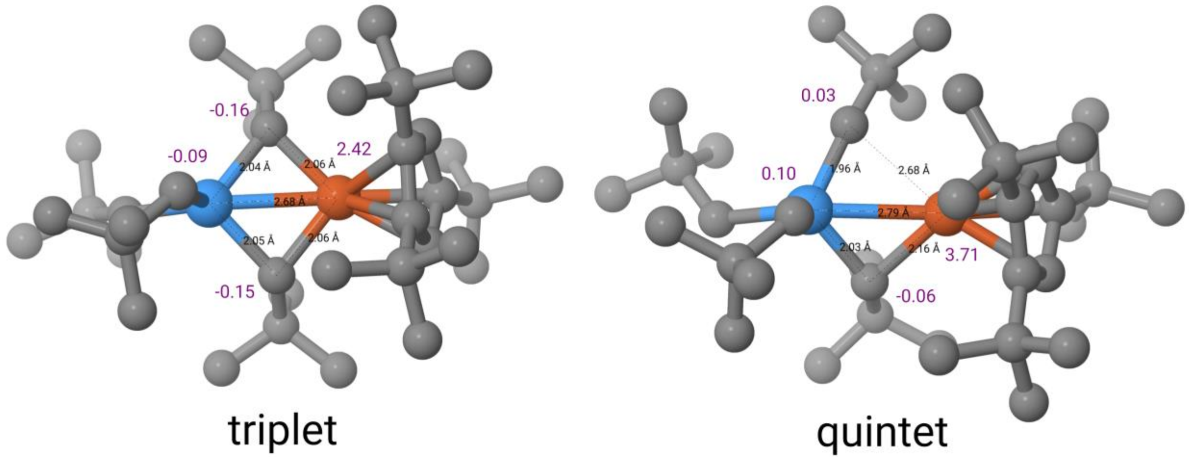

Figure S33. Comparison the key distances (black value) and spin densities (violet value) for triplet and quintet spin states of **3-Fe**.

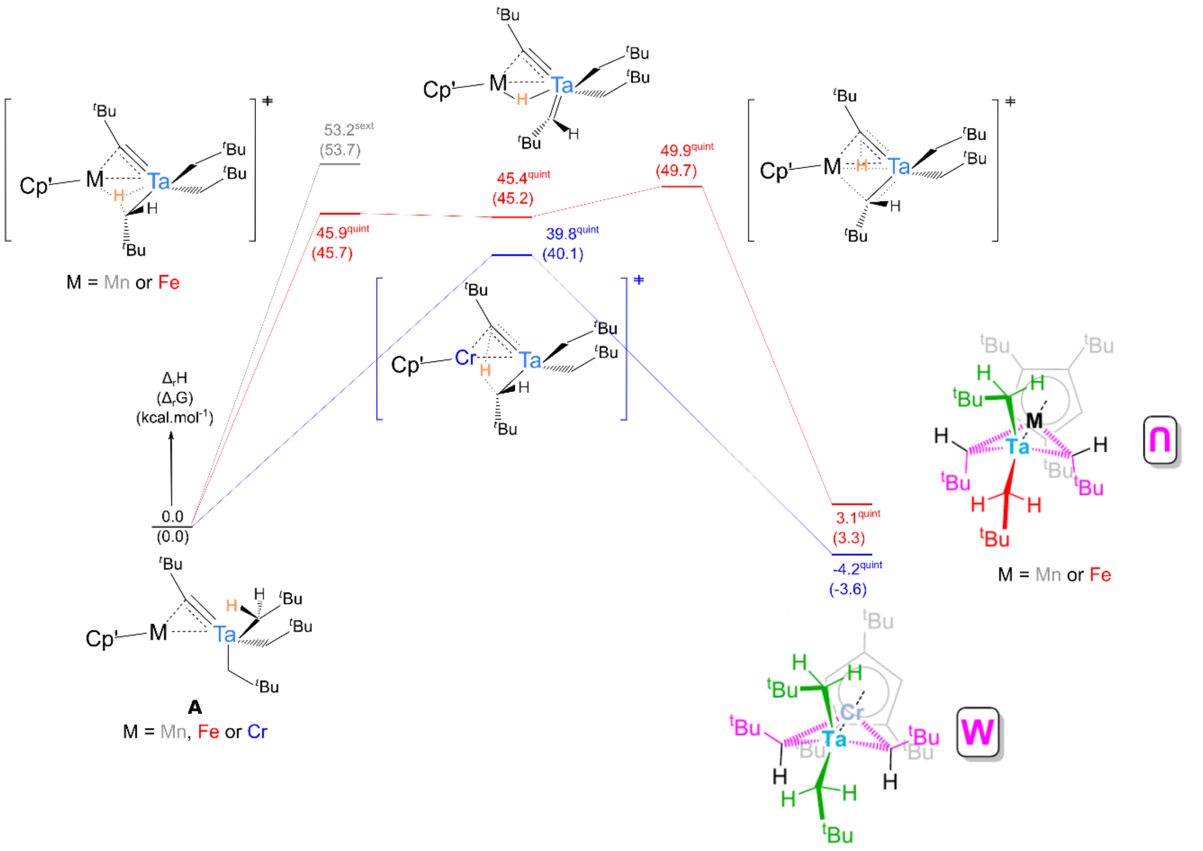

Figure S34. Computed energy profile at room temperature for the tautomerism reaction A ⇌ B for compounds **3-M** (M = Cr, Mn, Fe). The energies are given in kcal/mol. The spin state for the transition states and products are given in superscript. Energy profiles were not calculated for **3-Co** and **3-Ni** because the preferred spin states for tautomers A and B are not the same according to the DFT results (see above).

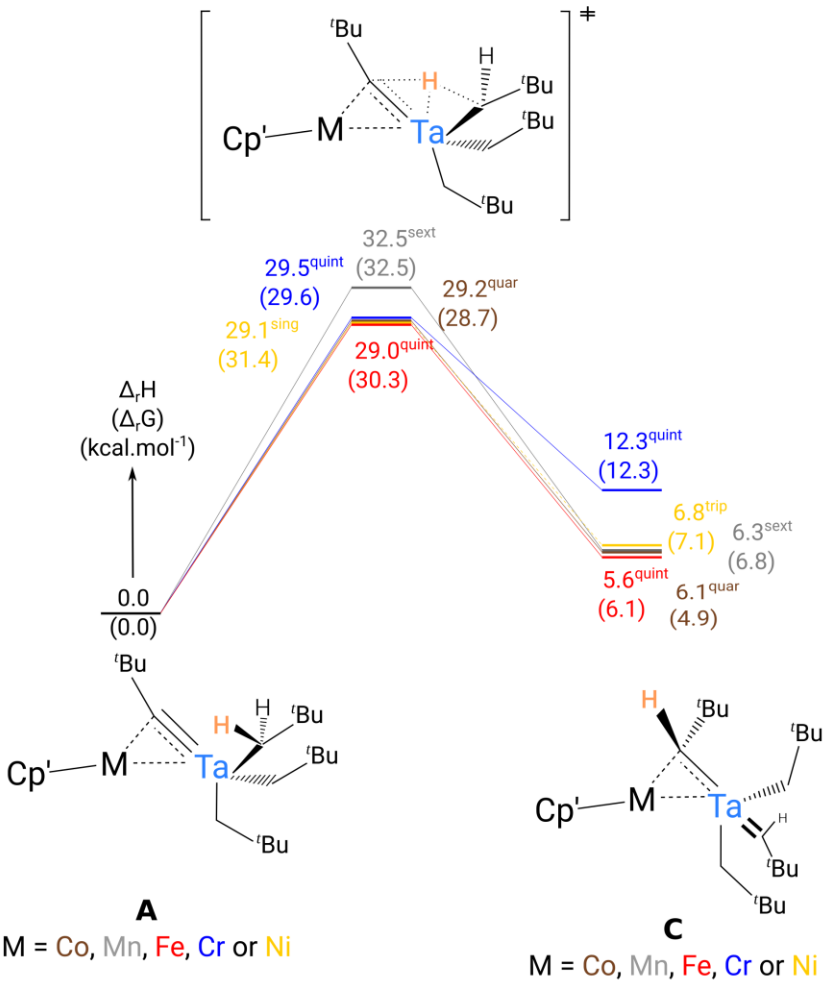

Figure S35. Computed energy profile at room temperature for the tautomerism reaction A ⇌ C. The energies are given in kcal/mol. The spin state for the transition states and C products are given in superscript.

References

(1) Evans, D. F. 400. The Determination of the Paramagnetic Susceptibility of Substances in Solution by Nuclear Magnetic Resonance. *J. Chem. Soc.* **1959**, 2003. https://doi.org/10.1039/jr9590002003.

(2) Bain, G. A.; Berry, J. F. Diamagnetic Corrections and Pascal's Constants. *J. Chem. Educ.* **2008**, *85* (4), 532. https://doi.org/10.1021/ed085p532.

(3) Sheldrick, G. M. SHELXT – Integrated Space-Group and Crystal-Structure Determination. *Acta Cryst. A* **2015**, *71* (1), 3–8. https://doi.org/10.1107/S2053273314026370.

(4) Dolomanov, O. V.; Bourhis, L. J.; Gildea, R. J.; Howard, J. A. K.; Puschmann, H. OLEX 2: A Complete Structure Solution, Refinement and Analysis Program. *J. Appl. Crystallogr.* **2009**, *42* (2), 339–341. https://doi.org/10.1107/S0021889808042726.

(5) Sheldrick, G. M. Crystal Structure Refinement with SHELXL. *Acta Cryst. C* **2015**, *71* (1), 3–8. https://doi.org/10.1107/S2053229614024218.

(6) M. J. Frisch, G. W. Trucks, H. B. Schlegel, G. E. Scuseria, M. A. Robb,; J. R. Cheeseman, G. Scalmani, V. Barone, G. A. Petersson, H. Nakatsuji, X. Li,; M. Caricato, A. V. Marenich, J. Bloino, B. G. Janesko, R. Gomperts, B. Mennucci,; H. P. Hratchian, J. V. Ortiz, A. F. Izmaylov, J. L. Sonnenberg, D. Williams Young, F. Ding, F. Lipparini, F. Egidi, J. Goings, B. Peng, A. Petrone, T. Hen derson, D. Ranasinghe, V. G. Zakrzewski, J. Gao, N. Rega, G. Zheng, W. Liang,; M. Hada, M. Ehara, K. Toyota, R. Fukuda, J. Hasegawa, M. Ishida, T. Nakajima,; Y. Honda, O. Kitao, H. Nakai, T. Vreven, K. Throssell, J. A. Montgomery, Jr.,; J. E. Peralta, F. Ogliaro, M. J. Bearpark, J. J. Heyd, E. N. Brothers, K. N. Kudin,; V. N. Staroverov, T. A. Keith, R. Kobayashi, J. Normand, K. Raghavachari,; A. P. Rendell, J. C. Burant, S. S. Iyengar, J. Tomasi, M. Cossi, J. M. Millam,; M. Klene, C. Adamo, R. Cammi, J. W. Ochterski, R. L. Martin, K. Morokuma,; O. Farkas, J. B. Foresman, and D. J. Fox. Gaussian 16, Revisions B.01v and B.01, Gaussian Inc., Wallingford, CT, 2016.

(7) Becke, A. D. Density-Functional Thermochemistry. III. The Role of Exact Exchange. *J. Chem. Phys.* **1993**, *98* (7), 5648–5652. https://doi.org/10.1063/1.464913.

(8) Burke, K.; Perdew, J. P.; Wang, Y. Derivation of a Generalized Gradient Approximation: The PW91 Density Functional. In *Electronic Density Functional Theory*; Dobson, J. F., Vignale, G., Das, M. P., Eds.; Springer US: Boston, MA, 1998; pp 81–111. https://doi.org/10.1007/978-1-4899-0316-7_7.

(9) Dolg, M.; Wedig, U.; Stoll, H.; Preuss, H. Energy-Adjusted *Ab Initio* Pseudopotentials for the First Row Transition Elements. *J. Chem. Phys.* **1987**, *86* (2), 866–872. https://doi.org/10.1063/1.452288.

(10) Ehlers, A. W.; Böhme, M.; Dapprich, S.; Gobbi, A.; Höllwarth, A.; Jonas, V.; Köhler, K. F.; Stegmann, R.; Veldkamp, A.; Frenking, G. A Set of F-Polarization Functions for Pseudo-Potential Basis Sets of the Transition Metals Sc-Cu, Y-Ag and La-Au. *Chem. Phys. Lett.* **1993**, *208* (1–2), 111–114. https://doi.org/10.1016/0009-2614(93)80086-5.

(11) Hariharan, P. C.; Pople, J. A. The Influence of Polarization Functions on Molecular Orbital Hydrogenation Energies. *Theoret. Chim. Acta* **1973**, *28* (3), 213–222. https://doi.org/10.1007/BF00533485.

(12) Hehre, W. J.; Ditchfield, R.; Pople, J. A. Self-Consistent Molecular Orbital Methods. XII. Further Extensions of Gaussian-Type Basis Sets for Use in Molecular Orbital Studies of Organic Molecules. *J. Chem. Phys.* **1972**, *56* (5), 2257–2261. https://doi.org/10.1063/1.1677527.

(13) Reed, A. E.; Curtiss, L. A.; Weinhold, F. Intermolecular Interactions from a Natural Bond Orbital, Donor-Acceptor Viewpoint. *Chem. Rev.* **1988**, *88* (6), 899–926. https://doi.org/10.1021/cr00088a005.

(14) Grimme, S.; Ehrlich, S.; Goerigk, L. Effect of the Damping Function in Dispersion Corrected Density Functional Theory. *J. Comput. Chem.* **2011**, *32* (7), 1456–1465. https://doi.org/10.1002/jcc.21759.

(15) McLean, A. D.; Chandler, G. S. Contracted Gaussian Basis Sets for Molecular Calculations. I. Second Row Atoms, *Z*=11–18. *J. Chem. Phys.* **1980**, *72* (10), 5639–5648. https://doi.org/10.1063/1.438980.

(16) Krishnan, R.; Binkley, J. S.; Seeger, R.; Pople, J. A. Self-Consistent Molecular Orbital Methods. XX. A Basis Set for Correlated Wave Functions. *J. Chem. Phys.* **1980**, *72* (1), 650–654. https://doi.org/10.1063/1.438955.

(17) Adamo, C.; Barone, V. Toward Reliable Density Functional Methods without Adjustable Parameters: The PBE0 Model. *J. Chem. Phys.* **1999**, *110* (13), 6158–6170. https://doi.org/10.1063/1.478522.

(18) Ernzerhof, M.; Perdew, J. P. Generalized Gradient Approximation to the Angle- and System-Averaged Exchange Hole. *J. Chem. Phys.* **1998**, *109* (9), 3313–3320. https://doi.org/10.1063/1.476928.
